# Platinum(0)-η^2^-1,2-(*E*)ditosylethene Complexes Bearing Phosphine, Isocyanide and *N*-Heterocyclic Carbene Ligands: Synthesis and Cytotoxicity towards Ovarian and Breast Cancer Cells

**DOI:** 10.3390/molecules29051119

**Published:** 2024-03-01

**Authors:** Nicola Compagno, Rachele Piccolo, Enrica Bortolamiol, Nicola Demitri, Flavio Rizzolio, Fabiano Visentin, Thomas Scattolin

**Affiliations:** 1 Department of Molecular Sciences, Nanosystems Università Ca’ Foscari, Campus Scientifico, Via Torino 155, 30174 Venezia, Italy; nicola.compagno@unive.it (N.C.); enrica.bortolamiol@unive.it (E.B.); flavio.rizzolio@unive.it (F.R.); 2 Area Science Park Elettra-Sincrotrone Trieste, S.S. 14 Km 163.5, Basovizza, 34149 Trieste, Italy; 3 Pathology Unit, Department of Molecular Biology and Translational Research, Centro di Riferimento Oncologico di Aviano (CRO), IRCCS, Via Franco Gallini 2, 33081 Aviano, Italy; 4 Dipartimento di Scienze Chimiche, Università degli Studi di Padova, Via Marzolo 1, 35131 Padova, Italy

**Keywords:** platinum(0) complexes, alkene ligands, phosphine and isocyanide ligands, metallodrugs, *N*-heterocyclic carbenes, ovarian and breast cancer

## Abstract

A wide range of platinum(0)-η^2^-(*E*)-1,2-ditosylethene complexes bearing isocyanide, phosphine and *N*-heterocyclic carbene ancillary ligands have been prepared with high yields and selectivity. All the novel products underwent thorough characterization using spectroscopic techniques, including NMR and FT-IR analyses. Additionally, for some compounds, the solid-state structures were elucidated through X-ray diffractometry. The synthesized complexes were successively evaluated for their potential as anticancer agents against two ovarian cancer cell lines (A2780 and A2780*cis*) and one breast cancer cell line (MDA-MB-231). The majority of the compounds displayed promising cytotoxicity within the micromolar range against A2780 and MDA-MB-231 cells, with IC_50_ values comparable to or even surpassing those of cisplatin. However, only a subset of compounds was cytotoxic against cisplatin-resistant cancer cells (A2780*cis*). Furthermore, the assessment of antiproliferative activity on MRC-5 normal cells revealed certain compounds to exhibit in vitro selectivity. Notably, complexes **3d**, **6a** and **6b** showed low cytotoxicity towards normal cells (IC_50_ > 100 µM) while concurrently displaying potent cytotoxicity against cancer cells.

## 1. Introduction

Platinum(II) anticancer agents have been at the forefront of cancer treatment since the introduction of cisplatin in the 1970s [1]. These compounds usually exert their anticancer action by forming covalent DNA adducts, disrupting DNA replication and transcription and inducing apoptosis in rapidly dividing cancer cells [2]. The success of cisplatin has paved the way for the development of several platinum(II) derivatives (e.g., carboplatin and oxaliplatin), each with unique properties and therapeutic profiles [3].

Beyond conventional chemotherapy, platinum(II) complexes are being explored for their potential in combination therapies and immunotherapy [4]. The immunomodulatory effects of platinum drugs, including their impact on the tumor microenvironment, have prompted investigations into their role in enhancing the immune response against cancer cells [5]. This intersection of platinum-based chemotherapy with emerging immunotherapeutic strategies represents a promising avenue for improving overall treatment outcomes.

The clinical success of platinum(II) anticancer drugs, particularly cisplatin, has been accompanied by unavoidable challenges concerning the dose-limiting side effects, acquired resistance and limitations in their spectrum of activity [6]. For these reasons, researchers are actively engaged in developing strategies to overcome these issues. Among them, the design of novel platinum complexes with modified ligands to enhance selectivity and reduce toxicity represents a natural development [7].

A very interesting option is constituted by the study of platinum complexes in an oxidation state other than +2. In this context, numerous studies have been carried out on platinum complexes in a high oxidation state (IV).

Platinum(IV) anticancer agents represent a promising and evolving class of compounds that have garnered attention for their distinct properties and potential advantages over traditional platinum(II) drugs [8]. In fact, some Pt(IV) complexes exhibit enhanced stability, reduced side effects and the ability to release active platinum(II) species preferentially within cancer cells. These features contribute to making them candidates for the development of more effective and targeted anticancer therapies.

One notable characteristic of Pt(IV) complexes is their ability to undergo reduction within the cellular environment. Inside cancer cells, Pt(IV) compounds are reduced to their active Pt(II) form, which then forms DNA adducts, leading to DNA damage and ultimately triggering cell death [9,10]. This reduction process is believed to be at the basis of the preference for Pt(IV) complexes for cancer cells with the consequent lowering of the systemic toxicity.

A very limited number of works have instead concerned the evaluation of the anti-tumor activity of platinum complexes in low oxidation states.

In particular, only a couple of works on this topic have been reported in the last years by our group [11] and by Ruffo and colleagues [12]. In these contributions, the cytotoxicity of platinum(0) complexes bearing 1,3,5-triaza-7-phosphaadamantane (PTA) or *N*-heterocyclic carbenes (NHCs) as ancillary ligands and commercially available alkenes such as dimethyl fumarate, maleic anhydride and fumaronitrile was investigated. A promising antiproliferative activity towards ovarian cancer and myelogenous leukemia cell lines was observed for these compounds [11].

It should be pointed out that platinum(0) alkene complexes represent a fascinating class of coordination compounds that have gained significant attention in the field of organometallic chemistry. These compounds feature a platinum center bearing one or more alkene ligands, forming stable and well-defined structures with diverse applications in catalysis and materials science [13,14].

The binding of alkene ligands to platinum centers typically involves the formation of π-bonding interactions between the metal and the unsaturated carbon–carbon double bond of the alkene [13]. This interaction is enforced by the back-donation of electron density from the platinum d orbitals to the π* molecular orbital of the alkene, resulting in a synergistic relationship that stabilizes the complex.

One of the key features of platinum alkene complexes is their versatility in catalyzing different organic reactions, including hydrogenation, hydroamination and polymerization reactions [15,16,17]. The ability of platinum to engage in oxidative addition and reductive elimination processes allows these complexes to participate in catalytic cycles with high efficiency.

In addition to their catalytic applications, platinum alkene complexes have found use in the design of novel materials. For instance, the incorporation of these complexes into polymers can impart unique properties such as enhanced conductivity, making them valuable in the development of advanced electronic devices and sensors [18].

In this work, we report the synthesis, characterization and in vitro anticancer activity of novel platinum(0) complexes bearing different ancillary ligands (phosphines, isocyanides and *N*-heterocyclic carbenes (NHCs)) and (*E*)-1,2-ditosylethene as the model olefin. The choice of this olefin ligand is based on the fact that only olefins featuring strong electron-withdrawing substituents can ensure adequate stability for zero-valent platinum complexes, thus averting their rapid hydrolysis in a biological medium [19,20].

## 2. Results

### 2.1. Synthesis and Characterization of Pt(0)-η^2^-(E)-1,2-Ditosylethene Complexes

The platinum(0)–alkene complexes were obtained through a two-step synthetic approach, which involves the isolation of [(6-methyl-2-(methylthiomethyl)pyridine)Pt(η^2^-(*E*)-1,2-ditosylethene)] (**1**) as an intermediate species. From this derivative, it is possible to easily remove the chelating pyridyl–thioether ligand and introduce the two ancillary ligands of interest. The ease with which this process occurs has already been highlighted by our research group in previous works, describing ligand substitution reactions on palladium(0) complexes [21]. In these studies, it was demonstrated that the lability of the 6-methyl-2-(methylthiomethyl)pyridine ligand is ascribable to the distortion of the chelated ring with respect to the planarity, due to the steric interference of the methyl group in position 6 of the pyridine.

**Scheme 1:**
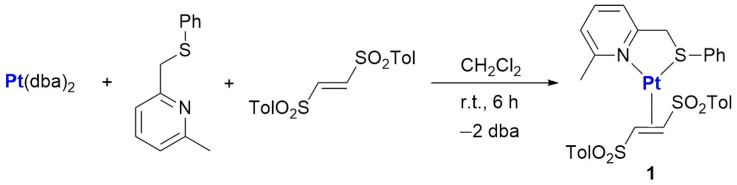
Synthesis of Pt(0) intermediate **1**.

More in detail, the [(6-methyl-2-(methylthiomethyl)pyridine)Pt(η^2^-(*E*)-1,2-ditosylethene)] (**1**) intermediate was prepared by reacting the pyridyl–thioether ligand, (*E*)-1,2-ditosylethene and Pt(dba)_2_ in dichloromethane at room temperature for ca. 6 h (Scheme 1). 

The product is stable in the solid state for months, and in chlorinated solvents, it is sufficiently stable to allow for an exhaustive characterization by NMR spectroscopy. In particular, in the ^1^H-NMR spectrum recorded at 298 K, the signals of the coordinated pyridyl–thioether ligand are present at lower chemical shifts than those of the free ligand. Furthermore, the methylene protons originate an AB system after the bidentate binding of the ligand on the platinum(0) center. 

Notably, all signals present in the ^1^H-NMR spectrum recorded at 298 K are significantly broadened, thus indicating a fluxional nature of the system. By lowering the temperature to 233 K, it is possible to observe the doubling of many signals, giving rise to two distinct groups of signals that can be ascribed to two different species.

The two species are two atropoisomers that differ from each other due to the different mutual positioning of the olefin tosyl substituents and the phenyl group bound to the thioether sulfur atom. At room temperature, the rapid inversion of the configuration of sulfur makes the two isomers indistinguishable. Conversely, at 233 K, this movement is impeded.

**Scheme 2:**
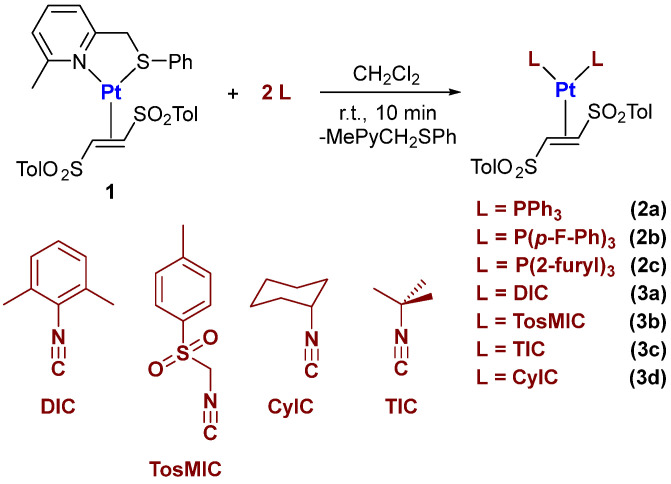
General procedure for the synthesis of bisphosphino and bisisocyanide Pt(0)-(*E*)-1,2-ditosylethene complexes.

Starting from intermediate **1**, the addition of two equivalents of an aryl phosphine or two equivalents of an isocyanide led to the formation of bisphosphino and bisisocyanide complexes **2a**–**c** and **3a**–**d**, respectively (Scheme 2). The reactions were carried out at room temperature in anhydrous dichloromethane for 10 min. The target products were isolated in high yields by precipitation from diethyl ether–*n*-pentane mixtures.

Importantly, most complexes can also be obtained directly by addition of the phosphine or isocyanide ligand, (*E*)-1,2-ditosylethene and Pt(dba)_2_ under similar operating conditions. However, the presence of traces of impurities in some complexes, as well as the need to develop a protocol valid also for platinum(0)–bisNHC complexes and mixed phosphine–isocyanide platinum(0) complexes convinced us to adopt the passage through intermediate **1** in all cases examined in this work.

As far as concerned the bisphosphino complexes **2a**–**c**, the ^31^P NMR spectra are all characterized by the presence of a single signal, accompanied by the two satellites due to the ^195^Pt-^31^P coupling (J ≈ 3700 Hz), which unequivocally testifies to the coordination of the phosphines occurring towards the platinum center. It should be noted that although the coordination of PPh_3_ and P(4-F-Ph)_3_ results in an increase in the chemical shift of the signal compared to that observed in the free phosphine (Δδ ≈ 30ppm), the coordination of tri(2-furyl)-phosphine entails a significant high-field shifting (Δδ ≈ −19 ppm). In the ^1^H and ^13^C NMR spectra, in addition to the expected signals attributable to the phosphine, even those characteristic of (*E*)-1,2-ditosylethene are all well identifiable. In particular, olefinic protons and carbons generate a characteristic AA’BB’ system due to the coupling with phosphorus nuclei.

Even in the case of bisisocyanide complexes **3a**–**d**, the ^1^H and ^13^C NMR spectra are rather simple considering the high symmetry of the compounds. In particular, all the signals related to isocyanide ligands can be identified, among which the coordinated isocyanide carbon, located at 130–140 ppm, is particularly diagnostic.

Moreover, the coordination of (*E*)-1,2-ditosylethene is confirmed by the presence of singlets ascribable to olefinic protons and carbons, which are localized at lower chemical shifts with respect to those observed in the free olefin because of metal-olefin backdonation.

**Scheme 3:**
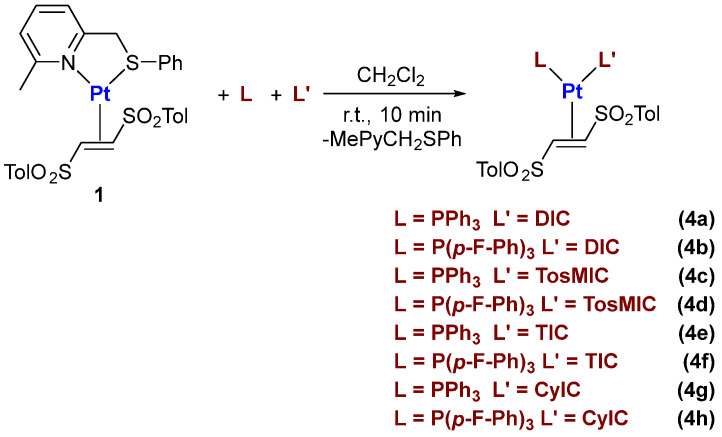
General procedure for the synthesis of Pt(0)-(*E*)-1,2-ditosylethene complexes bearing one phosphine and one isocyanide ligand.

With the aim of combining the peculiar characteristics of phosphine and isocyanide ligands, we have also successfully carried out the synthesis of Pt-η^2^-(*E*)-1,2-ditosylethene complexes bearing one phosphine and one isocyanide. In fact, the addition of one equivalent of each ligand to a dichloromethane solution of the pyridyl–thioether complex (**1**) selectively provides mixed phosphine–isocyanide complexes **4a**–**h** (Scheme 3). 

The only exception is represented by the complexes with tri(2-furyl)-phosphine, where the presence of the bisphosphino and bisisocyanide complexes was also observed.

**Scheme 4:**
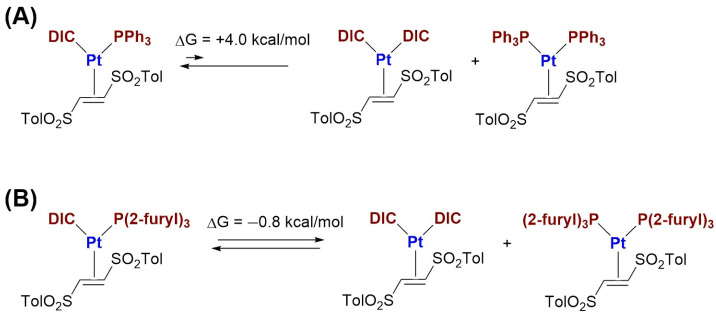
(**A**) Equilibrium characteristics of mixed PPh_3_–DIC, bisPPh_3_ and bisDIC platinum(0) complexes; (**B**) Equilibrium characteristics of mixed P(2-furyl)_3_–DIC, bisP(2-furyl)_3_ and bisDIC platinum(0) complexes.

To confirm this evidence, DFT calculations were carried out using [(PPh_3_)(DIC)Pt(η^2^-(*E*)-1,2-ditosylethene)] and [(P(2-furyl)_3_)(DIC)Pt(η^2^-(*E*)-1,2-ditosylethene)] as model complexes (Scheme 4A,B). 

The equilibrium of these species with their bisphosphino and bisisocyanide congeners is completely shifted towards the mixed phosphine–isocyanide complex in the case of triphenylphosphine (ΔG = +4.0 kcal·mol^−1^). Conversely, in the case of tri(2-furyl)-phosphine, the similar energy between the three complexes represented in Scheme 4B (ΔG = −0.8 kcal·mol^−1^) justifies the impossibility of selectively isolating the complex [(P(2-furyl)_3_)(DIC)Pt(η^2^-(*E*)-1,2-ditosylethene)].

Complexes **4a**–**h** were successfully characterized by NMR and IR analyses. In particular, in the ^31^P NMR spectra, a single signal at 23–26 ppm (ca. 30 ppm higher than that of the free phosphine) is detected. This fact, in conjunction with the presence of two satellites due to the coupling between the phosphorus and platinum nuclei, attests to the successful coordination of the phosphine ligand on the metal center. Moreover, in the ^31^P NMR spectra of platinum complexes bearing P(*p*-F-Ph)_3_, the signal appears as a quartet, due to the long-range coupling between the phosphorus and fluorine nuclei.

The coordination of the isocyanide ligand is generally confirmed by the presence in the ^13^C NMR spectra of a weak signal of the isocyanide carbon at ca. 150 ppm in the case of aryl isocyanides (DIC and TosMIC) and at ca. 135 ppm for alkyl isocyanides (TIC and CyIC). As usual, the coordination of (*E*)-1,2-ditosylethene is unequivocally attested by the presence, both in the ^1^H and ^13^C NMR spectra, of two distinct signals attributable to olefin protons and carbons. This differentiation of the signals, which is due to the two different ancillary ligands, gives rise to a system of a rather complex multiplicity, especially in the ^1^H NMR spectra, due to the simultaneous presence of H-H, H-P and H-Pt couplings. 

Finally, the IR spectra of all the synthesized complexes exhibit three characteristic signals: (i) CN stretching at 2100–2200 cm^−1^, (ii) SO stretching at 1300–1500 cm^−1^ and (iii) bending of the sulfonic group at 670 cm^−1^.

The last category of complexes that was examined in this work presents two *N*-heterocyclic carbene ligands (NHC) coordinated to the metal center.

*N*-heterocyclic carbene (NHC) ligands have emerged as pivotal components in modern organometallic chemistry, revolutionizing catalytic processes and enhancing the versatility of transition metal complexes. Since their discovery in the 1980s, NHC ligands have garnered significant attention owing to their unique electronic and steric properties, which make them ideal candidates for a wide range of applications [22]. 

**Scheme 5:**
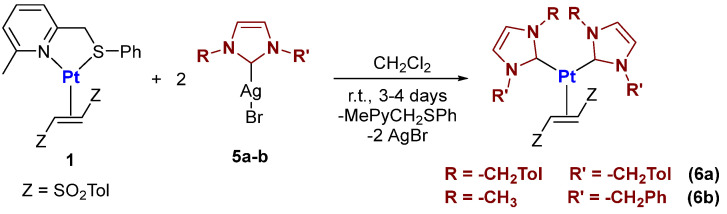
General procedure for the synthesis of Pt(0)-(*E*)-1,2-ditosylethene complexes bearing *N*-heterocyclic carbene ligands.

From some preliminary tests, it emerged that the most favorable synthetic protocol for the synthesis of [(NHC)_2_Pt(0)(η^2^-(*E*)-1,2-ditosylethene)] complexes is the transmetallation route. Given the well-known inertia of Pt(0) in ligand substitution reactions compared to Pd(0), we opted for two moderately bulky carbene ligands (see Scheme 5). In particular, two equivalents of the silver–NHC complexes **5a**,**b** were added to complex **1** in dichloromethane at room temperature. The transmetallation proceeds very slowly, requiring 3–4 days to complete the process.

After the filtration of AgBr, complexes **6a**,**b** were obtained in good yields by precipitation from a diethyl ether–*n*-pentane mixture.

The identity of the products was verified by ^1^H and ^13^C NMR analyses. In particular, the coordination of *N*-heterocyclic carbene ligands is proven by the presence in the ^13^C NMR spectra of the characteristic peak of the coordinated carbene carbon at ca. 180 ppm. In this case, the interaction with the metal center is further confirmed by the two satellites of the main signal, from which a *J*_Pt-C_ of ca. 1360 Hz can be inferred. 

Both in the ^1^H and ^13^C NMR spectra, the other signals of the carbene ligands can be easily identified and are located at different chemical shifts compared to those of the corresponding Ag-NHC precursors.

Notably, the olefinic carbons fall at very low chemical shifts (ca. 48 ppm), thus experimentally demonstrating the superior σ-donor character of *N*-heterocyclic carbene ligands with respect to aryl phosphines or isocyanides.

**Figure 1:**
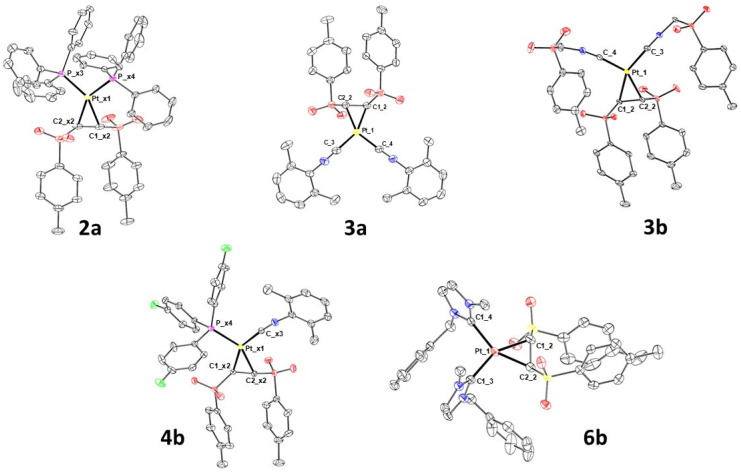
X-ray molecular structures of **2a**, **3a**, **3b**, **4b** and **6b** are presented, showing thermal displacement ellipsoids at the 50% probability level with hydrogen atoms, counterions and solvent molecules omitted for clarity.

To complete the characterization of the complexes covered by this work, the structures of complexes **2a**, **3a**, **3b**, **4b** and **6b** have been resolved by single-crystal X-ray diffraction (Figure 1).

Complexes **2a**, **3a**, **3b**, **4b** and **6b** molecules have been crystallized, characterized through XRD and show square planar platinum coordination spheres (Tables S1–S7). All the **3a**, **3b** and **6b** crystalline forms bear one crystallographically independent neutral platinum complex each, whereas **2a** and **4b** show two molecules in the asymmetric unit. The complex **3a** is isomorphic with the homologous palladium variant previously published (CCDC 1940848) [19]. A small coordination geometry adjustment reflects the change in the metallic center with a slight contraction of the metal–CN bond upon replacement of Pd with Pt, as previously reported [23]. Complexes bearing the same ligands are well overlapped with minor adjustment of phenyl sidechains of phosphine and tosyl ligands. Superimposition of crystallographically independent molecules bearing a (*Z*)-1,2-ditosylethene ligand shows equivalent conformations with an overall R.M.S.D. among common atoms less than ~1.7 Å. Sulfonic groups are involved in polar intermolecular contacts, but crystal packings are mainly stabilized by multiple weak hydrophobic intermolecular π···π and CH···π interactions among neighbor aromatic rings. 

### 2.2. Antiproliferative Activity of Pt(0)-η^2^-(E)-1,2-Ditosylethene Complexes towards Cancer Cells

With the aim of exploring the potential anticancer effects of platinum(0)-η^2^-1,2-(*E*)ditosylethene complexes, we exposed a set of three distinct human tumor cell lines (ovarian cancer A2780 and its cisplatin-resistant clone A2780*cis*, and triple-negative breast cancer MDA-MB-231) to the synthesized compounds and cisplatin (positive control) for a duration of 96 h. Importantly, the antiproliferative activity was also assessed using MRC-5 normal human lung fibroblast cells in order to establish whether our complexes exhibit in vitro selectivity towards cancer cells.

Before proceeding with biological assays, we initially assessed the stability of the platinum(0)-η^2^-1,2-(*E*)ditosylethene complexes in a 1:1 D_2_O/DMSO-d^6^ solution using ^1^H and ^31^P NMR as standard techniques. Over a 24 h period, negligible changes were observed in the spectra, indicating that the complexes retain their original structure.

However, in the case of complexes **3a**, **4e** and **4g**, a low solubility in DMSO and DMSO/H_2_O solutions was observed. Therefore, these three platinum derivatives were consequently excluded from biological analyses.

The results of the antiproliferative activity assays, presented as half-inhibitory concentrations (IC_50_) values, are summarized in Table 1.

In the A2780 cell line (cisplatin-sensitive ovarian cancer), complexes **2a**, **3b**–**d** and **6a**,**b** exhibited the greatest cytotoxicity, although with higher IC_50_ values compared to cisplatin. Interestingly, in the case of complexes containing isocyanide and phosphine ligands, the best results were obtained with triphenylphosphine and alkyl isocyanides (TIC and CyIC).

In the A2780*cis* cell line (cisplatin-resistant ovarian cancer), the only active compounds are **3b**–**d** and **6a**,**b**. In particular, in the case of bisisocyanide complexes **3b**–**d**, the IC_50_ values are comparable to those obtained on the cisplatin-sensitive cell line, whereas the bisNHC complexes 6a–b present IC_50_ values one order of magnitude lower.

Notably, the cytotoxicity of complexes **3b**–**d** and **6a**,**b** is comparable, or even better, than that of cisplatin.

In the MDA-MB-231 cell line (triple-negative breast cancer), many of the complexes tested exhibited better antiproliferative activity, up to an order of magnitude greater than cisplatin (**2a**, **3b**–**d**, **4a**, **4c**, **4d** and **6b**).

The evaluation of the cytotoxicity of the synthesized complexes on MRC-5 normal cells allows us to establish which complexes present a certain selectivity towards cancer cells. In particular, complexes **3d**, **6a** and **6b** showed a poor cytotoxicity towards normal cells (IC_50_ > 100 µM) and, at the same time, a good cytotoxicity towards ovarian and breast cancer cells, thus suggesting a significant in vitro selectivity.

## 3. Materials and Methods

### 3.1. Solvents and Reagents

All syntheses were conducted in an inert atmosphere (Ar) employing standard Schlenk techniques. Diethyl ether and dichloromethane were dried using molecular sieves (4 Å, 10%) and stored under an argon atmosphere. All other solvents were commercially sourced and utilized without further purification.

2-methyl-6-((phenylthio)methyl)pyridine, Pt(dba)_2_ (*E*)-1,2-ditosylethene and silver-NHC complexes **5a**,**b** were synthetized according to the published procedures [21,22,23,24,25].

Isocyanides and phosphines were employed as supplied.

### 3.2. Instruments

One-dimensional NMR and two-dimensional NMR spectra were acquired using Bruker 300 and 400 MHz Advance spectrometers (Billerica, MA, USA). Chemical shift values (ppm) were referenced to TMS for ^1^H and ^13^C and H_3_PO_4_ for ^31^P. IR spectra were obtained using a PerkinElmer Spectrum One spectrophotometer (Waltham, MA, USA). 

### 3.3. Synthesis of Complex **1**

A total of 0.0448 g (0.2081 mmol) 2-methyl-6-((phenylthio)methyl)pyridine, 0.1353 g (0.200 mmol) of Pt(dba)_2_ and 0.0542 g (0.1611 mmol) of 1,2-(*E*)ditosylethene were suspended in 30 mL of anhydrous dichloromethane under inert atmosphere (Ar) in a 100 mL two-necked flask. The resulting mixture was stirred at room temperature for 6 h and then treated with activated carbon and filtered on a Celite filter. The volume of the resulting clear solution was reduced under vacuum, and finally, diethyl ether was added to promote the precipitation of the final product. The white precipitate was filtered off on a Gooch and dried under vacuum. A total of 0.0866 g (yield 72%) of complex **1** was obtained.

^1^H NMR (CDCl_3_, T = 298 K, ppm, selected peaks) δ: 2.37 (bs, 3H, tol-CH_3_), 2.41 (s, 3H, tol-CH_3_), 3.28 (bs, 3H, Py-CH_3_), 4.13 (bs, 2H, *J*_PtH_ = 69.9 Hz, CH=CH), 4.50–4.69 (bs, 2H, CH_2_N), 6.93 (bd, 2H, tol-H), 7.04 (bd, 2H, tol-H), 7.43 (bd, 4H, tol-H).

^13^C {^1^H} NMR (CDCl_3_, T = 298 K, ppm, selected peaks) δ: 21.7 (CH_3_, tol-CH_3_), 31.9 (CH_3_, Py-CH_3_), 44.5 (CH_2_, CH_2_-S), 49.9 (CH, CH=CH), 120.4 (CH, C^5^-Py), 124.1 (CH, C^3^-Py), 126.8 (CH, tol-H), 129.2 (CH, S-Ph), 129.4 (CH, tol-H), 132.2 (CH, S-Ph), 138.1 (CH, S-Ph), 138.8 (C, *p*-tol-C), 139.6 (C, *i*-tol-C), 142.2 (CH, C^4^Py), 158.2 (C, C^6^Py), 163.0 (C, C^2^Py) 

IR (KBr, cm^−1^) ν_SO_ = 1295 cm^−1^, 1154 cm^−1^; δ_SO_ = 676 cm^−1^.

### 3.4. Synthesis of Pt(0) Complexes Bearing Isocyanide and/or Phosphine Ligands

*[(PPh_3_)_2_Pt(η^2^-(E)-1,2-ditosylethene)]* (**2a**). A total of 0.0868 g (0.1162 mmol) of the precursor **1** was dissolved in ca. 7 mL of anhydrous dichloromethane into a 50 mL two-necked flask under inert atmosphere (Ar). The resulting mixture was treated with 0.0611 g (0.2324 mmol) of PPh_3_, previously dissolved in ca. 3 mL of anhydrous dichloromethane, and stirred at room temperature for 10 min. The addition of a 1:1 mixture of diethylether and *n*-pentane to the concentrated solution yielded the complex **2a** as a white precipitate, which was filtered off on a Gooch and washed with *n*-pentane. A total of 0.0984 g (yield 80%) of complex **2a** was obtained.

^1^H NMR (CDCl_3_, T = 298 K, ppm) δ: 2.34 (s, 6H, tol-CH_3_), 3.61 (m, AA’BB’ system, *J*_PtH_ = 50.7 Hz, 2H, CH=CH), 6.85 (d, *J* = 8.0 Hz, 4H, tol-H), 7.12 (d, *J* = 8.0 Hz, 4H, tol-H), 7.20–7.50 (m, 30H, PPh_3_).

^13^C {^1^H} NMR (CDCl_3_, T = 298 K, ppm, selected peaks) δ: 21.5 (CH_3_, tol-CH_3_), 62.3 (CH, d, *J*_CP_ = 61.7 Hz, *J*_PtC_ = 366 Hz, CH=CH), 126.4 (CH, tol-*m*-CH), 128.9 (CH, tol-*o*-C).

^31^P {^1^H} NMR (CDCl_3_, T = 298 K, ppm) δ: 24.4 (s, *J*_PtP_ = 3720 Hz).

IR (KBr, cm^−1^) ν_SO_ = 1297 cm^−1^, 1141 cm^−1^; δ_SO_ = 674 cm^−1^.

*[(P(p-F-Ph)_3_)_2_Pt(η^2^-(E)-1,2-ditosylethene)]* (**2b**). Compound **2b** was obtained employing 0.0500 g (0.0670 mmol) of complex **1** and 0.0424 g (0.1340 mmol) of P(*p*-F-Ph)_3_. A total of 0.0687 g (yield 88%) of complex **2b** was obtained as a white powder.

^1^H NMR (CDCl_3_, T = 298 K, ppm) δ: 2.36 (s, 6H, tol-CH_3_), 3.56 (m, AA’BB’ system, *J*_PtH_ = 51.0 Hz, 2H, CH=CH), 6.89 (d, *J* = 8.1 Hz, 4H, tol-H), 6.96–7.01 (m, 12H, *o*,*m*-Ph), 7.13 (d, *J* = 8.1 Hz, 4H, tol-H), 7.41–7.49 (m, 12H, *o*,*m*-Ph). 

^13^C {^1^H} NMR (CDCl_3_, T = 298 K, ppm, selected peaks) δ: 21.5 (CH_3_, tol-CH_3_), 62.8 (CH, AA’BB’ system, CH=CH), 126.3 (CH, tol-*m*-CH), 129.2 (CH, tol-*o*-C).

^31^P {^1^H} NMR (CDCl_3_, T = 298 K, ppm) δ: 22.7 (s, *J*_PtP_ = 3741 Hz).

IR (KBr, cm^−1^) ν_SO_ = 1296 cm^−1^, 1161 cm^−1^; δ_SO_ = 671 cm^−1^.

*[(P(2-furyl)_3_)2Pt(η^2^-(E)-1,2-ditosylethene)]* (**2c**). Compound **2c** was obtained employing 0.0500 g (0.0670 mmol) of complex **1** and 0.0354 g (0.1524 mmol) of P(2-furyl)_3_. A total of 0.0496 g (yield 75%) of complex **2c** was obtained as a white powder.

^1^H NMR (CDCl_3_, T = 298 K, ppm) δ: 2.39 (s, 6H, tol-CH_3_), 4.17 (m, AA’BB’ system, *J*_PtH_ = 54.2 Hz, 2H, CH=CH), 6.38–6.40 (m, 6H, 3-furyl-H), 6.95 (d, *J* = 8.0 Hz, 4H, tol-H), 6.98–7.00 (m, 6H, 4-furyl-H), 7.37 (d, *J* = 8.0 Hz, 4H, tol-H), 7.53–7.55 (m, 6H, 5-furyl-H).

^13^C {^1^H} NMR (CDCl_3_, T = 298 K, ppm, selected peaks) δ: 21.6 (CH_3_, tol-CH_3_), 60.7 (CH, AA’BB’ system, *J*_PtC_ = 366 Hz, CH=CH), 110.9 (CH, 4-furyl-CH), 123.1 (m, CH, 3-furyl-CH) 126.7 (CH, tol-*m*-CH), 129.1 (CH, tol-*o*-CH), 142.0 (C, 2-furyl-C), 147.5 (CH, 5-furyl-CH).

^31^P {^1^H} NMR (CDCl_3_, T = 298 K, ppm) δ: −29.9 (s, *J*_PtP_ = 3786 Hz).

IR (KBr, cm^−1^) ν_SO_ = 1299 cm^−1^, 1155 cm^−1^; δ_SO_ = 671 cm^−1^.

*[(DIC)_2_Pt(η^2^-(E)-1,2-ditosylethene)]* (**3a**). Compound **3a** was obtained employing 0.0500 g (0.0670 mmol) of complex **1** and 0.0180 g (0.1360 mmol) of DIC. A total of 0.0491 g (yield 93%) of complex **3a** was obtained as a brownish powder.

^1^H NMR (CDCl_3_, T = 298 K, ppm) δ: 2.32 (s, 6H, tol-CH_3_), 2.47 (s, 12H, DIC-CH_3_), 3.98 (s, 2H, *J*_PtH_ = 55.4 Hz, CH=CH), 7.09 (d, *J* = 8.5 Hz, 4H, tol-H), 7.15 (d, *J* = 7.8 Hz 4H, DIC-*m*-H), 7.27 (t, *J* = 7.8 Hz, 2H, DIC-*p*-H), 7.81 (d, *J* = 8.5 Hz, 4H, tol-H).

^13^C {^1^H} NMR (CDCl_3_, T = 298 K, ppm) δ: 18.9 (CH_3_, DIC-CH_3_), 21.4 (CH_3_, tol-CH_3_), 56.6 (CH, *J*_PtC_ = 326 Hz, CH=CH), 126.8 (C, DIC-*i*-C), 127.1 (CH, DIC-*m*-CH), 128.0 (CH, tol-*m*-CH), 128.0 (CH, tol-*o*-C), 129.2 (CH, DIC-*p*-CH), 135.7 (C, DIC-*o*-C), 139.4 (C, tol-*i*-C), 142.4 (C, tol-*p*-C), 147.1 (C, NC).

IR (KBr, cm^−1^) ν_CN_ = 2179 cm^−1^, 2149 cm^−1^, ν_SO_ = 1306 cm^−1^, 1146 cm^−1^; δ_SO_ = 692 cm^−1^.

*[(TosMIC)_2_Pt(η^2^-(E)-1,2-ditosylethene)]* (**3b**). Compound **3b** was obtained employing 0.0500 g (0.0670 mmol) of complex **1** and 0.0264 g (0.1360 mmol) of TosMIC. A total of 0.0555 g (yield 90%) of complex **3b** was obtained as a white powder.

^1^H NMR (CDCl_3_, T = 298 K, ppm) δ: 2.41 (s, 6H, tol-CH_3_), 2.51 (s, 6H, TosMIC-CH_3_), 3.89 (s, 2H, *J*_PtH_ = 58.6 Hz, CH=CH), 4.93 (s, 4H, *J*_PtH_ = 16.0 Hz, CH_2_S), 7.00 (d, *J* = 8.5 Hz, 4H, tol-H), 7.37 (d, *J* = 8.5 Hz, 4H, tol-H), 7.53 (d, *J* = 8.4 Hz, 4H, TosMIC-H), 8.01 (d, *J* = 8.4 Hz, 4H, TosMIC-H).

^13^C {^1^H} NMR (CDCl_3_, T = 298 K, ppm) δ: 21.6 (CH_3_, tol-CH_3_), 21.9 (CH_3_, TosMIC-CH_3_), 57.6 (CH, *J*_PtC_ = 327 Hz, CH=CH), 63.0 (CH_2_, *J*_PtC_ = 20 Hz, CH_2_S), 126.7 (CH, tol-*m*-CH), 128.3 (CH, tol-*o*-CH), 129.5 (CH, TosMIC-CH), 130.9 (CH, TosMIC-CH), 132.1 (C, TosMIC-C), 138.8 (C, TosMIC-C), 142.6 (C, tol-*i*-C), 146.1 (C, NC), 147.2 (C, tol-*p*-C).

IR (KBr, cm^−1^) ν_CN_ = 2164 cm^−1^, 2202 cm^−1^ ν_SO_ = 1299 cm^−1^, 1155 cm^−1^; δ_SO_ = 674 cm^−1^.

*[(TIC)_2_Pt(η^2^-(E)-1,2-ditosylethene)]* (**3c**). Compound **3c** was obtained employing 0.0485 g (0.0650 mmol) of complex **1** and 0.0108 g (0.1300 mmol) of TIC. A total of 0.0409 g (yield 87%) of complex **3c** was obtained as a white powder.

^1^H NMR (CDCl_3_, T = 298 K, ppm) δ: 1.60 (s, 18H, C(CH_3_)), 2.39 (s, 6H, tol-CH_3_), 3.83 (s, 2H, *J*_PtH_ = 57.2 Hz, CH=CH), 6.97 (d, *J* = 8.2 Hz, 4H, tol-H), 7.43 (d, *J* = 8.2 Hz, 4H, tol-H).

^13^C {^1^H} NMR (CDCl_3_, T = 298 K, ppm) δ: 21.5 (CH_3_, tol-CH_3_), 30.3 (CH_3_, C(CH_3_)), 54.9 (CH, *J*_PtC_ = 337 Hz, CH=CH), 57.2 (C, C(CH_3_)), 126.6 (CH, tol-*m*-CH), 129.1 (CH, tol-*o*-C), 134.4 (C, NC), 139.7 (C, tol-*i*-C), 142.0 (C, tol-*p*-C).

IR (KBr, cm^−1^) ν_CN_ = 2159 cm^−1^, 2196 cm^−1^ ν_SO_ = 1299 cm^−1^, 1158 cm^−1^; δ_SO_ = 675 cm^−1^.

*[(CyIC)_2_Pt(η^2^-(E)-1,2-ditosylethene)]* (**3d**). Compound **3d** was obtained employing 0.0500 g (0.0670 mmol) of complex **1** and 0.0181 g (0.1658 mmol) of CyIC. A total of 0.0444 g (yield 88%) of complex **3d** was obtained as a white powder.

^1^H NMR (CDCl_3_, T = 298 K, ppm) δ: 1.45–1.53 (m, 8H, Cy-CH_2_), 1.77–2.07 (m, 12H, Cy-CH_2_), 2.39 (s, 6H, tol-CH_3_), 3.84 (s, 2H, *J*_PtH_ = 57.3 Hz, CH=CH), 3.91–4.00 (m, 2H, Cy-CH), 6.98 (d, *J* = 8.0 Hz, 4H, tol-H), 7.43 (d, *J* = 8.0 Hz, 4H, tol-H).

^13^C {^1^H} NMR (CDCl_3_, T = 298 K, ppm) δ: 21.6 (CH_3_, tol-CH_3_), 22.8 (CH_2_, Cy-CH_2_), 24.9 (CH_2_, Cy-CH_2_), 32.2 (CH_2_, Cy-CH_2_), 54.6 (CH, Cy-CH), 54.9 (CH, *J*_PtC_ = 338 Hz, CH=CH), 126.7 (CH, tol-*m*-CH), 129.2 (CH, tol-*o*-C), 138.6 (C, NC), 140.0 (C, tol-*i*-C), 142.0 (C, tol-*p*-C).

IR (KBr, cm^−1^) ν_CN_ = 2110 cm^−1^, 2185 cm^−1^ ν_SO_ = 1297 cm^−1^, 1142 cm^−1^; δ_SO_ = 671 cm^−1^.

*[(PPh_3_)(DIC)Pt(η^2^-(E)-1,2-ditosylethene)]* (**4a**). Compound **4a** was obtained employing 0.0500 g (0.0670 mmol) of complex **1**, 0.0088 g (0.0670 mmol) of DIC and 0.0176 g (0.0670 mmol) of PPh_3_. A total of 0.0592 g (yield 95%) of complex **4a** was obtained as a white powder.

^1^H NMR (CDCl_3_, T = 298 K, ppm) δ: 2.14 (s, 6H, DIC-CH_3_), 2.36 (s, 3H, tol-CH_3_), 2.37 (s, 3H, tol-CH_3_), 3.94 (dd, *J*_HH_ = 8.2, *J*_HP_ = 2.2, *J*_PtH_ = 56.0 Hz, 1H, CH=CH *trans*-C), 4.13 (dd, *J*_HH_ = 8.2, *J*_HP_ = 9.5, *J*_PtH_ = 53.0 Hz, 1H, CH=CH *trans*-P), 6.93 (d, *J* = 8.5 Hz, 2H, tol-H), 6.96 (d, *J* = 8.5 Hz, 2H, tol-H), 7.02 (d, *J* = 7.7 Hz, 2H, DIC-*m*-H), 7.16 (t, *J* = 7.7 Hz, 1H, DIC-*p*-H), 7.35 (d, *J* = 8.5 Hz, 2H, tol-H), 7.36–7.45 (m, 11H, tol-H, PPh_3_), 7.63–7.73 (m, 6H, PPh_3_).

^13^C {^1^H} NMR (CDCl_3_, T = 298 K, ppm, selected peaks) δ: 18.5 (CH_3_, DIC-CH_3_), 21.5 (CH_3_, tol-CH_3_), 56.9 (CH, d, *J*_CP_ = 4.8, *J*_PtC_ = 366 Hz, CH=CH *trans*-C), 58.8 (CH, d, *J*_CP_ = 48.7, *J*_PtC_ = 285 Hz, CH=CH *trans*-P), 127.7 (CH, DIC-*m*-CH), 126.6 (CH, tol-*m*-CH), 129.0 (CH, tol-*o*-C), 129.1 (CH, tol-*o*-C), 128.6 (CH, DIC-*p*-CH), 150.6 (C, NC).

^31^P {^1^H} NMR (CDCl_3_, T = 298 K, ppm) δ: 25.4 (s, *J*_PtP_ = 3520 Hz).

IR (KBr, cm^−1^) ν_CN_ = 2123 cm^−1^ ν_SO_ = 1298 cm^−1^, 1141 cm^−1^; δ_SO_ = 674 cm^−1^.

*[(P(p-F-Ph)_3_)(DIC)Pt(η^2^-(E)-1,2-ditosylethene)]* (**4b**). Compound **4b** was obtained employing 0.0500 g (0.0670 mmol) of complex **1**, 0.0088 g (0.0670 mmol) of DIC and 0.0212 g (0.0670 mmol) of P(*p*-F-Ph)_3_. A total of 0.0567 g (yield 86%) of complex **4b** was obtained as a white powder.

^1^H NMR (CDCl_3_, T = 298 K, ppm) δ: 2.18 (s, 6H, DIC-CH_3_), 2.38 (s, 3H, tol-CH_3_), 2.39 (s, 3H, tol-CH_3_), 3.90 (dd, *J*_HH_ = 8.2, *J*_HP_ = 2.4, *J*_PtH_ = 56.1 Hz, 1H, CH=CH *trans*-C), 4.14 (dd, *J*_HH_ = 8.2, *J*_HP_ = 9.5 *J*_PtH_ = 53.0 Hz, 1H, CH=CH *trans*-P), 6.95 (d, *J* = 8.2 Hz, 2H, tol-H), 6.97 (d, *J* = 8.2 Hz, 2H, tol-H), 7.06 (d, *J* = 7.5 Hz, 2H, DIC-*m*-H), 7.11–7.37 (m, 6H, P(C_6_H_4_F)_3_), 7.20 (t, J = 7.5 Hz, 1H, DIC-*p*-H), 7.34 (d, *J* = 8.2 Hz, 2H, tol-H), 7.43 (d, *J* = 8.2 Hz, 2H, tol-H), 7.61–7.71 (m, 6H, P(C_6_H_4_F)_3_).

^13^C {^1^H} NMR (CDCl_3_, T = 298 K, ppm, selected peaks) δ: 18.5 (CH_3_, DIC-CH_3_), 21.6 (CH_3_, tol-CH_3_), 57.2 (CH, d, *J*_CP_ = 4.7, *J*_PtC_ = 336 Hz, CH=CH *trans*-C), 59.5 (CH, d, *J*_CP_ = 48.7, *J*_PtC_ = 286 Hz, CH=CH *trans*-P), 126.5 (CH, tol-*m*-CH), 126.6 (CH, tol-*m*-CH), 129.2 (CH, tol-*o*-C), 127.9 (CH, DIC-*m*-CH), 129.0 (CH, DIC-*p*-CH), 150.2 (C, NC).

^31^P {^1^H} NMR (CDCl_3_, T = 298 K, ppm) δ: 23.4 (s, *J*_PtP_ = 3561 Hz).

IR (KBr, cm^−1^) ν_CN_ = 2127 cm^−1^, 2150 cm^−1^, ν_SO_ = 1298 cm^−1^, 1160 cm^−1^; δ_SO_ = 673 cm^−1^.

*[(PPh_3_)(TosMIC)Pt(η^2^-(E)-1,2-ditosylethene)]* (**4c**). Compound **4c** was obtained employing 0.0503 g (0.0674 mmol) of complex **1**, 0.0131 g (0.0674 mmol) of TosMIC and 0.0177 g (0.0674 mmol) of PPh_3_. A total of 0.0570 g (yield 85%) of complex **4c** was obtained as a white powder. 

^1^H NMR (CDCl_3_, T = 298 K, ppm) δ: 2.37 (s, 3H, TosMIC-CH_3_), 2.41 (s, 6H, tol-CH_3_), 3.81 (dd, *J*_HH_ = 8.3, *J*_HP_ = 2.3, *J*_PtH_ = 56.1 Hz, 1H, CH=CH *trans*-C), 4.02 (dd, *J*_HH_ = 8.3, *J*_HP_ = 9.5, *J*_PtH_ = 53.0 Hz, 1H, CH=CH *trans*-P), 4.93 (broad AB system, 2H, *J*_PtH_ = 16.0 Hz, CH_2_S), 6.92 (d, *J* = 8.0 Hz, 2H, tol-H), 6.98 (d, *J* = 8.0 Hz, 2H, tol-H), 7.30 (d, *J* = 8.0 Hz, 2H, TosMIC-H), 7.35 (d, *J* = 8.0 Hz, 2H, tol-H), 7.40 (d, *J* = 8.0 Hz, 2H, tol-H), 7.44–7.60 (m, 15H, PPh_3_), 7.81 (d, *J* = 8.0 Hz, 2H, TosMIC-H).

^13^C {^1^H} NMR (CDCl_3_, T = 298 K, ppm, selected peaks) δ: 21.6 (CH_3_, tol-CH_3_), 21.8 (CH_3_, TosMIC-CH_3_), 58.0 (CH, d, *J*_CP_ = 4.3, *J*_PtC_ = 330 Hz, CH=CH *trans*-C), 60.7 (CH, d, *J*_CP_ = 47.7, *J*_PtC_ = 283 Hz, CH=CH *trans*-P), 62.8 (CH_2_,CH_2_S), 126.5 (CH, TosMIC-CH), 126.6 (CH, tol-CH), 129.1 (CH, tol-CH), 129.2 (CH, TosMIC-CH), 129.5 (CH, tol-CH), 151.3 (C, NC).

^31^P {^1^H} NMR (CDCl_3_, T = 298 K, ppm) δ: 25.5 (s, *J*_PtP_ = 3487 Hz).

IR (KBr, cm^−1^) ν_CN_ = 2182 cm^−1^ ν_SO_ = 1299 cm^−1^, 1152 cm^−1^; δ_SO_ = 672 cm^−1^.

*[(P(p-F-Ph)_3_)(TosMIC)Pt(η^2^-(E)-1,2-ditosylethene)]* (**4d**). Compound **4d** was obtained employing 0.0500 g (0.0670 mmol) of complex **1**, 0.0131 g (0.0670 mmol) of TosMIC and 0.0212 g (0.0670 mmol) of P(*p*-F-Ph)_3_. A total of 0.0611 g (yield 86%) of complex **4d** was obtained as a white powder.

^1^H NMR (CDCl_3_, T = 298 K, ppm) δ: 2.38 (s, 3H, tol-CH_3_), 2.41 (s, 3H, tol-CH_3_), 2.45 (s, 3H, TosMIC-CH_3_), 3.74 (dd, *J*_HH_ = 8.3, *J*_HP_ = 2.1, *J*_PtH_ = 56.2 Hz, 1H, CH=CH *trans*-C), 4.00 (dd, *J*_HH_ = 8.3, *J*_HP_ = 9.5, *J*_PtH_ = 53.6 Hz, 1H, CH=CH *trans*-P), 4.63 (m, 2H, CH_2_S), 6.94 (d, *J* = 8.0 Hz, 2H, tol-H), 6.98 (d, *J* = 8.0 Hz, 4H, tol-H), 7.15–7.21 (m, 6H, P(C_6_H_4_F)_3_), 7.28 (d, *J* = 8.1 Hz, 2H, TosMIC-H), 7.37 (d, *J* = 8.0 Hz, 4H, tol-H), 7.53–7.61 (m, 6H, P(C_6_H_4_F)_3_), 7.80 (d, *J* = 8.1 Hz, 2H, TosMIC-H).

^13^C {^1^H} NMR (CDCl_3_, T = 298 K, ppm, selected peaks) δ: 21.6 (CH_3_, tol-CH_3_), 21.8 (CH_3_, TosMIC-CH3), 58.3 (CH, d, *J*_CP_ = 5.2, *J*_PtC_ = 334 Hz, CH=CH *trans*-C), 60.1 (CH, d, *J*_CP_ = 47.9, *J*_PtC_ = 283 Hz, CH=CH *trans*-P), 63.0 (CH_2_,CH_2_S), 126.5 (CH, TosMIC-CH), 126.6 (CH, tol-CH), 129.2 (CH, tol-CH), 129.3 (CH, TosMIC-CH), 129.3 (CH, tol-CH), 150.8 (C, NC).

^31^P {^1^H} NMR (CDCl_3_, T = 298 K, ppm) δ: 23.4 (s, *J*_PtP_ = 3541 Hz).

IR (KBr, cm^−1^) ν_CN_ = 2174 cm^−1^ ν_SO_ = 1297 cm^−1^, 1160 cm^−1^; δ_SO_ = 673 cm^−1^.

*[(PPh_3_)(TIC)Pt(η^2^-(E)-1,2-ditosylethene)]* (**4e**). Compound **4e** was obtained employing 0.0500 g (0.0670 mmol) of complex **1**, 0.0056 g (0.0670 mmol) of TIC and 0.0176 g (0.0670 mmol) of PPh_3_. A total of 0.0504 g (yield 86%) of complex **4e** was obtained as a white powder. 

^1^H NMR (CDCl_3_, T = 298 K, ppm) δ: 1.34 (s, 9H, C(CH_3_)), 2.36 (s, 3H, tol-CH_3_), 2.40 (s, 3H, tol-CH_3_), 3.83 (dd, *J*_HH_ = 8.1, *J*_HP_ = 2.0, *J*_PtH_ = 56.9 Hz, 1H, CH=CH *trans*-C), 4.02 (dd, *J*_HH_ = 8.2, *J*_HP_ = 9.4, *J*_PtH_ = 51.8 Hz, 1H, CH=CH *trans*-P), 6.90 (d, *J* = 8.1 Hz, 2H, tol-H), 6.97 (d, *J* = 8.1 Hz, 2H, tol-H), 7.33 (d, *J* = 8.1 Hz, 2H, tol-H), 7.31–7.47 (m, 11H, tol-H, PPh_3_), 7.60–7.68 (m, 6H, PPh_3_).

^13^C {^1^H} NMR (CDCl_3_, T = 298 K, ppm, selected peaks) δ: 21.5 (CH_3_, tol-CH_3_), 30.0 (CH_3_, C(CH_3_)), 56.1 (CH, d, *J*_CP_ = 4.8, *J*_PtC_ = 345 Hz, CH=CH *trans*-C), 57.4 (C, *J*_PtC_ = 12.3 Hz, C(CH_3_)), 58.2 (CH, d, *J*_CP_ = 50.1, *J*_PtC_ = 284 Hz, CH=CH *trans*-P), 126.6 (CH, tol-CH), 129.0 (CH, tol-CH), 129.1 (CH, tol-CH), 134.0 (C, NC).

^31^P {^1^H} NMR (CDCl_3_, T = 298 K, ppm) δ: 26.0 (s, *J*_PtP_ = 3547 Hz).

IR (KBr, cm^−1^) ν_CN_ = 2182 cm^−1^ ν_SO_ = 1297 cm^−1^, 1154 cm^−1^; δ_SO_ = 671 cm^−1^.

*[(P(p-F-Ph)_3_)(TIC)Pt(η^2^-(E)-1,2-ditosylethene)]* (**4f**). Compound **4f** was obtained employing 0.0476 g (0.0638 mmol) of complex **1**, 0.0053 g (0.0638 mmol) of TIC and 0.0202 g (0.0638 mmol) of P(*p*-F-Ph)_3_. A total of 0.0512 g (yield 82%) of complex **4f** was obtained as a white powder.

^1^H NMR (CDCl_3_, T = 298 K, ppm) δ: 1.39 (s, 9H, C(CH_3_)), 2.38 (s, 3H, tol-CH_3_), 2.40 (s, 3H, tol-CH_3_), 3.78 (dd, *J*_HH_ = 8.1, *J*_HP_ = 2.2, *J*_PtH_ = 56.7 Hz, 1H, CH=CH *trans*-C), 4.02 (dd, *J*_HH_ = 8.1, *J*_HP_ = 9.6, *J*_PtH_ = 52.2 Hz, 1H, CH=CH *trans*-P), 6.92 (d, *J* = 8.2 Hz, 2H, tol-H), 6.97 (d, *J* = 8.2 Hz, 2H, tol-H), 7.13–7.19 (m, 6H, P(C_6_H_5_F)_3_), 7.32 (d, *J* = 8.2 Hz, 2H, tol-H), 7.41 (d, *J* = 8.2 Hz, 2H, tol-H), 7.58–7.67 (m, 6H, P(C_6_H_5_F)_3_).

^13^C {^1^H} NMR (CDCl_3_, T = 298 K, ppm, selected peaks) δ: 21.6 (CH_3_, tol-CH_3_), 30.0 (CH_3_, C(CH_3_)), 56.5 (CH, d, *J*_CP_ = 4.6, *J*_PtC_ = 346 Hz, CH=CH *trans*-C), 57.7 (C, *J*_PtC_ = 12.3 Hz, C(CH_3_)), 58.7 (CH, d, *J*_CP_ = 50.4, J_PtC_ = 282 Hz, CH=CH *trans*-P), 126.5 (CH, tol-CH), 129.1 (CH, tol-CH), 129.2 (CH, tol-CH), 139.8 (C, NC).

^31^P {^1^H} NMR (CDCl_3_, T = 298 K, ppm) δ: 24.1 (s, *J*_PtP_ = 3596 Hz).

IR (KBr, cm^−1^) ν_CN_ = 2175 cm^−1^ ν_SO_ = 1297 cm^−1^, 1160 cm^−1^; δ_SO_ = 672 cm^−1^.

*[(PPh_3_)(CyIC)Pt(η^2^-(E)-1,2-ditosylethene)]* (**4g**). Compound **4g** was obtained employing 0.0500 g (0.0670 mmol) of complex **1**, 0.0073 g (0.0670 mmol) of CyIC and 0.0176 g (0.0670 mmol) of PPh_3_. A total of 0.0524 g (yield 87%) of complex **4g** was obtained as a white powder. 

^1^H NMR (CDCl_3_, T = 298 K, ppm) δ: 1.23–1.54 (m, 6H, Cy-CH_2_), 1.59–1.83 (m, 4H, Cy-CH_2_), 2.36 (s, 3H, tol-CH_3_), 2.40 (s, 3H, tol-CH_3_), 3.57–3.66 (m, 1H, Cy-CH), 3.76 (dd, *J*_HH_ = 8.1, *J*_HP_ = 2.1, *J*_PtH_ = 56.7 Hz, 1H, CH=CH *trans*-C), 3.98 (dd, *J*_HH_ = 8.1, *J*_HP_ = 9.4 *J*_PtH_ = 51.9 Hz, 1H, CH=CH *trans*-P), 6.90 (d, *J* = 8.0 Hz, 2H, tol-H), 6.97 (d, *J* = 8.0 Hz, 2H, tol-H), 7.32 (d, *J* = 8.0 Hz, 2H, tol-H), 7.31–7.45 (m, 11H, tol-H, PPh_3_), 7.60–7.69 (m, 6H, PPh_3_).

^13^C {^1^H} NMR (CDCl_3_, T = 298 K, ppm, selected peaks) δ: 21.6 (CH_3_, tol-CH_3_), 22.9 (CH_2_, Cy-CH_2_), 24.8 (CH_2_, Cy-CH_2_), 32.0 (CH_2_, Cy-CH_2_), 54.4 (CH, Cy-CH), 56.1 (CH, d, *J*_CP_ = 4.8, *J*_PtC_ = 344 Hz, CH=CH *trans*-C), 58.2 (CH, d, *J*_CP_ = 50.2, *J*_PtC_ = 284 Hz, CH=CH *trans*-P), 126.6 (CH, tol-CH), 129.0 (CH, tol-CH), 129.1 (CH, tol-CH), 133.9 (C, NC).

^31^P {^1^H} NMR (CDCl_3_, T = 298 K, ppm) d: 26.0 (s, *J*_PtP_ = 3547 Hz).

IR (KBr, cm^−1^) ν_CN_ = 2190 cm^−1^ ν_SO_ = 1296 cm^−1^, 1153 cm^−1^; δ_SO_ = 671 cm^−1^.

*[(P(p-F-Ph)_3_)(CyIC)Pt(η^2^-(E)-1,2-ditosylethene)]* (**4h**). Compound **4h** was obtained employing 0.0616 g (0.0824 mmol) of complex **1**, 0.0090 g (0.0824 mmol) of CyIC and 0.0261 g (0.0824 mmol) of P(*p*-F-Ph)_3_. A total of 0.0695 g (yield 88%) of complex **4h** was obtained as a white powder.

^1^H NMR (CDCl_3_, T = 298 K, ppm) δ: 1.28–1.54 (m, 6H, Cy-CH_2_), 1.59–1.85 (m, 4H, Cy-CH_2_), 2.37 (s, 3H, tol-CH_3_), 2.40 (s, 3H, tol-CH_3_), 3.65–3.73 (m, 1H, Cy-CH), 3.78 (dd, *J*_HH_ = 8.2, *J*_HP_ = 2.2, *J*_PtH_ = 56.9 Hz, 1H, CH=CH *trans*-C), 3.98 (dd, *J*_HH_ = 8.2, *J*_HP_ = 9.6 *J*_PtH_ = 52.2 Hz, 1H, CH=CH *trans*-P), 6.92 (d, *J* = 8.1 Hz, 2H, tol-H), 6.97 (d, *J* = 8.1 Hz, 2H, tol-H), 7.13–7.19 (m, 6H, P(C_6_H_4_F)_3_), 7.32 (d, *J* = 8.1 Hz, 2H, tol-H), 7.41 (d, *J* = 8.1 Hz, 2H, tol-H), 7.58–7.67 (m, 6H, P(C_6_H_4_F)_3_).

^13^C {^1^H} NMR (CDCl_3_, T = 298 K, ppm, selected peaks) δ: 21.6 (CH_3_, tol-CH_3_), 22.8 (CH_2_, Cy-CH_2_), 24.8 (CH_2_, Cy-CH_2_), 32.0 (CH_2_, Cy-CH_2_), 54.5 (CH, Cy-CH), 56.4 (CH, d, *J*_CP_ = 5.2, *J*_PtC_ = 344 Hz, CH=CH *trans*-C), 58.6 (CH, d, *J*_CP_ = 50.6, *J*_PtC_ = 284 Hz, CH=CH *trans*-P), 126.5 (CH, tol-CH), 129.1 (CH, tol-CH), 129.2 (CH, tol-CH), 139.8 (C, NC).

^31^P {^1^H} NMR (CDCl_3_, T = 298 K, ppm) δ: 24.1 (s, *J*_PtP_ = 3589 Hz).

IR (KBr, cm^−1^) ν_CN_ = 2179 cm^−1^ ν_SO_ = 1297 cm^−1^, 1161 cm^−1^; δ_SO_ = 672 cm^−1^.

### 3.5. Synthesis of Pt(0) Complexes Bearing N-Heterocyclic Carbene (NHC) Ligands

*[(TolCH_2_ImCH_2_Tol)_2_Pt(η^2^-(E)-1,2-ditosylethene)]* (**6a**). A total of 0.0500 g (0.0670 mmol) of the precursor **1** was dissolved in ca. 7 mL of anhydrous dichloromethane into a 50 mL two-necked flask under inert atmosphere (Ar). The resulting mixture was treated with 0.0623 g (0.1340 mmol) of silver–NHC complex **5a**, previously dissolved in ca. 7 mL of anhydrous dichloromethane, and stirred at room temperature for 4 days. Afterwards, the mixture was treated with activated carbon and filtered on a Celite filter. The addition of a 1:1 mixture of diethylether and *n*-pentane to the concentrated solution yielded the complex **6a** as a white precipitate, which was filtered off on a Gooch and washed with *n*-pentane. This gave 0.0591 g (yield 81%) of complex **6a**.

^1^H NMR (CDCl_3_, T = 298 K, ppm) δ: 2.32 (s, 12H, tol-CH_3_ (NHC)), 2.35 (s, 6H, tol-CH_3_), 3.40 (s, 2H, *J*_PtH_ = 47.3 Hz, CH=CH), 5.04 (d, *J* = 14.8 Hz, 4H,CH_2_-tol (NHC)), 5.46 (d, *J* = 14.8 Hz, 4H, CH_2_-tol (NHC)), 6.63 (s, *J*_PtH_ = 11.0 Hz, 4H, CH=CH (NHC)), 6.90 (d, *J* = 8.0 Hz, 4H, tol-H), 7.11, 7.09 (AB system, *J* = 8.7 Hz, 16H, tol-H (NHC)), 7.37 (d, *J* = 8.0 Hz, 4H, tol-H). 

^13^C{^1^H}NMR (CDCl_3_, T = 298 K, ppm) δ: 21.1 (CH_3_, tol-CH_3_ (NHC)) 21.5 (CH_3_, tol-CH_3_), 48.6 (CH, *J*_PtC_ = 297 Hz, CH=CH), 53.8 (CH_2_, *J*_PtC_ = 39.0 Hz, N-CH_2_), 120.1 (CH, *J*_PtC_ = 35.8 Hz, CH=CH (NHC)), 126.2 (CH, tol-*m*-CH), 128.2 (CH, tol-*m*-CH (NHC)), 128.8 (CH, tol-*o*-CH), 129.3 (CH, tol-*o*-CH (NHC)), 133.7 (C, tol-*i*-C (NHC)), 137.3 (C, tol-*p*-C (NHC)), 140.6 (C, tol-*i*-C), 141.6 (C, tol-*p*-C), 178.6 (C, *J*_PtC_ = 1374 Hz, NCN).

IR (KBr, cm^−1^) ν_SO_ = 1279 cm^−1^, 1129 cm^−1^; δ_SO_ = 667 cm^−1^.

*[(CH_3_ImCH_2_Ph)_2_Pt(η^2^-(E)-1,2-ditosylethene)]* (**6b**). A total of 0.0500 g (0.0670 mmol) of the precursor **1** was dissolved in ca. 7 mL of anhydrous dichloromethane into a 50 mL two-necked flask under inert atmosphere (Ar). The resulting mixture was treated with 0.0482 g (0.1340 mmol) of silver–NHC complex **5b**, previously dissolved in ca. 7 mL of anhydrous dichloromethane, and stirred at room temperature for 3 days. Afterwards, the mixture was treated with activated carbon and filtered on a Celite filter. The addition of a 1:1 mixture of diethylether and *n*-pentane to the concentrated solution yielded the complex **6b** as a white precipitate, which was filtered off on a Gooch and washed with *n*-pentane. This gave 0.0587 g (yield 90%) of complex **6b**.

^1^H NMR (CDCl_3_, T = 298 K, ppm) δ: 2.36 (s, 6H, tol-CH_3_), 3.42 (s, 2H, *J*_PtH_ = 47.6 Hz, CH=CH), 3.76 (s, 6H, *J*_PtH_ = 5.3 Hz N-CH_3_ (NHC)), 5.39–5.46 (AB system, *J* = 15.3 Hz, 4H, CH_2_-tol (NHC)), 6.70 (d, *J* = 2.0 Hz, *J*_PtH_ = 13.3 Hz, CH=CH (NHC)), 6.79 (d, *J* = 2.0 Hz, *J*_PtH_ = 13.3 Hz, 4H, CH=CH (NHC)), 6.90 (d, *J* = 8.3 Hz, 4H, tol-H), 7.07–7.10, (m, 4H, Ph-H (NHC)), 7.25–7.30, (m, 6H, Ph-H (NHC)), 7.37 (d, *J* = 8.3 Hz, 4H, tol-H). 

^13^C {^1^H} NMR (CDCl_3_, T = 298 K, ppm) δ: 21.5 (CH_3_, tol-CH_3_), 37.9 (CH_3_, *J*_PtC_ = 39.9 Hz, N-CH_3_), 47.8 (CH, *J*_PtC_ = 296 Hz, CH=CH), 53.8 (CH_2_, *J*_PtC_ = 39.5 Hz, N-CH_2_), 119.8 (CH, *J*_PtC_ = 31.3 Hz, CH=CH (NHC)), 121.8 (CH, *J*_PtC_ = 31.3 Hz, CH=CH (NHC)), 126.2 (CH, tol-*m*-CH), 127.5 (CH, tol-*m*-CH (NHC)), 128.6 (CH, tol-*o*-CH), 128.8 (CH, tol-*o*-CH (NHC)), 136.8 (C, tol-*i*-C (NHC)), 140.7 (C, tol-*p*-C (NHC)), 141.5 (C, tol-*i*-C), 141.5 (C, tol-*p*-C), 178.1 (C, *J*_PtC_ = 1365 Hz NCN).

IR (KBr, cm^−1^) ν_SO_ = 1277 cm^−1^, 1130 cm^−1^; δ_SO_ = 668 cm^−1^.

### 3.6. Computational Details

All calculations were performed by using DFT, as implemented in the ORCA 4.2 suite of ab initio quantum chemistry programs [26]. Geometry optimizations were performed with the B97M-D3BJ functional [27] using the double-ζ-quality def2-SVP [28] basis set that included relativistic core potentials for Pd.

Solvent effects (dichloromethane, ε = 8.93) were included using CPCM. More accurate single-point energies were computed from the optimized geometries using ωB97M-V [29] DFT and the triple-ζ-quality def2-TZVPP [28] basis set. Vibrational frequencies were computed at the B97M-D3BJ/def2-SVP level of theory to derive the Gibbs free energy.

### 3.7. Cytotoxicity Assay

Two types of ovarian cancer cell lines (A2780 and A2780*cis*), one breast cancer cell line (MDA-MB-231) and a normal cell line (MRC-5) were cultured following the supplier’s guidelines (Sigma-Aldrich, St. Louis, MO, USA) and maintained at 37 °C in a humidified atmosphere containing 5% CO_2_. In 96-well plates, 1∙10^3^ cancer cells and 8∙10^3^ MRC-5 cells were seeded and treated after 24 h with six different concentrations of Pt(0) complexes (0.001, 0.01, 0.1, 1, 10, 100 µM) [30,31]. Stock solutions (10 mM) of all platinum complexes were prepared using DMSO as a solvent. After 96 h of treatment, cell viability was assessed using a CellTiter-Glo assay (Promega, Madison, WI, USA) with Tecan M1000 or SynergyH1 microplate readers (Mennedorf, Switzerland). IC_50_ values were determined from logistical dose–response curves using GraphPad Prism 8 software. Triplicate measurements were taken to calculate averages, and standard deviations were represented by error bars.

### 3.8. Crystal Structure Determination

The data of the **2a**, **3a**, **3b**, **4b** and **6b** crystals were collected at the XRD2 beamline of the Elettra Synchrotron, Trieste (Italy) [32], using a monochromatic wavelength of 0.620 Å, at 100 K or 298 K. The datasets were integrated, scaled and corrected for Lorentz, absorption and polarization effects using the XDS package [33]. Data from two random orientations have been merged to obtain complete datasets for the triclinic **3a**, **3b**, **4b** and **6b** crystal forms, using CCP4-Aimless [34,35] code. The structures were solved by direct methods using the SHELXT program [36] and refined using full-matrix least squares implemented in SHELXL−2019/3 [37]. Thermal motions for all non-hydrogen atoms have been treated anisotropically, and hydrogens have been included at calculated positions, riding on their carrier atoms. Thermal and geometric restrains (SIMU, DFIX, DANG, FLAT) have been used to properly model disordered and poorly defined fragments. Data for **4b** have been refined as a racemic twin (twin fraction refined to 30%). The Coot program was used for structure building [38,39]. Pictures were prepared using Ortep3 [40] and Pymol [41] software. The crystal data are given in Table S1.

Crystallographic data have been deposited at the Cambridge Crystallographic Data Centre and allocated the deposition number CCDC 2323081, 2323083, 2323084, 2323085, 2323087 and 2323082 for **3a** at 100 K, **3b** at 100 K, **2a** at 100 K, **2a** at 298 K, **4b** at 100 K and **6b** at 100 K, respectively. These data can be obtained free of charge via https://www.ccdc.cam.ac.uk/structures (accessed on 9 February 2024).

## 4. Conclusions

In conclusion, we have reported a two-step synthesis of 17 new platinum(0) complexes bearing different ancillary ligands and (*E*)-1,2-ditosylethene as a model olefin. These compounds were exhaustively characterized by NMR and IR analyses, highlighting the characteristic signals of the investigated complexes. Moreover, for some of them, it was possible to confirm the structure by XRD analysis. The most stable compounds were tested towards two different ovarian cancer cell lines (A2780 and A2780*cis*) and one breast cancer cell line (MDA-MB-231). Most of the compounds exhibited good cytotoxicity (in the micromolar range) towards A2780 and MDA-MB-231 cells, with IC_50_ values comparable and sometimes even better than cisplatin. On the contrary, only a few compounds showed cytotoxicity towards cisplatin-resistant cancer cells (A2780*cis*). Moreover, in the case of complexes containing isocyanide and phosphine ligands, the best results were obtained with triphenylphosphine and alkyl isocyanides (TIC and CyIC).

Notably, complexes **3d**, **6a** and **6b** showed a poor cytotoxicity towards normal cells (IC_50_ > 100 µM) and, at the same time, a good antiproliferative activity towards cancer cells.

We strongly believe that these three last complexes deserve to be investigated in depth in the future, with the aim of defining in detail their mechanism of action and cytotoxicity on more complex biological systems such as organoids and animal models.

## Figures and Tables

**Table 1:** Antiproliferative activity on A2780, A2780*cis*, MDA-MB-231 and MRC-5 cell lines.

Compound	IC_50_ (µM)			
	A2780	A2780*cis*	MDA-MB-231	MRC-5
Cisplatin	0.19 ± 0.08	5.4 ± 0.1	6.4 ± 0.5	2.9 ± 0.1
**2a**	5.5 ± 0.6	>100	2.6 ± 0.4	>100
**2b**	>100	>100	36 ± 6	>100
**2c**	17.2 ± 0.8	>100	>100	>100
**3b**	5.4 ± 0.7	11 ± 6	5.52 ± 0.03	22 ± 6
**3c**	2.6 ± 0.3	3.1 ± 0.9	3.4 ± 0.4	3.5 ± 0.1
**3d**	1.18 ± 0.09	1.4 ± 0.2	1.2 ± 0.3	9 ± 1
**4a**	35 ± 3	>100	1.8 ± 0.6	59 ± 8
**4b**	85 ± 23	>100	30 ± 13	>100
**4c**	55 ± 17	>100	1.0 ± 0.8	15 ± 3
**4d**	>100	>100	0.4 ± 0.1	14 ± 1
**4f**	>100	>100	21 ± 2	>100
**4h**	41 ± 10	>100	37 ± 13	>100
**6a**	1.6 ± 0.1	10 ± 3	36 ± 11	>100
**6b**	1.9 ± 0.3	12.91 ± 0.06	5.4 ± 0.9	61 ± 13

Data after 96 h of incubation. Stock solutions in DMSO for all complexes; stock solutions in H_2_O/NaCl for cisplatin. A2780 (cisplatin-sensitive ovarian cancer cells), A2780*cis* (cisplatin-resistant ovarian cancer cells), MDA-MB-231 (triple-negative breast cancer cells), MRC-5 (normal lung fibroblasts).

## Data Availability

Data are contained within the article and Supplementary Materials.

## Supplementary Materials

The following supporting information can be downloaded at: https://www.mdpi.com/article/10.3390/molecules29051119/s1, Figure S1: ^1^H NMR spectrum of compound **1** in CDCl_3_ at 298 K; Figure S2: ^13^C{^1^H} NMR spectrum of compound **1** in CDCl_3_ at 298 K; Figure S3: ^1^H NMR spectrum of compound **2a** in CDCl_3_ at 298 K; Figure S4: ^13^C{^1^H} NMR spectrum of compound **2a** in CDCl_3_ at 298 K; Figure S5: ^31^P{^1^H} NMR spectrum of compound **2a** in CDCl_3_ at 298 K; Figure S6: ^1^H NMR spectrum of compound **2b** in CDCl_3_ at 298 K; Figure S7: ^13^C{^1^H} NMR spectrum of compound **2b** in CDCl_3_ at 298 K; Figure S8: ^31^P{^1^H} NMR spectrum of compound **2b** in CDCl_3_ at 298 K; Figure S9: ^1^H NMR spectrum of compound **2c** in CDCl_3_ at 298 K; Figure S10: ^13^C{^1^H} NMR spectrum of compound **2c** in CDCl_3_ at 298 K; Figure S11: ^31^P{^1^H} NMR spectrum of compound **2c** in CDCl_3_ at 298 K; Figure S12: ^1^H NMR spectrum of compound **3a** in CDCl_3_ at 298 K; Figure S13: ^13^C{^1^H} NMR spectrum of compound **3a** in CDCl_3_ at 298 K; Figure S14: ^1^H NMR spectrum of compound **3b** in CDCl_3_ at 298 K; Figure S15: ^13^C{^1^H} NMR spectrum of compound **3b** in CDCl_3_ at 298 K; Figure S16: ^1^H NMR spectrum of compound **3c** in CDCl_3_ at 298 K; Figure S17: ^13^C{^1^H} NMR spectrum of compound **3c** in CDCl_3_ at 298 K; Figure S18: ^1^H NMR spectrum of compound **3d** in CDCl_3_ at 298 K; Figure S19: ^13^C{^1^H} NMR spectrum of compound **3d** in CDCl_3_ at 298 K; Figure S20: ^1^H NMR spectrum of compound **4a** in CDCl_3_ at 298 K; Figure S21: ^13^C{^1^H} NMR spectrum of compound **4a** in CDCl_3_ at 298 K; Figure S22: ^31^P{^1^H} NMR spectrum of compound **4a** in CDCl_3_ at 298 K; Figure S23: ^1^H NMR spectrum of compound **4b** in CDCl_3_ at 298 K; Figure S24: ^13^C{^1^H} NMR spectrum of compound **4b** in CDCl_3_ at 298 K; Figure S25: ^31^P{^1^H} NMR spectrum of compound **4b** in CDCl_3_ at 298 K; Figure S26: ^1^H NMR spectrum of compound **4c** in CDCl_3_ at 298 K; Figure S27: ^13^C{^1^H} NMR spectrum of compound **4c** in CDCl_3_ at 298 K; Figure S28: ^31^P{^1^H} NMR spectrum of compound **4c** in CDCl_3_ at 298K; Figure S29: ^1^H NMR spectrum of compound **4d** in CDCl_3_ at 298 K; Figure S30: ^13^C{^1^H} NMR spectrum of compound **4d** in CDCl_3_ at 298 K; Figure S31: ^31^P{^1^H} NMR spectrum of compound **4d** in CDCl_3_ at 298 K; Figure S32: ^1^H NMR spectrum of compound **4e** in CDCl_3_ at 298 K; Figure S33: ^13^C{^1^H} NMR spectrum of compound **4e** in CDCl_3_ at 298 K; Figure S34: ^31^P{^1^H} NMR spectrum of compound **4e** in CDCl_3_ at 298 K; Figure S35: ^1^H NMR spectrum of compound **4f** in CDCl_3_ at 298 K; Figure S36: ^13^C{^1^H} NMR spectrum of compound **4f** in CDCl_3_ at 298 K; Figure S37: ^31^P{^1^H} NMR spectrum of compound **4f** in CDCl_3_ at 298 K; Figure S38: ^1^H NMR spectrum of compound **4g** in CDCl_3_ at 298 K; Figure S39: ^13^C{^1^H} NMR spectrum of compound **4g** in CDCl_3_ at 298 K; Figure S40: ^31^P{^1^H} NMR spectrum of compound **4g** in CDCl_3_ at 298 K; Figure S41: ^1^H NMR spectrum of compound **4h** in CDCl_3_ at 298 K; Figure S42: ^13^C{^1^H} NMR spectrum of compound **4h** in CDCl_3_ at 298 K; Figure S43: ^13^C{^1^H} NMR spectrum of compound **4h** in CDCl_3_ at 298 K; Figure S44: ^1^H NMR spectrum of compound **6a** in CDCl_3_ at 298 K; Figure S45: ^13^C{^1^H} NMR spectrum of compound **6a** in CDCl_3_ at 298 K; Figure S46: ^1^H NMR spectrum of compound **6b** in CDCl_3_ at 298 K; Figure S47: ^13^C{^1^H} NMR spectrum of compound **6b** in CDCl_3_ at 298 K; Figure S48: IR spectrum of compound **1**; Figure S49: IR spectrum of compound **2a**; Figure S50: IR spectrum of compound **3a**; Figure S51: IR spectrum of compound **4a**; Table S1: Crystallographic data; Table S2: Selected platinum distances and angles for **3a** at 100 K; Table S3: Selected platinum distances and angles for **3b** at 100 K; Table S4: Selected platinum distances and angles for **2a** at 100 K; Table S5: Selected platinum distances and angles for **2a** at 298 K; Table S6: Selected platinum distances and angles for **4b** at 100 K; Table S7: Selected platinum distances and angles for **6b** at 100 K.
