# Supplementary material for: Platinum(0)-η2-1,2-(E)ditosylethene Complexes Bearing Phosphine, Isocyanide and N-Heterocyclic Carbene Ligands: Synthesis and Cytotoxicity towards Ovarian and Breast Cancer Cells
Source: Molecules. 2024 Mar 1;29(5):1119. doi: 10.3390/molecules29051119 (PMC10934098; doi:10.3390/molecules29051119)

checkCIF/PLATON report

Structure factors have been supplied for datablock(s) NC5LT

THIS REPORT IS FOR GUIDANCE ONLY. IF USED AS PART OF A REVIEW PROCEDURE FOR PUBLICATION, IT SHOULD NOT REPLACE THE EXPERTISE OF AN EXPERIENCED CRYSTALLOGRAPHIC REFEREE.

No syntax errors found. CIF dictionary Interpreting this report

Datablock: NC5LT

Bond precision: C-C = 0.0021 A Wavelength=0.62000

Cell:	a=14.660(3)	b=17.981(4)	c=18.585(4)
	alpha=101.57(3)	beta=96.12(3)	gamma=98.80(3)
Temperature:	100 K		

	Calculated	Reported
Volume	4695.1(19)	4695.1(18)
Space group	P -1	P -1
Hall group	-P 1	-P 1
Moiety formula	C52 H46 O4 P2 Pt S2, 0.3(C4 H10 O), 0.175(C H2 Cl2)	C52 H46 O4 P2 Pt S2, 0.25 (C4 H10 O), 0.25 (C H2 Cl2)
Sum formula	C53.37 H49.35 Cl0.35 O4.30 P2 Pt S2	C53.25 H48.75 Cl0.50 O4.25 P2 Pt S2
Mr	1093.08	1095.54
Dx,g cm-3	1.546	1.550
Z	4	4
Mu (mm-1)	2.245	2.251
F000	2199.7	2203.0
F000'	2198.31	
h,k,lmax	24,29,30	23,29,30
Nref	45624	41421
Tmin,Tmax	0.874,0.956	
Tmin'	0.798	

Correction method= Not given

Data completeness= 0.908 Theta(max)= 31.129

R(reflections)= 0.0295(39404) wR2(reflections)= 0.0737(41421)

S = 1.050 Npar= 1193

The following ALERTS were generated. Each ALERT has the format
test-name_ALERT_alert-type_alert-level.
Click on the hyperlinks for more details of the test.

Alert level C

```
SHFSU01_ALERT_2_C  The absolute value of parameter shift to su ratio > 0.05
            Absolute value of the parameter shift to su ratio given   0.056
            Additional refinement cycles may be required.
PLAT041_ALERT_1_C Calc. and Reported SumFormula    Strings  Differ     Please Check
PLAT042_ALERT_1_C Calc. and Reported MoietyFormula Strings  Differ     Please Check
PLAT043_ALERT_1_C Calculated and Reported Mol. Weight Differ by ..       2.46 Check
PLAT068_ALERT_1_C Reported F000 Differs from Calcd (or Missing)...     Please Check
PLAT077_ALERT_4_C Unitcell Contains Non-integer Number of Atoms ..     Please Check
PLAT080_ALERT_2_C Maximum Shift/Error ...........................       0.06 Why ?
PLAT213_ALERT_2_C Atom C4_13           has ADP max/min Ratio .....        3.4 prolat
PLAT220_ALERT_2_C NonSolvent   Resd 1  C   Ueq(max)/Ueq(min) Range        3.5 Ratio
PLAT220_ALERT_2_C NonSolvent   Resd 2  C   Ueq(max)/Ueq(min) Range        4.2 Ratio
PLAT911_ALERT_3_C Missing FCF Refl Between Thmin & STh/L=    0.600         11 Report
PLAT971_ALERT_2_C Check Calcd Resid. Dens.  0.65Ang From Pt_11          2.07 eA-3
PLAT971_ALERT_2_C Check Calcd Resid. Dens.  0.60Ang From Pt_21          1.89 eA-3
PLAT971_ALERT_2_C Check Calcd Resid. Dens.  1.04Ang From C1B_92         1.84 eA-3
PLAT971_ALERT_2_C Check Calcd Resid. Dens.  0.53Ang From Pt_21          1.83 eA-3
PLAT971_ALERT_2_C Check Calcd Resid. Dens.  0.55Ang From Pt_11          1.78 eA-3
PLAT971_ALERT_2_C Check Calcd Resid. Dens.  0.93Ang From Pt_21          1.71 eA-3
PLAT972_ALERT_2_C Check Calcd Resid. Dens.  0.58Ang From Pt_21         -2.14 eA-3
PLAT972_ALERT_2_C Check Calcd Resid. Dens.  0.68Ang From Pt_21         -1.62 eA-3
PLAT972_ALERT_2_C Check Calcd Resid. Dens.  0.67Ang From Pt_11         -1.60 eA-3
PLAT972_ALERT_2_C Check Calcd Resid. Dens.  0.50Ang From Pt_11         -1.53 eA-3
PLAT972_ALERT_2_C Check Calcd Resid. Dens.  0.86Ang From Pt_11         -1.52 eA-3
PLAT972_ALERT_2_C Check Calcd Resid. Dens.  0.60Ang From Pt_11         -1.51 eA-3
```

Alert level G

```
FORMU01_ALERT_1_G  There is a discrepancy between the atom counts in the
            _chemical_formula_sum and _chemical_formula_moiety. This is
            usually due to the moiety formula being in the wrong format.
            Atom count from _chemical_formula_sum:   C53.25 H48.75 Cl0.5 O4.25 P2
            Atom count from _chemical_formula_moiety:C53.25 H49 Cl0.5 O4.25 P2 Pt1
FORMU01_ALERT_2_G  There is a discrepancy between the atom counts in the
            _chemical_formula_sum and the formula from the _atom_site* data.
            Atom count from _chemical_formula_sum:C53.25 H48.75 Cl0.5 O4.25 P2 Pt1
            Atom count from the _atom_site data:  C53.37450 H49.34899 Cl0.349 O4.3
ABSMU01_ALERT_1_G  Calculation of _exptl_absorpt_correction_mu
                not performed for this radiation type.
CELLZ01_ALERT_1_G Difference between formula and atom_site contents detected.
CELLZ01_ALERT_1_G ALERT: Large difference may be due to a
            symmetry error - see SYMMG tests
           From the CIF: _cell_formula_units_Z    4
           From the CIF: _chemical_formula_sum  C53.25 H48.75 Cl0.50 O4.25 P2 Pt S
           TEST: Compare cell contents of formula and atom_site data

           atom    Z*formula  cif sites diff
           C        213.00    213.50   -0.50
           H        195.00    197.40   -2.40
           Cl         2.00      1.40    0.60
```

```
          O         17.00     17.20   -0.20
          P          8.00      8.00    0.00
          Pt         4.00      4.00    0.00
          S          8.00      8.00    0.00
PLAT002_ALERT_2_G Number of Distance or Angle Restraints on AtSite         11 Note
PLAT003_ALERT_2_G Number of Uiso or Uij Restrained non-H Atoms ...         11 Report
PLAT083_ALERT_2_G SHELXL Second Parameter in WGHT  Unusually Large       6.10 Why ?
PLAT092_ALERT_4_G Check: Wavelength Given is not Cu,Ga,Mo,Ag,In Ka    0.62000 Ang.
PLAT154_ALERT_1_G The s.u.'s on the Cell Angles are Equal ..(Note)       0.03 Degree
PLAT172_ALERT_4_G The CIF-Embedded .res File Contains DFIX Records          3 Report
PLAT173_ALERT_4_G The CIF-Embedded .res File Contains DANG Records          3 Report
PLAT175_ALERT_4_G The CIF-Embedded .res File Contains SAME Records          1 Report
PLAT176_ALERT_4_G The CIF-Embedded .res File Contains SADI Records          2 Report
PLAT178_ALERT_4_G The CIF-Embedded .res File Contains SIMU Records          1 Report
PLAT188_ALERT_3_G A Non-default SIMU Restraint Value has been used     0.0100 Report
PLAT232_ALERT_2_G Hirshfeld Test Diff (M-X)  Pt_21    --Pa_24    .       12.6 s.u.
PLAT232_ALERT_2_G Hirshfeld Test Diff (M-X)  Pt_11    --P_13     .        6.4 s.u.
PLAT301_ALERT_3_G Main Residue  Disorder .............(Resd  1  )        31% Note
PLAT301_ALERT_3_G Main Residue  Disorder .............(Resd  2  )        10% Note
PLAT302_ALERT_4_G Anion/Solvent/Minor-Residue Disorder (Resd  3  )       100% Note
PLAT302_ALERT_4_G Anion/Solvent/Minor-Residue Disorder (Resd  4  )       100% Note
PLAT302_ALERT_4_G Anion/Solvent/Minor-Residue Disorder (Resd  5  )       100% Note
PLAT304_ALERT_4_G Non-Integer Number of Atoms in ..... (Resd  4  )       1.03 Check
PLAT304_ALERT_4_G Non-Integer Number of Atoms in ..... (Resd  5  )       0.72 Check
PLAT413_ALERT_2_G Short Inter XH3 .. XHn     H1A_91   ..H4_14    .       2.10 Ang.
                                               2-x,1-y,1-z  =      2_766 Check
PLAT413_ALERT_2_G Short Inter XH3 .. XHn     H4A3_91  ..H4_22    .       2.04 Ang.
                                                     x,y,z  =      1_555 Check
PLAT432_ALERT_2_G Short Inter X...Y Contact  C5_12    ..C1B_92   .       3.06 Ang.
                                               1-x,1-y,1-z  =      2_666 Check
PLAT432_ALERT_2_G Short Inter X...Y Contact  C4_12    ..C1B_92   .       3.16 Ang.
                                               1-x,1-y,1-z  =      2_666 Check
PLAT720_ALERT_4_G Number of Unusual/Non-Standard Labels ..........        284 Note
PLAT789_ALERT_4_G Atoms with Negative _atom_site_disorder_group  #         10 Check
PLAT822_ALERT_4_G CIF-embedded .res Contains Negative PART Numbers          2 Check
PLAT860_ALERT_3_G Number of Least-Squares Restraints .............        190 Note
PLAT912_ALERT_4_G Missing # of FCF Reflections Above STh/L=  0.600       4093 Note
PLAT913_ALERT_3_G Missing # of Very Strong Reflections in FCF ....          2 Note
PLAT933_ALERT_2_G Number of HKL-OMIT Records in Embedded .res File          4 Note
PLAT965_ALERT_2_G The SHELXL WEIGHT Optimisation has not Converged     Please Check
PLAT978_ALERT_2_G Number C-C Bonds with Positive Residual Density.          0 Info
```

```
   0 ALERT level A = Most likely a serious problem - resolve or explain
   0 ALERT level B = A potentially serious problem, consider carefully
  23 ALERT level C = Check. Ensure it is not caused by an omission or oversight
  38 ALERT level G = General information/check it is not something unexpected

   9 ALERT type 1 CIF construction/syntax error, inconsistent or missing data
  30 ALERT type 2 Indicator that the structure model may be wrong or deficient
   6 ALERT type 3 Indicator that the structure quality may be low
  16 ALERT type 4 Improvement, methodology, query or suggestion
   0 ALERT type 5 Informative message, check
```

It is advisable to attempt to resolve as many as possible of the alerts in all categories. Often the minor alerts point to easily fixed oversights, errors and omissions in your CIF or refinement strategy, so attention to these fine details can be worthwhile. In order to resolve some of the more serious problems it may be necessary to carry out additional measurements or structure refinements. However, the purpose of your study may justify the reported deviations and the more serious of these should normally be commented upon in the discussion or experimental section of a paper or in the "special_details" fields of the CIF. checkCIF was carefully designed to identify outliers and unusual parameters, but every test has its limitations and alerts that are not important in a particular case may appear. Conversely, the absence of alerts does not guarantee there are no aspects of the results needing attention. It is up to the individual to critically assess their own results and, if necessary, seek expert advice.

Publication of your CIF in IUCr journals

A basic structural check has been run on your CIF. These basic checks will be run on all CIFs submitted for publication in IUCr journals (*Acta Crystallographica*, *Journal of Applied Crystallography*, *Journal of Synchrotron Radiation*); however, if you intend to submit to *Acta Crystallographica Section C* or *E* or *IUCrData*, you should make sure that full publication checks are run on the final version of your CIF prior to submission.

Publication of your CIF in other journals

Please refer to the *Notes for Authors* of the relevant journal for any special instructions relating to CIF submission.

PLATON version of 06/07/2023; check.def file version of 30/06/2023

Datablock NC5LT - ellipsoid plot

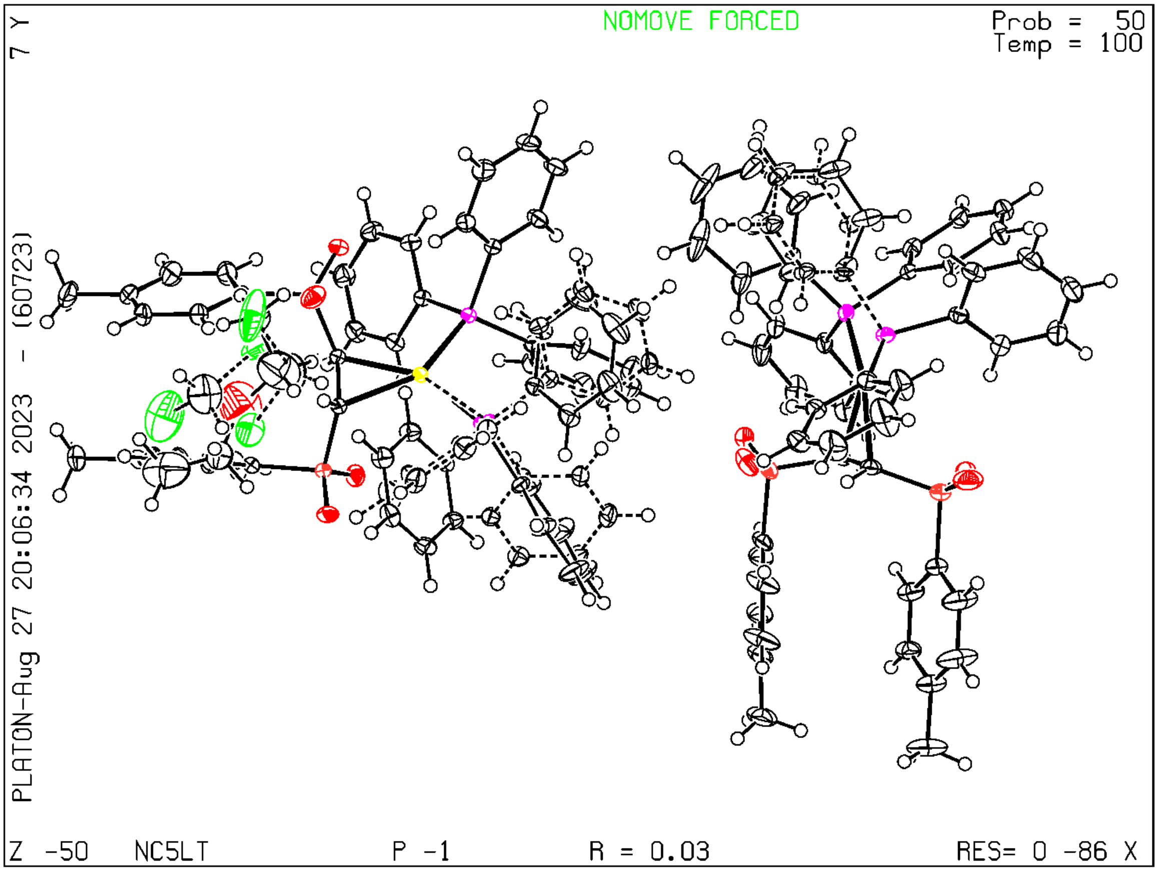

Supplement: Supplementary file 1 [file molecules-29-01119-s001.zip › 2aLT_checkcif.pdf]
