# Supplementary material for: Platinum(0)-η2-1,2-(E)ditosylethene Complexes Bearing Phosphine, Isocyanide and N-Heterocyclic Carbene Ligands: Synthesis and Cytotoxicity towards Ovarian and Breast Cancer Cells
Source: Molecules. 2024 Mar 1;29(5):1119. doi: 10.3390/molecules29051119 (PMC10934098; doi:10.3390/molecules29051119)

checkCIF/PLATON report

Structure factors have been supplied for datablock(s) NC5RT

THIS REPORT IS FOR GUIDANCE ONLY. IF USED AS PART OF A REVIEW PROCEDURE FOR PUBLICATION, IT SHOULD NOT REPLACE THE EXPERTISE OF AN EXPERIENCED CRYSTALLOGRAPHIC REFEREE.

No syntax errors found. CIF dictionary Interpreting this report

Datablock: NC5RT

Bond precision:	C-C = 0.0033 A		Wavelength=0.62000
Cell:	a=14.754(3)	b=18.270(4)	c=18.705(4)
	alpha=101.72(3)	beta=95.88(3)	gamma=98.88(3)
Temperature:	298 K		

	Calculated	Reported
Volume	4831(2)	4830.9(18)
Space group	P -1	P -1
Hall group	-P 1	-P 1
Moiety formula	C52 H46 O4 P2 Pt S2, 0.265(C4 H10 O), 0.229(C H2 Cl2)	C52 H46 O4 P2 Pt S2, 0.25 (C4 H10 O), 0.25 (C H2 Cl2)
Sum formula	C53.29 H49.11 Cl0.46 O4.27 P2 Pt S2	C53.25 H48.75 Cl0.50 O4.25 P2 Pt S2
Mr	1095.16	1095.54
Dx,g cm-3	1.506	1.506
Z	4	4
Mu (mm-1)	2.186	2.188
F000	2203.1	2203.0
F000'	2201.72	
h,k,lmax	25,31,31	24,29,29
Nref	49787	40702
Tmin,Tmax	0.877,0.957	
Tmin'	0.803	

Correction method= Not given

Data completeness= 0.818 Theta(max)= 31.827

```
R(reflections)= 0.0343( 34524)                           wR2(reflections)=
                                                         0.0940( 40702)
S = 1.055                      Npar= 1150
```

The following ALERTS were generated. Each ALERT has the format
test-name_ALERT_alert-type_alert-level.
Click on the hyperlinks for more details of the test.

Alert level C

```
PLAT041_ALERT_1_C Calc. and Reported SumFormula    Strings  Differ     Please Check
PLAT042_ALERT_1_C Calc. and Reported MoietyFormula Strings  Differ     Please Check
PLAT077_ALERT_4_C Unitcell Contains Non-integer Number of Atoms ..     Please Check
PLAT220_ALERT_2_C NonSolvent   Resd 1  C   Ueq(max)/Ueq(min) Range        3.5 Ratio
PLAT220_ALERT_2_C NonSolvent   Resd 2  C   Ueq(max)/Ueq(min) Range        3.2 Ratio
PLAT234_ALERT_4_C Large Hirshfeld Difference C16B_24  --C15B_24  .       0.16 Ang.
PLAT241_ALERT_2_C High   'MainMol' Ueq as Compared to Neighbors of      C5_12 Check
PLAT241_ALERT_2_C High   'MainMol' Ueq as Compared to Neighbors of     C12_12 Check
PLAT241_ALERT_2_C High   'MainMol' Ueq as Compared to Neighbors of     C15_14 Check
PLAT241_ALERT_2_C High   'MainMol' Ueq as Compared to Neighbors of      C9_14 Check
PLAT242_ALERT_2_C Low    'MainMol' Ueq as Compared to Neighbors of      C6_22 Check
PLAT242_ALERT_2_C Low    'MainMol' Ueq as Compared to Neighbors of      C6_12 Check
PLAT242_ALERT_2_C Low    'MainMol' Ueq as Compared to Neighbors of     C13_12 Check
PLAT242_ALERT_2_C Low    'MainMol' Ueq as Compared to Neighbors of     C16_14 Check
PLAT242_ALERT_2_C Low    'MainMol' Ueq as Compared to Neighbors of     C13_14 Check
PLAT260_ALERT_2_C Large Average Ueq of Residue Including     O1_91      0.205 Check
PLAT260_ALERT_2_C Large Average Ueq of Residue Including   Cl1A_92      0.247 Check
PLAT260_ALERT_2_C Large Average Ueq of Residue Including   Cl1B_92      0.241 Check
PLAT911_ALERT_3_C Missing FCF Refl Between Thmin & STh/L=    0.600        335 Report
PLAT972_ALERT_2_C Check Calcd Resid. Dens.  0.61Ang From Pt_11          -2.50 eA-3
PLAT972_ALERT_2_C Check Calcd Resid. Dens.  0.64Ang From Pt_11          -2.45 eA-3
PLAT972_ALERT_2_C Check Calcd Resid. Dens.  0.61Ang From Pt_21          -2.27 eA-3
PLAT972_ALERT_2_C Check Calcd Resid. Dens.  0.57Ang From Pt_21          -2.02 eA-3
PLAT972_ALERT_2_C Check Calcd Resid. Dens.  0.55Ang From Pt_21          -1.82 eA-3
PLAT972_ALERT_2_C Check Calcd Resid. Dens.  0.68Ang From Pt_21          -1.74 eA-3
PLAT975_ALERT_2_C Check Calcd Resid. Dens.  1.01Ang From O2_12   .       0.65 eA-3
```

Alert level G

```
FORMU01_ALERT_1_G  There is a discrepancy between the atom counts in the
            _chemical_formula_sum and _chemical_formula_moiety. This is
            usually due to the moiety formula being in the wrong format.
            Atom count from _chemical_formula_sum:   C53.25 H48.75 Cl0.5 O4.25 P2
            Atom count from _chemical_formula_moiety:C53.25 H49 Cl0.5 O4.25 P2 Pt1
FORMU01_ALERT_2_G  There is a discrepancy between the atom counts in the
            _chemical_formula_sum and the formula from the _atom_site* data.
            Atom count from _chemical_formula_sum:C53.25 H48.75 Cl0.5 O4.25 P2 Pt1
            Atom count from the _atom_site data:  C53.28950 H49.10900 Cl0.459 O4.2
ABSMU01_ALERT_1_G  Calculation of _exptl_absorpt_correction_mu
                not performed for this radiation type.
CELLZ01_ALERT_1_G Difference between formula and atom_site contents detected.
CELLZ01_ALERT_1_G ALERT: Large difference may be due to a
            symmetry error - see SYMMG tests
           From the CIF: _cell_formula_units_Z    4
           From the CIF: _chemical_formula_sum  C53.25 H48.75 Cl0.50 O4.25 P2 Pt S
```

```
TEST: Compare cell contents of formula and atom_site data

atom    Z*formula  cif sites diff
C        213.00    213.16   -0.16
H        195.00    196.44   -1.44
Cl         2.00      1.84    0.16
O         17.00     17.06   -0.06
P          8.00      8.00    0.00
Pt         4.00      4.00    0.00
S          8.00      8.00    0.00
PLAT002_ALERT_2_G Number of Distance or Angle Restraints on AtSite         11 Note
PLAT003_ALERT_2_G Number of Uiso or Uij Restrained non-H Atoms ...          6 Report
PLAT092_ALERT_4_G Check: Wavelength Given is not Cu,Ga,Mo,Ag,In Ka    0.62000 Ang.
PLAT152_ALERT_1_G The Supplied and Calc. Volume s.u. Differ by ...          2 Units
PLAT154_ALERT_1_G The s.u.'s on the Cell Angles are Equal ..(Note)       0.03 Degree
PLAT172_ALERT_4_G The CIF-Embedded .res File Contains DFIX Records          3 Report
PLAT173_ALERT_4_G The CIF-Embedded .res File Contains DANG Records          3 Report
PLAT175_ALERT_4_G The CIF-Embedded .res File Contains SAME Records          1 Report
PLAT176_ALERT_4_G The CIF-Embedded .res File Contains SADI Records          2 Report
PLAT178_ALERT_4_G The CIF-Embedded .res File Contains SIMU Records          1 Report
PLAT188_ALERT_3_G A Non-default SIMU Restraint Value has been used     0.0100 Report
PLAT301_ALERT_3_G Main Residue  Disorder .............(Resd  1  )        31% Note
PLAT302_ALERT_4_G Anion/Solvent/Minor-Residue Disorder (Resd  3  )       100% Note
PLAT302_ALERT_4_G Anion/Solvent/Minor-Residue Disorder (Resd  4  )       100% Note
PLAT302_ALERT_4_G Anion/Solvent/Minor-Residue Disorder (Resd  5  )       100% Note
PLAT304_ALERT_4_G Non-Integer Number of Atoms in ..... (Resd  3  )       7.95 Check
PLAT304_ALERT_4_G Non-Integer Number of Atoms in ..... (Resd  4  )       1.13 Check
PLAT304_ALERT_4_G Non-Integer Number of Atoms in ..... (Resd  5  )       1.16 Check
PLAT343_ALERT_2_G Unusual sp?      Angle Range in Main Residue for      C2_22 Check
PLAT411_ALERT_2_G Short Inter H...H Contact  H11_14   ..H9A_24   .       2.10 Ang.
                                                 1-x,1-y,1-z  =      2_666 Check
PLAT413_ALERT_2_G Short Inter XH3 .. XHn     H4A3_91  ..H4_22    .       2.14 Ang.
                                                       x,y,z  =      1_555 Check
PLAT720_ALERT_4_G Number of Unusual/Non-Standard Labels .........         273 Note
PLAT721_ALERT_1_G Bond    Calc     0.96000, Rep     0.97000 Dev...       0.01 Ang.
            C1B_92   -H1D_92          1_555   1_555 ........     #   22 Check
PLAT789_ALERT_4_G Atoms with Negative _atom_site_disorder_group  #         10 Check
PLAT822_ALERT_4_G CIF-embedded .res Contains Negative PART Numbers          2 Check
PLAT860_ALERT_3_G Number of Least-Squares Restraints ............          70 Note
PLAT912_ALERT_4_G Missing # of FCF Reflections Above STh/L=  0.600       8175 Note
PLAT933_ALERT_2_G Number of HKL-OMIT Records in Embedded .res File         35 Note
PLAT951_ALERT_5_G Calculated (ThMax) and CIF-Reported Kmax Differ           2 Units
PLAT952_ALERT_5_G Calculated (ThMax) and CIF-Reported Lmax Differ.          2 Units
PLAT957_ALERT_1_G Calculated (ThMax) and Actual (FCF) Kmax Differ           2 Units
PLAT958_ALERT_1_G Calculated (ThMax) and Actual (FCF) Lmax Differ.          2 Units
PLAT965_ALERT_2_G The SHELXL WEIGHT Optimisation has not Converged     Please Check
PLAT978_ALERT_2_G Number C-C Bonds with Positive Residual Density.          0 Info
```

---

0 **ALERT level A** = Most likely a serious problem - resolve or explain
0 **ALERT level B** = A potentially serious problem, consider carefully
26 **ALERT level C** = Check. Ensure it is not caused by an omission or oversight
39 **ALERT level G** = General information/check it is not something unexpected

11 ALERT type 1 CIF construction/syntax error, inconsistent or missing data

```
30 ALERT type 2 Indicator that the structure model may be wrong or deficient
 4 ALERT type 3 Indicator that the structure quality may be low
18 ALERT type 4 Improvement, methodology, query or suggestion
 2 ALERT type 5 Informative message, check
```

It is advisable to attempt to resolve as many as possible of the alerts in all categories. Often the minor alerts point to easily fixed oversights, errors and omissions in your CIF or refinement strategy, so attention to these fine details can be worthwhile. In order to resolve some of the more serious problems it may be necessary to carry out additional measurements or structure refinements. However, the purpose of your study may justify the reported deviations and the more serious of these should normally be commented upon in the discussion or experimental section of a paper or in the "special_details" fields of the CIF. checkCIF was carefully designed to identify outliers and unusual parameters, but every test has its limitations and alerts that are not important in a particular case may appear. Conversely, the absence of alerts does not guarantee there are no aspects of the results needing attention. It is up to the individual to critically assess their own results and, if necessary, seek expert advice.

Publication of your CIF in IUCr journals

A basic structural check has been run on your CIF. These basic checks will be run on all CIFs submitted for publication in IUCr journals (*Acta Crystallographica*, *Journal of Applied Crystallography*, *Journal of Synchrotron Radiation*); however, if you intend to submit to *Acta Crystallographica Section C* or *E* or *IUCrData*, you should make sure that full publication checks are run on the final version of your CIF prior to submission.

Publication of your CIF in other journals

Please refer to the *Notes for Authors* of the relevant journal for any special instructions relating to CIF submission.

PLATON version of 06/07/2023; check.def file version of 30/06/2023

Datablock NC5RT - ellipsoid plot

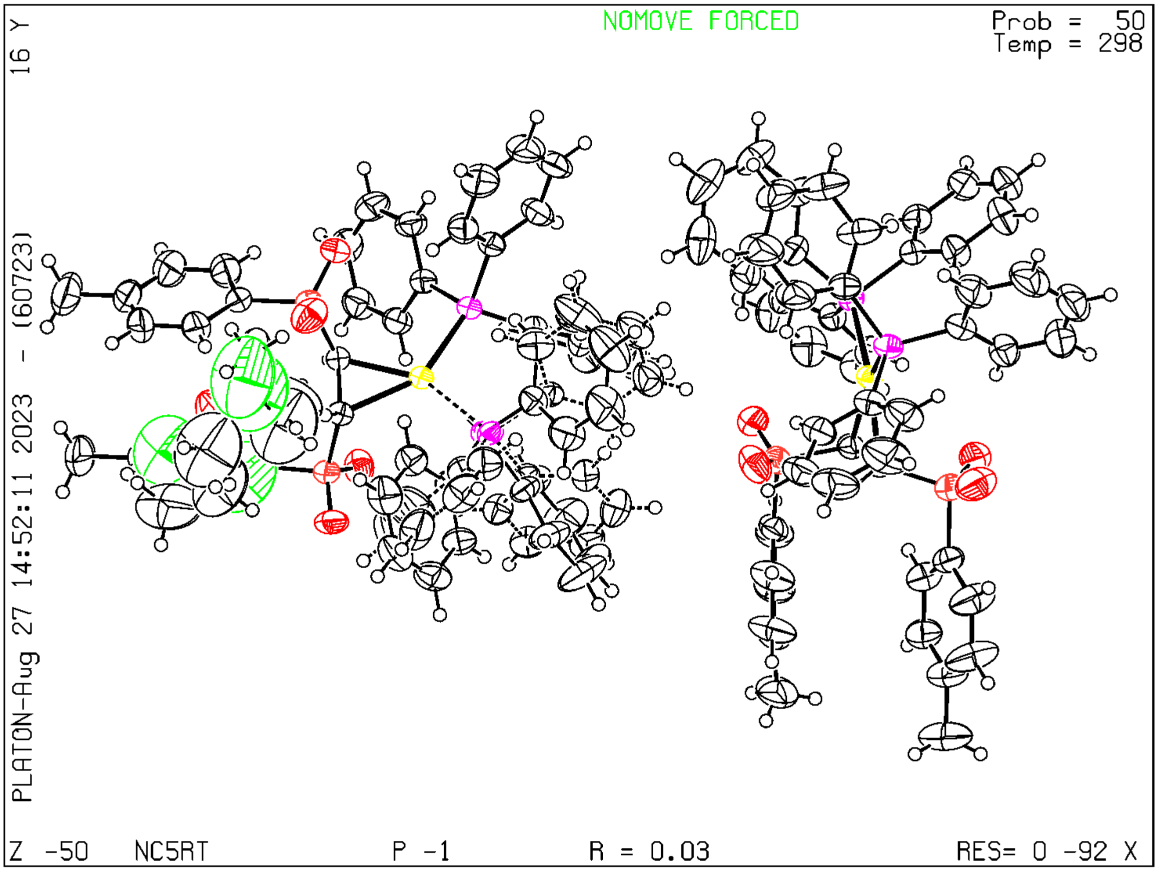

Supplement: Supplementary file 1 [file molecules-29-01119-s001.zip › 2aRT_checkcif.pdf]
