# Supplementary material for: Platinum(0)-η2-1,2-(E)ditosylethene Complexes Bearing Phosphine, Isocyanide and N-Heterocyclic Carbene Ligands: Synthesis and Cytotoxicity towards Ovarian and Breast Cancer Cells
Source: Molecules. 2024 Mar 1;29(5):1119. doi: 10.3390/molecules29051119 (PMC10934098; doi:10.3390/molecules29051119)

checkCIF/PLATON report

Structure factors have been supplied for datablock(s) NC1

THIS REPORT IS FOR GUIDANCE ONLY. IF USED AS PART OF A REVIEW PROCEDURE FOR PUBLICATION, IT SHOULD NOT REPLACE THE EXPERTISE OF AN EXPERIENCED CRYSTALLOGRAPHIC REFEREE.

No syntax errors found. CIF dictionary Interpreting this report

Datablock: NC1

```
Bond precision:   C-C = 0.0044 A          Wavelength=0.62000

Cell:              a=15.496(3)     b=9.010(2)          c=23.067(5)
                   alpha=90        beta=101.56(3)      gamma=90
Temperature:       100 K

                 Calculated                  Reported
Volume           3155.3(12)                  3155.2(11)
Space group      P 21/c                      P 21/c
Hall group       -P 2ybc                     -P 2ybc
Moiety formula   C34 H34 N2 O4 Pt S2         C34 H34 N2 O4 Pt S2
Sum formula      C34 H34 N2 O4 Pt S2         C34 H34 N2 O4 Pt S2
Mr               793.83                      793.84
Dx,g cm-3        1.671                       1.671
Z                4                           4
Mu (mm-1)        3.232                       3.233
F000             1576.0                      1576.0
F000'            1573.73
h,k,lmax         22,13,33                    22,13,33
Nref             10516                       10435
Tmin,Tmax        0.824,0.937
Tmin'            0.724

Correction method= Not given

Data completeness= 0.992          Theta(max)= 27.122

                                                         wR2(reflections)=
R(reflections)= 0.0289( 9184)                            0.0767( 10435)

S = 1.105                  Npar= 394
```

The following ALERTS were generated. Each ALERT has the format
test-name_ALERT_alert-type_alert-level.
Click on the hyperlinks for more details of the test.

Alert level C

```
PLAT057_ALERT_3_C Correction for Absorption Required   RT(exp) ...        1.14 Do !
PLAT911_ALERT_3_C Missing FCF Refl Between Thmin & STh/L=    0.600          65 Report
PLAT971_ALERT_2_C Check Calcd Resid. Dens.  0.69Ang From Pt_1             1.96 eA-3
PLAT971_ALERT_2_C Check Calcd Resid. Dens.  0.84Ang From Pt_1             1.96 eA-3
PLAT971_ALERT_2_C Check Calcd Resid. Dens.  0.91Ang From Pt_1             1.90 eA-3
PLAT971_ALERT_2_C Check Calcd Resid. Dens.  0.69Ang From Pt_1             1.64 eA-3
PLAT972_ALERT_2_C Check Calcd Resid. Dens.  0.68Ang From Pt_1            -2.50 eA-3
PLAT972_ALERT_2_C Check Calcd Resid. Dens.  0.51Ang From Pt_1            -1.94 eA-3
PLAT972_ALERT_2_C Check Calcd Resid. Dens.  0.66Ang From Pt_1            -1.87 eA-3
PLAT972_ALERT_2_C Check Calcd Resid. Dens.  0.56Ang From Pt_1            -1.76 eA-3
PLAT972_ALERT_2_C Check Calcd Resid. Dens.  0.73Ang From Pt_1            -1.60 eA-3
PLAT972_ALERT_2_C Check Calcd Resid. Dens.  0.78Ang From Pt_1            -1.54 eA-3
PLAT977_ALERT_2_C Check Negative Difference Density on H8_2      .       -0.55 eA-3
```

Alert level G

```
ABSMU01_ALERT_1_G  Calculation of _exptl_absorpt_correction_mu
                not performed for this radiation type.
PLAT019_ALERT_1_G _diffrn_measured_fraction_theta_full/*_max < 1.0      0.997 Report
PLAT092_ALERT_4_G Check: Wavelength Given is not Cu,Ga,Mo,Ag,In Ka    0.62000 Ang.
PLAT232_ALERT_2_G Hirshfeld Test Diff (M-X)  Pt_1     --C_4       .       7.1 s.u.
PLAT720_ALERT_4_G Number of Unusual/Non-Standard Labels .........          77 Note
PLAT794_ALERT_5_G Tentative Bond Valency for Pt_1      (II)       .       2.04 Info
PLAT912_ALERT_4_G Missing # of FCF Reflections Above STh/L=  0.600         16 Note
PLAT933_ALERT_2_G Number of HKL-OMIT Records in Embedded .res File         46 Note
PLAT965_ALERT_2_G The SHELXL WEIGHT Optimisation has not Converged     Please Check
PLAT978_ALERT_2_G Number C-C Bonds with Positive Residual Density.          5 Info
PLAT984_ALERT_1_G The  N-f'=    0.0061 Deviates from the B&C-Value     0.0042 Check
```

0 **ALERT level A** = Most likely a serious problem - resolve or explain
0 **ALERT level B** = A potentially serious problem, consider carefully
13 **ALERT level C** = Check. Ensure it is not caused by an omission or oversight
11 **ALERT level G** = General information/check it is not something unexpected

3 ALERT type 1 CIF construction/syntax error, inconsistent or missing data
15 ALERT type 2 Indicator that the structure model may be wrong or deficient
2 ALERT type 3 Indicator that the structure quality may be low
3 ALERT type 4 Improvement, methodology, query or suggestion
1 ALERT type 5 Informative message, check

It is advisable to attempt to resolve as many as possible of the alerts in all categories. Often the minor alerts point to easily fixed oversights, errors and omissions in your CIF or refinement strategy, so attention to these fine details can be worthwhile. In order to resolve some of the more serious problems it may be necessary to carry out additional measurements or structure refinements. However, the purpose of your study may justify the reported deviations and the more serious of these should normally be commented upon in the discussion or experimental section of a paper or in the "special_details" fields of the CIF. checkCIF was carefully designed to identify outliers and unusual parameters, but every test has its limitations and alerts that are not important in a particular case may appear. Conversely, the absence of alerts does not guarantee there are no aspects of the results needing attention. It is up to the individual to critically assess their own results and, if necessary, seek expert advice.

Publication of your CIF in IUCr journals

A basic structural check has been run on your CIF. These basic checks will be run on all CIFs submitted for publication in IUCr journals (*Acta Crystallographica*, *Journal of Applied Crystallography*, *Journal of Synchrotron Radiation*); however, if you intend to submit to *Acta Crystallographica Section C* or *E* or *IUCrData*, you should make sure that full publication checks are run on the final version of your CIF prior to submission.

Publication of your CIF in other journals

Please refer to the *Notes for Authors* of the relevant journal for any special instructions relating to CIF submission.

PLATON version of 06/07/2023; check.def file version of 30/06/2023

Datablock NC1 - ellipsoid plot

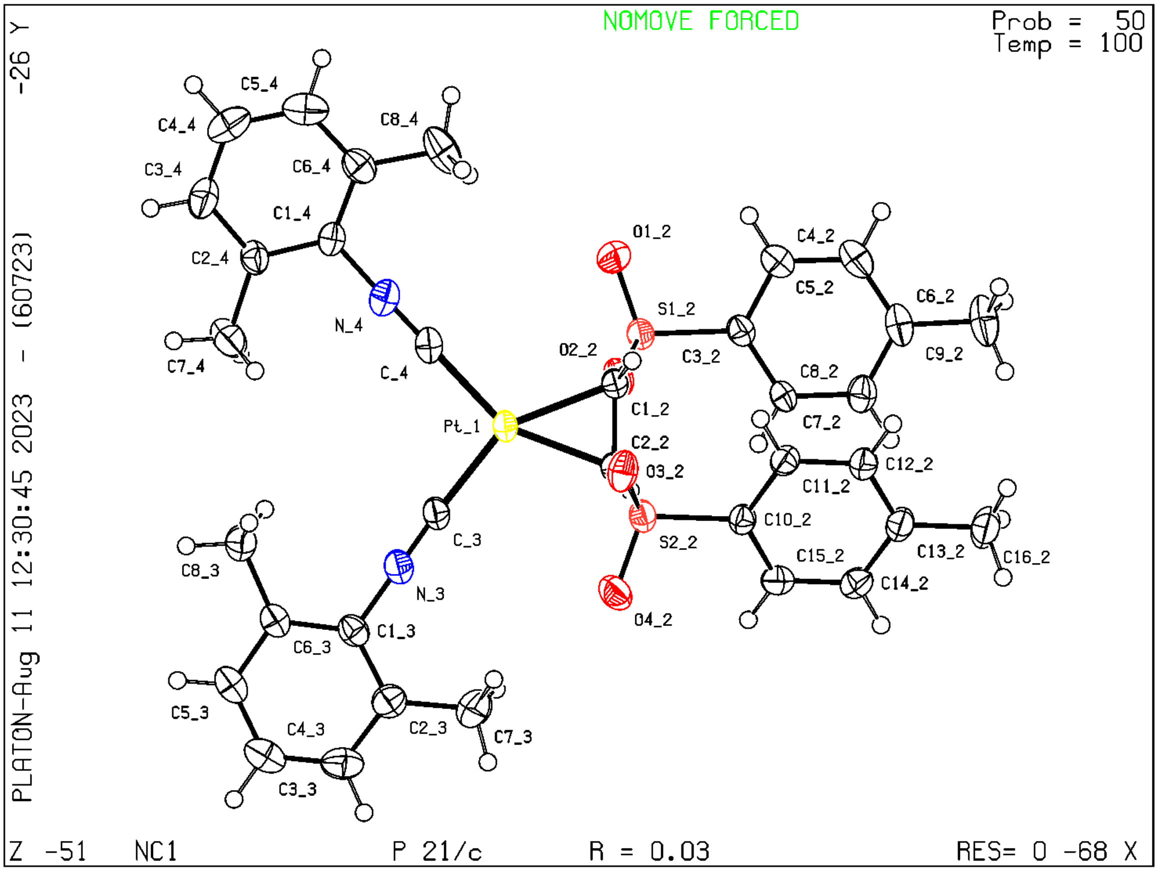

Supplement: Supplementary file 1 [file molecules-29-01119-s001.zip › 3a_checkcif.pdf]
