# Supplementary material for: Platinum(0)-η2-1,2-(E)ditosylethene Complexes Bearing Phosphine, Isocyanide and N-Heterocyclic Carbene Ligands: Synthesis and Cytotoxicity towards Ovarian and Breast Cancer Cells
Source: Molecules. 2024 Mar 1;29(5):1119. doi: 10.3390/molecules29051119 (PMC10934098; doi:10.3390/molecules29051119)

checkCIF/PLATON report

Structure factors have been supplied for datablock(s) NC3

THIS REPORT IS FOR GUIDANCE ONLY. IF USED AS PART OF A REVIEW PROCEDURE FOR PUBLICATION, IT SHOULD NOT REPLACE THE EXPERTISE OF AN EXPERIENCED CRYSTALLOGRAPHIC REFEREE.

No syntax errors found. CIF dictionary Interpreting this report

Datablock: NC3

Bond precision: C-C = 0.0028 A Wavelength=0.62000

Cell:	a=10.295(2)	b=13.985(3)	c=14.334(3)
	alpha=62.54(3)	beta=80.47(3)	gamma=75.95(3)
Temperature:	100 K		

	Calculated	Reported
Volume	1773.0(8)	1773.1(8)
Space group	P -1	P -1
Hall group	-P 1	-P 1
Moiety formula	C34 H34 N2 O8 Pt S4	C34 H34 N2 O8 Pt S4
Sum formula	C34 H34 N2 O8 Pt S4	C34 H34 N2 O8 Pt S4
Mr	921.95	921.96
Dx,g cm-3	1.727	1.727
Z	2	2
Mu (mm-1)	2.966	2.966
F000	916.0	916.0
F000'	915.31	
h,k,lmax	15,21,21	15,21,21
Nref	12924	12650
Tmin,Tmax	0.837,0.942	
Tmin'	0.743	

Correction method= Not given

Data completeness= 0.979 Theta(max)= 28.014

R(reflections)= 0.0215(12023) wR2(reflections)= 0.0538(12650)

S = 1.038 Npar= 446

The following ALERTS were generated. Each ALERT has the format

test-name_ALERT_alert-type_alert-level.

Click on the hyperlinks for more details of the test.

Alert level C

```
PLAT057_ALERT_3_C Correction for Absorption Required   RT(exp) ...        1.13 Do !
PLAT220_ALERT_2_C NonSolvent   Resd 1  C   Ueq(max)/Ueq(min) Range         3.3 Ratio
PLAT222_ALERT_3_C NonSolvent Resd 1  H   Uiso(max)/Uiso(min) Range         4.2 Ratio
PLAT911_ALERT_3_C Missing FCF Refl Between Thmin & STh/L=    0.600         165 Report
PLAT972_ALERT_2_C Check Calcd Resid. Dens.  0.71Ang From Pt_1            -2.50 eA-3
PLAT975_ALERT_2_C Check Calcd Resid. Dens.  0.99Ang From C2_2    .        0.40 eA-3
PLAT977_ALERT_2_C Check Negative Difference Density on H9A_2     .       -0.31 eA-3
```

Alert level G

```
ABSMU01_ALERT_1_G  Calculation of _exptl_absorpt_correction_mu
                not performed for this radiation type.
PLAT019_ALERT_1_G _diffrn_measured_fraction_theta_full/*_max < 1.0      0.995 Report
PLAT092_ALERT_4_G Check: Wavelength Given is not Cu,Ga,Mo,Ag,In Ka    0.62000 Ang.
PLAT154_ALERT_1_G The s.u.'s on the Cell Angles are Equal ..(Note)       0.03 Degree
PLAT232_ALERT_2_G Hirshfeld Test Diff (M-X)  Pt_1     --C_3      .        5.9 s.u.
PLAT232_ALERT_2_G Hirshfeld Test Diff (M-X)  Pt_1     --C_4      .        7.3 s.u.
PLAT343_ALERT_2_G Unusual sp?      Angle Range in Main Residue for       C2_2 Check
PLAT720_ALERT_4_G Number of Unusual/Non-Standard Labels .........          83 Note
PLAT794_ALERT_5_G Tentative Bond Valency for Pt_1      (II)      .       2.04 Info
PLAT910_ALERT_3_G Missing # of FCF Reflection(s) Below Theta(Min).          1 Note
PLAT912_ALERT_4_G Missing # of FCF Reflections Above STh/L=  0.600        111 Note
PLAT933_ALERT_2_G Number of HKL-OMIT Records in Embedded .res File         13 Note
PLAT978_ALERT_2_G Number C-C Bonds with Positive Residual Density.          7 Info
```

0 **ALERT level A** = Most likely a serious problem - resolve or explain
0 **ALERT level B** = A potentially serious problem, consider carefully
7 **ALERT level C** = Check. Ensure it is not caused by an omission or oversight
13 **ALERT level G** = General information/check it is not something unexpected

3 ALERT type 1 CIF construction/syntax error, inconsistent or missing data
9 ALERT type 2 Indicator that the structure model may be wrong or deficient
4 ALERT type 3 Indicator that the structure quality may be low
3 ALERT type 4 Improvement, methodology, query or suggestion
1 ALERT type 5 Informative message, check

It is advisable to attempt to resolve as many as possible of the alerts in all categories. Often the minor alerts point to easily fixed oversights, errors and omissions in your CIF or refinement strategy, so attention to these fine details can be worthwhile. In order to resolve some of the more serious problems it may be necessary to carry out additional measurements or structure refinements. However, the purpose of your study may justify the reported deviations and the more serious of these should normally be commented upon in the discussion or experimental section of a paper or in the "special_details" fields of the CIF. checkCIF was carefully designed to identify outliers and unusual parameters, but every test has its limitations and alerts that are not important in a particular case may appear. Conversely, the absence of alerts does not guarantee there are no aspects of the results needing attention. It is up to the individual to critically assess their own results and, if necessary, seek expert advice.

Publication of your CIF in IUCr journals

A basic structural check has been run on your CIF. These basic checks will be run on all CIFs submitted for publication in IUCr journals (*Acta Crystallographica*, *Journal of Applied Crystallography*, *Journal of Synchrotron Radiation*); however, if you intend to submit to *Acta Crystallographica Section C* or *E* or *IUCrData*, you should make sure that full publication checks are run on the final version of your CIF prior to submission.

Publication of your CIF in other journals

Please refer to the *Notes for Authors* of the relevant journal for any special instructions relating to CIF submission.

PLATON version of 06/07/2023; check.def file version of 30/06/2023

Datablock NC3 - ellipsoid plot

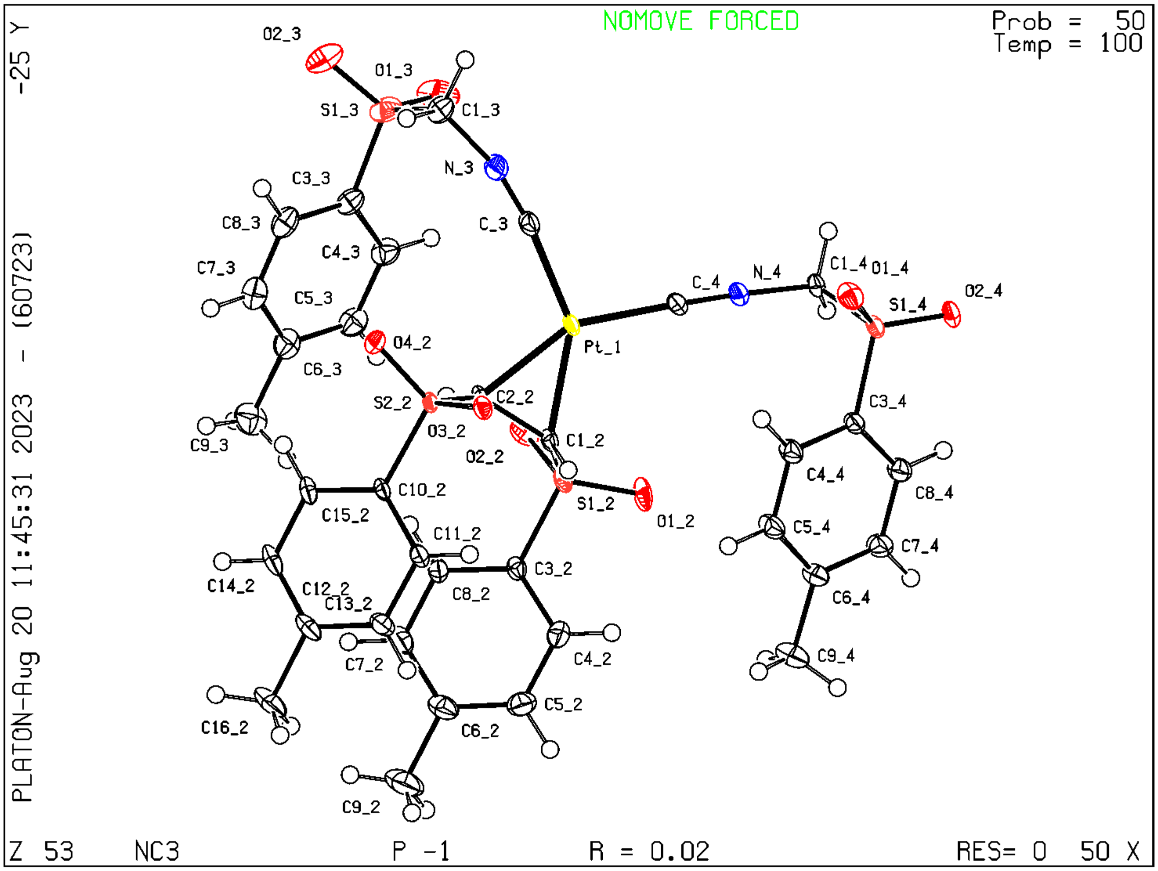

Supplement: Supplementary file 1 [file molecules-29-01119-s001.zip › 3b_checkcif.pdf]
