# Supplementary material for: Platinum(0)-η2-1,2-(E)ditosylethene Complexes Bearing Phosphine, Isocyanide and N-Heterocyclic Carbene Ligands: Synthesis and Cytotoxicity towards Ovarian and Breast Cancer Cells
Source: Molecules. 2024 Mar 1;29(5):1119. doi: 10.3390/molecules29051119 (PMC10934098; doi:10.3390/molecules29051119)

checkCIF/PLATON report

Structure factors have been supplied for datablock(s) NC9

THIS REPORT IS FOR GUIDANCE ONLY. IF USED AS PART OF A REVIEW PROCEDURE FOR PUBLICATION, IT SHOULD NOT REPLACE THE EXPERTISE OF AN EXPERIENCED CRYSTALLOGRAPHIC REFEREE.

No syntax errors found. CIF dictionary Interpreting this report

Datablock: NC9

```
Bond precision:   C-C = 0.0067 A          Wavelength=0.62000

Cell:            a=11.488(2)        b=14.203(3)       c=14.404(3)
                 alpha=62.21(3)     beta=75.71(3)     gamma=74.35(3)
Temperature:     100 K

                Calculated                    Reported
Volume          1981.8(9)                     1981.8(9)
Space group     P 1                           P 1
Hall group      P 1                           P 1
Moiety formula  C43 H37 F3 N O4 P Pt S2       C43 H37 F3 N O4 P Pt S2
Sum formula     C43 H37 F3 N O4 P Pt S2       C43 H37 F3 N O4 P Pt S2
Mr              978.91                        978.91
Dx,g cm-3       1.640                         1.640
Z               2                             2
Mu (mm-1)       2.618                         2.618
F000            972.0                         972.0
F000'           971.10
h,k,lmax        18,22,23                      18,22,23
Nref            34848[ 17424]                 31365
Tmin,Tmax       0.939,0.949
Tmin'           0.877

Correction method= Not given

Data completeness= 1.80/0.90       Theta(max)= 29.999

                                                    wR2(reflections)=
R(reflections)= 0.0277( 29616)                      0.0628( 31365)

S = 1.109                  Npar= 1000
```

The following ALERTS were generated. Each ALERT has the format
test-name_ALERT_alert-type_alert-level.
Click on the hyperlinks for more details of the test.

Alert level C

```
PLAT220_ALERT_2_C NonSolvent   Resd 2  C   Ueq(max)/Ueq(min) Range        3.1 Ratio
PLAT911_ALERT_3_C Missing FCF Refl Between Thmin & STh/L=    0.600          8 Report
PLAT971_ALERT_2_C Check Calcd Resid. Dens.  0.61Ang From Pt_21           1.64 eA-3
PLAT971_ALERT_2_C Check Calcd Resid. Dens.  0.68Ang From Pt_11           1.57 eA-3
PLAT971_ALERT_2_C Check Calcd Resid. Dens.  0.51Ang From Pt_21           1.53 eA-3
PLAT972_ALERT_2_C Check Calcd Resid. Dens.  0.63Ang From Pt_21          -2.42 eA-3
PLAT972_ALERT_2_C Check Calcd Resid. Dens.  0.63Ang From Pt_11          -2.27 eA-3
PLAT972_ALERT_2_C Check Calcd Resid. Dens.  0.62Ang From Pt_21          -2.22 eA-3
PLAT972_ALERT_2_C Check Calcd Resid. Dens.  0.63Ang From Pt_11          -2.18 eA-3
PLAT972_ALERT_2_C Check Calcd Resid. Dens.  0.63Ang From Pt_11          -1.90 eA-3
PLAT972_ALERT_2_C Check Calcd Resid. Dens.  0.58Ang From Pt_21          -1.57 eA-3
PLAT977_ALERT_2_C Check Negative Difference Density on H5_13     .      -0.36 eA-3
```

Alert level G

```
ABSMU01_ALERT_1_G  Calculation of _exptl_absorpt_correction_mu
                not performed for this radiation type.
PLAT033_ALERT_4_G Flack x Value Deviates > 3.0 * sigma from Zero .      0.299 Note
PLAT092_ALERT_4_G Check: Wavelength Given is not Cu,Ga,Mo,Ag,In Ka    0.62000 Ang.
PLAT154_ALERT_1_G The s.u.'s on the Cell Angles are Equal ..(Note)       0.03 Degree
PLAT343_ALERT_2_G Unusual sp?      Angle Range in Main Residue for      C1_12 Check
PLAT343_ALERT_2_G Unusual sp?      Angle Range in Main Residue for      C2_12 Check
PLAT343_ALERT_2_G Unusual sp?      Angle Range in Main Residue for      C1_22 Check
PLAT343_ALERT_2_G Unusual sp?      Angle Range in Main Residue for      C2_22 Check
PLAT380_ALERT_4_G Incorrectly? Oriented X(sp2)-Methyl Moiety .....      C7_13 Check
PLAT720_ALERT_4_G Number of Unusual/Non-Standard Labels ..........        184 Note
PLAT912_ALERT_4_G Missing # of FCF Reflections Above STh/L=  0.600        839 Note
PLAT933_ALERT_2_G Number of HKL-OMIT Records in Embedded .res File         19 Note
PLAT941_ALERT_3_G Average HKL Measurement Multiplicity ..........         4.2 Low
PLAT978_ALERT_2_G Number C-C Bonds with Positive Residual Density.           0 Info
```

0 **ALERT level A** = Most likely a serious problem - resolve or explain
0 **ALERT level B** = A potentially serious problem, consider carefully
12 **ALERT level C** = Check. Ensure it is not caused by an omission or oversight
14 **ALERT level G** = General information/check it is not something unexpected

2 ALERT type 1 CIF construction/syntax error, inconsistent or missing data
17 ALERT type 2 Indicator that the structure model may be wrong or deficient
2 ALERT type 3 Indicator that the structure quality may be low
5 ALERT type 4 Improvement, methodology, query or suggestion
0 ALERT type 5 Informative message, check

It is advisable to attempt to resolve as many as possible of the alerts in all categories. Often the minor alerts point to easily fixed oversights, errors and omissions in your CIF or refinement strategy, so attention to these fine details can be worthwhile. In order to resolve some of the more serious problems it may be necessary to carry out additional measurements or structure refinements. However, the purpose of your study may justify the reported deviations and the more serious of these should normally be commented upon in the discussion or experimental section of a paper or in the "special_details" fields of the CIF. checkCIF was carefully designed to identify outliers and unusual parameters, but every test has its limitations and alerts that are not important in a particular case may appear. Conversely, the absence of alerts does not guarantee there are no aspects of the results needing attention. It is up to the individual to critically assess their own results and, if necessary, seek expert advice.

Publication of your CIF in IUCr journals

A basic structural check has been run on your CIF. These basic checks will be run on all CIFs submitted for publication in IUCr journals (*Acta Crystallographica*, *Journal of Applied Crystallography*, *Journal of Synchrotron Radiation*); however, if you intend to submit to *Acta Crystallographica Section C* or *E* or *IUCrData*, you should make sure that full publication checks are run on the final version of your CIF prior to submission.

Publication of your CIF in other journals

Please refer to the *Notes for Authors* of the relevant journal for any special instructions relating to CIF submission.

PLATON version of 06/07/2023; check.def file version of 30/06/2023

Datablock NC9 - ellipsoid plot

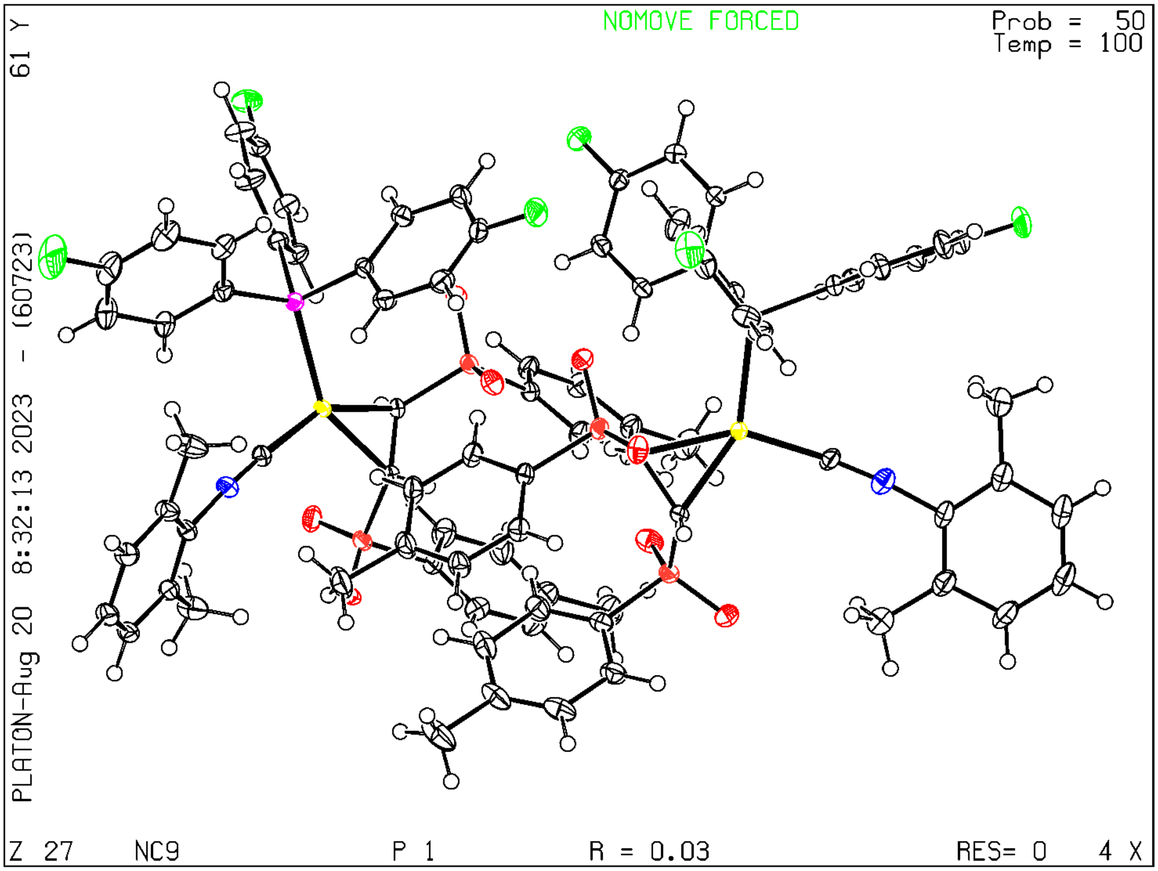

Supplement: Supplementary file 1 [file molecules-29-01119-s001.zip › 4b_checkcif.pdf]
