# Supplementary material for: Platinum(0)-η2-1,2-(E)ditosylethene Complexes Bearing Phosphine, Isocyanide and N-Heterocyclic Carbene Ligands: Synthesis and Cytotoxicity towards Ovarian and Breast Cancer Cells
Source: Molecules. 2024 Mar 1;29(5):1119. doi: 10.3390/molecules29051119 (PMC10934098; doi:10.3390/molecules29051119)

checkCIF/PLATON report

Structure factors have been supplied for datablock(s) NC16

THIS REPORT IS FOR GUIDANCE ONLY. IF USED AS PART OF A REVIEW PROCEDURE FOR PUBLICATION, IT SHOULD NOT REPLACE THE EXPERTISE OF AN EXPERIENCED CRYSTALLOGRAPHIC REFEREE.

No syntax errors found. CIF dictionary Interpreting this report

Datablock: NC16

Bond precision: C-C = 0.0069 A Wavelength=0.62000

Cell:	a=10.808(2)	b=11.808(2)	c=15.799(3)
	alpha=74.17(3)	beta=70.02(3)	gamma=76.02(3)
Temperature:	100 K		

	Calculated	Reported
Volume	1798.4(7)	1798.5(8)
Space group	P -1	P -1
Hall group	-P 1	-P 1
Moiety formula	C38 H40 N4 O4 Pt S2	C38 H40 N4 O4 Pt S2
Sum formula	C38 H40 N4 O4 Pt S2	C38 H40 N4 O4 Pt S2
Mr	875.94	875.95
Dx,g cm-3	1.618	1.618
Z	2	2
Mu (mm-1)	2.843	2.843
F000	876.0	876.0
F000’	874.86	
h,k,lmax	12,13,18	12,13,18
Nref	5832	5731
Tmin,Tmax	0.843,0.945	
Tmin’	0.753	

Correction method= Not given

Data completeness= 0.983 Theta(max)= 21.000

R(reflections)= 0.0260(5440) wR2(reflections)= 0.0636(5731)

S = 1.074 Npar= 446

The following ALERTS were generated. Each ALERT has the format
test-name_ALERT_alert-type_alert-level.
Click on the hyperlinks for more details of the test.

Alert level C

```
THETM01_ALERT_3_C  The value of sine(theta_max)/wavelength is less than 0.590
            Calculated sin(theta_max)/wavelength =    0.5780
PLAT057_ALERT_3_C Correction for Absorption Required   RT(exp) ...       1.12 Do !
PLAT220_ALERT_2_C NonSolvent   Resd 1  C   Ueq(max)/Ueq(min) Range        3.5 Ratio
PLAT911_ALERT_3_C Missing FCF Refl Between Thmin & STh/L=    0.578        101 Report
PLAT971_ALERT_2_C Check Calcd Resid. Dens.  0.88Ang From Pt_1            2.50 eA-3
PLAT971_ALERT_2_C Check Calcd Resid. Dens.  1.00Ang From Pt_1            1.74 eA-3
```

Alert level G

```
ABSMU01_ALERT_1_G  Calculation of _exptl_absorpt_correction_mu
                not performed for this radiation type.
PLAT092_ALERT_4_G Check: Wavelength Given is not Cu,Ga,Mo,Ag,In Ka    0.62000 Ang.
PLAT154_ALERT_1_G The s.u.'s on the Cell Angles are Equal ..(Note)       0.03 Degree
PLAT343_ALERT_2_G Unusual sp?      Angle Range in Main Residue for       C2_2 Check
PLAT720_ALERT_4_G Number of Unusual/Non-Standard Labels ..........         89 Note
PLAT794_ALERT_5_G Tentative Bond Valency for Pt_1      (II)      .       1.87 Info
PLAT909_ALERT_3_G Percentage of I>2sig(I) Data at Theta(Max) Still        90% Note
PLAT933_ALERT_2_G Number of HKL-OMIT Records in Embedded .res File         43 Note
PLAT941_ALERT_3_G Average HKL Measurement Multiplicity ...........        3.1 Low
PLAT965_ALERT_2_G The SHELXL WEIGHT Optimisation has not Converged     Please Check
PLAT978_ALERT_2_G Number C-C Bonds with Positive Residual Density.          0 Info
```

0 **ALERT level A** = Most likely a serious problem - resolve or explain
0 **ALERT level B** = A potentially serious problem, consider carefully
6 **ALERT level C** = Check. Ensure it is not caused by an omission or oversight
11 **ALERT level G** = General information/check it is not something unexpected

2 ALERT type 1 CIF construction/syntax error, inconsistent or missing data
7 ALERT type 2 Indicator that the structure model may be wrong or deficient
5 ALERT type 3 Indicator that the structure quality may be low
2 ALERT type 4 Improvement, methodology, query or suggestion
1 ALERT type 5 Informative message, check

It is advisable to attempt to resolve as many as possible of the alerts in all categories. Often the minor alerts point to easily fixed oversights, errors and omissions in your CIF or refinement strategy, so attention to these fine details can be worthwhile. In order to resolve some of the more serious problems it may be necessary to carry out additional measurements or structure refinements. However, the purpose of your study may justify the reported deviations and the more serious of these should normally be commented upon in the discussion or experimental section of a paper or in the "special_details" fields of the CIF. checkCIF was carefully designed to identify outliers and unusual parameters, but every test has its limitations and alerts that are not important in a particular case may appear. Conversely, the absence of alerts does not guarantee there are no aspects of the results needing attention. It is up to the individual to critically assess their own results and, if necessary, seek expert advice.

Publication of your CIF in IUCr journals

A basic structural check has been run on your CIF. These basic checks will be run on all CIFs submitted for publication in IUCr journals (*Acta Crystallographica*, *Journal of Applied Crystallography*, *Journal of Synchrotron Radiation*); however, if you intend to submit to *Acta Crystallographica Section C* or *E* or *IUCrData*, you should make sure that full publication checks are run on the final version of your CIF prior to submission.

Publication of your CIF in other journals

Please refer to the *Notes for Authors* of the relevant journal for any special instructions relating to CIF submission.

PLATON version of 06/07/2023; check.def file version of 30/06/2023

Datablock NC16 - ellipsoid plot

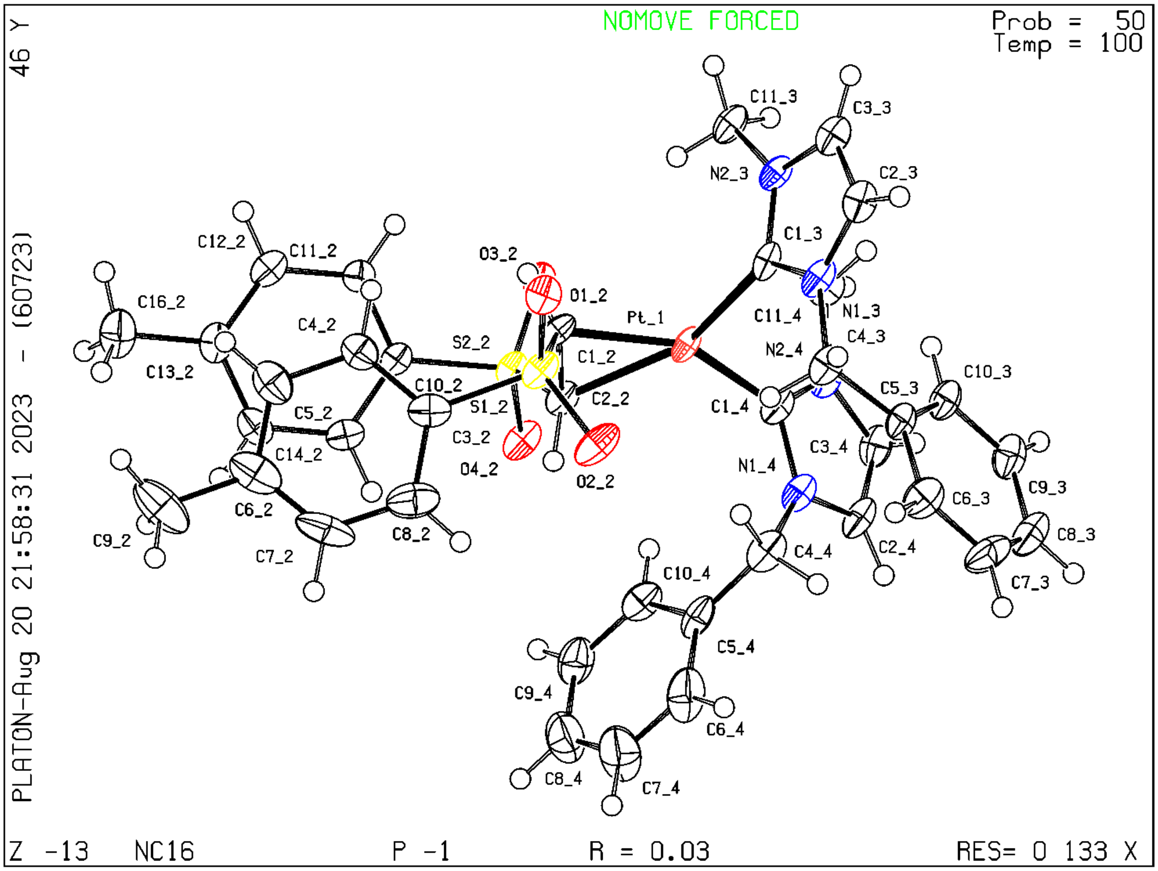

Supplement: Supplementary file 1 [file molecules-29-01119-s001.zip › 6b_checkcif.pdf]
