# Supplementary material for: Platinum(0)-η2-1,2-(E)ditosylethene Complexes Bearing Phosphine, Isocyanide and N-Heterocyclic Carbene Ligands: Synthesis and Cytotoxicity towards Ovarian and Breast Cancer Cells
Source: Molecules. 2024 Mar 1;29(5):1119. doi: 10.3390/molecules29051119 (PMC10934098; doi:10.3390/molecules29051119)

Supplementary Information

Platinum(0)-η²-1,2-(*E*)ditosylethene complexes bearing phosphine, isocyanide and *N*-heterocyclic carbene ligands: synthesis and cytotoxicity towards ovarian and breast cancer cells

Nicola Compagno[1], Rachele Piccolo[1], Enrica Bortolamiol[1], Nicola Demitri[2], Flavio Rizzolio[1,3], Fabiano Visentin[1]* and Thomas Scattolin[4]*

[1] Department of Molecular Sciences and Nanosystems Università Ca' Foscari Campus Scientifico Via Torino 155, 30174, Venezia, Italy: nicola.compagno@unive.it (N.C.); enrica.bortolamiol@unive.it (E.B.); flavio.rizzolio@unive.it (F.R.); fvise@unive.it (F.V.)

[2] Area Science Park Elettra-Sincrotrone Trieste, S.S. 14 Km 163.5 Basovizza, 34149, Trieste, Italy

[3] Pathology Unit, Department of Molecular Biology and Translational Research Centro di Riferimento Oncologico di Aviano (CRO) IRCCS via Franco Gallini 2, 33081, Aviano, Italy: flavio.rizzolio@unive.it (F.R.)

[4] Dipartimento di Scienze Chimiche, Università degli Studi di Padova, via Marzolo 1, 35131 Padova, Italy: thomas.scattolin@unipd.it (T.S)

* Correspondence: thomas.scattolin@unipd.it (T.S); fvise@unive.it (F.V.)

Table of content

NMR and IR spectra 2

X-ray diffraction analysis - Structural characterization 27

NMR and IR spectra

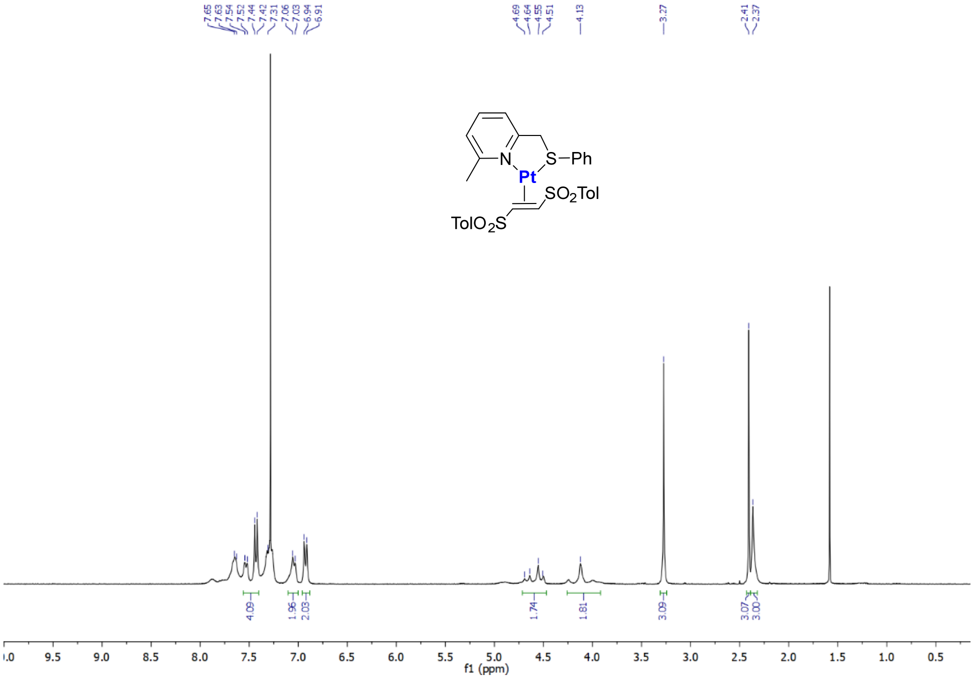

*Figure S1. ¹H NMR spectrum of compound **1** in CDCl₃ at 298K*

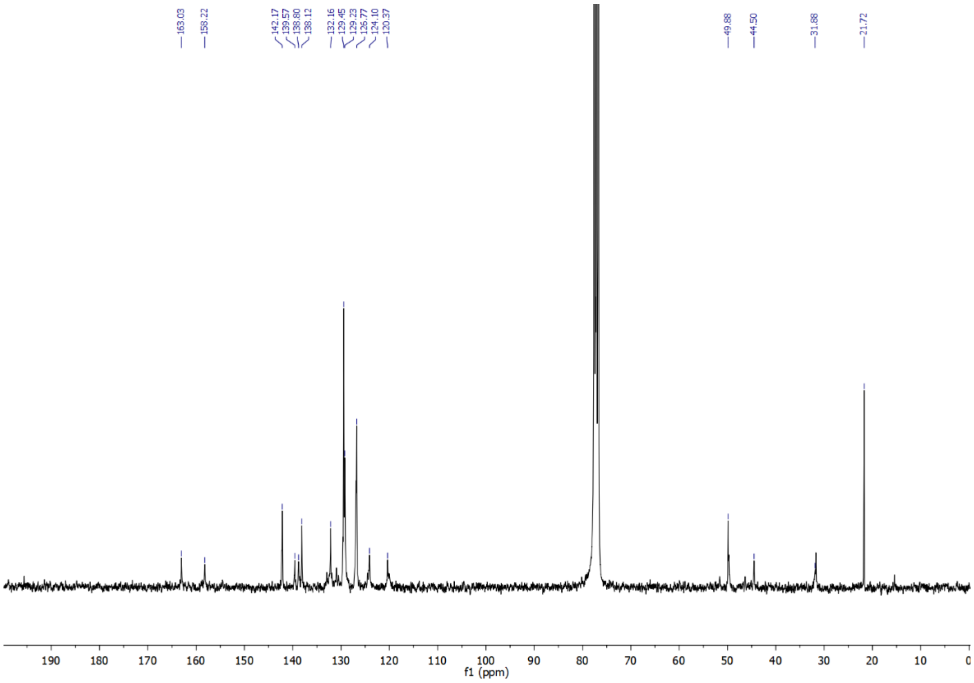

*Figure S2. ¹³C{¹H} NMR spectrum of compound **1** in CDCl₃ at 298K*

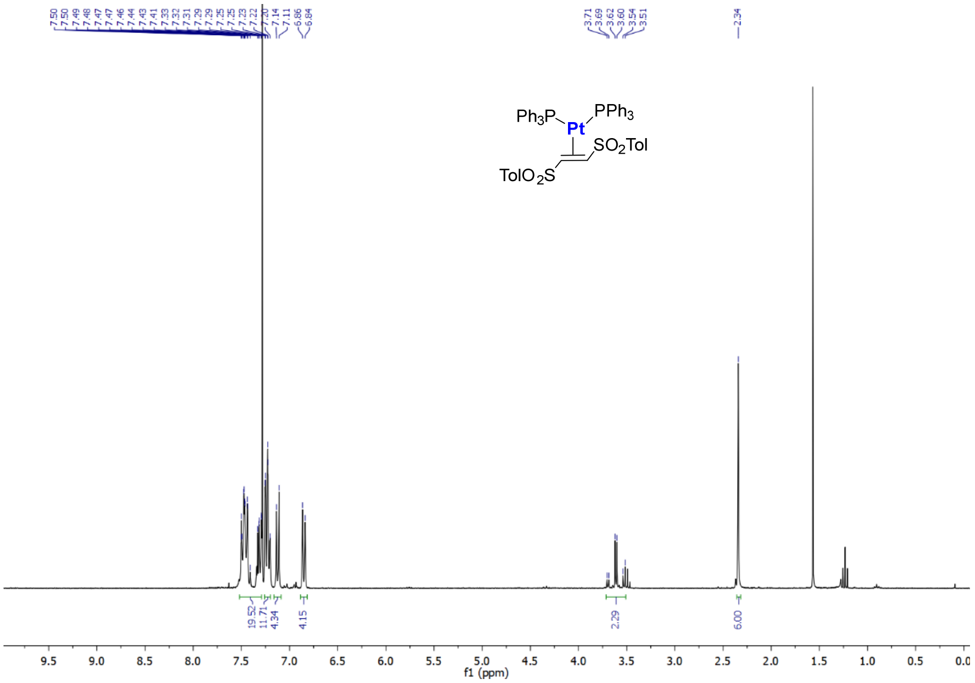

Figure S3. ¹*H NMR spectrum of compound* ***2a*** *in CDCl*₃ *at 298K*

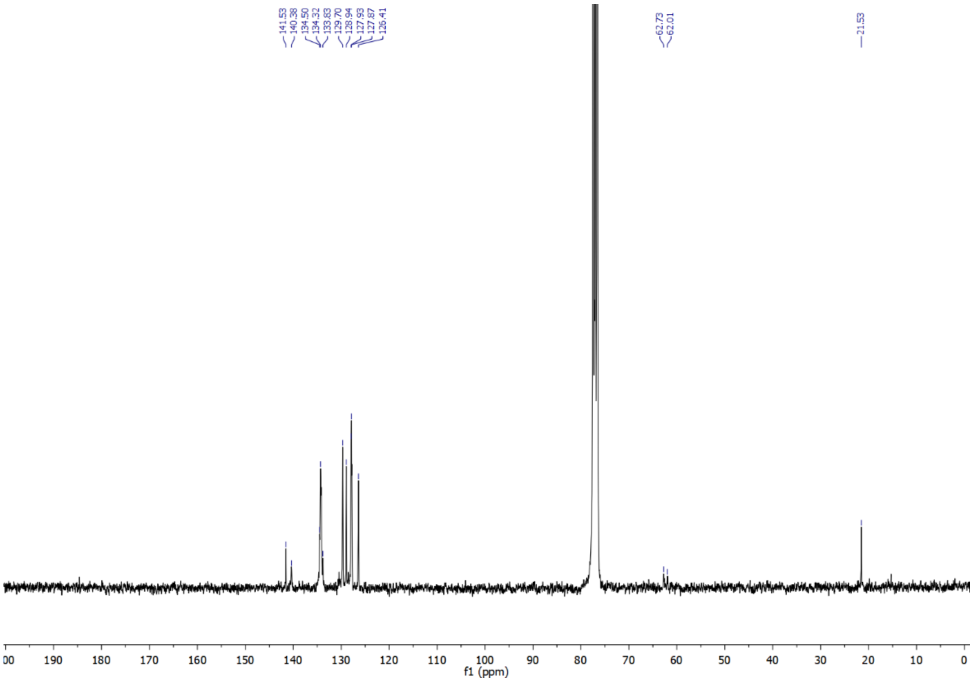

Figure S4. ¹³*C{*¹*H} NMR spectrum of compound* ***2a*** *in CDCl*₃ *at 298K*

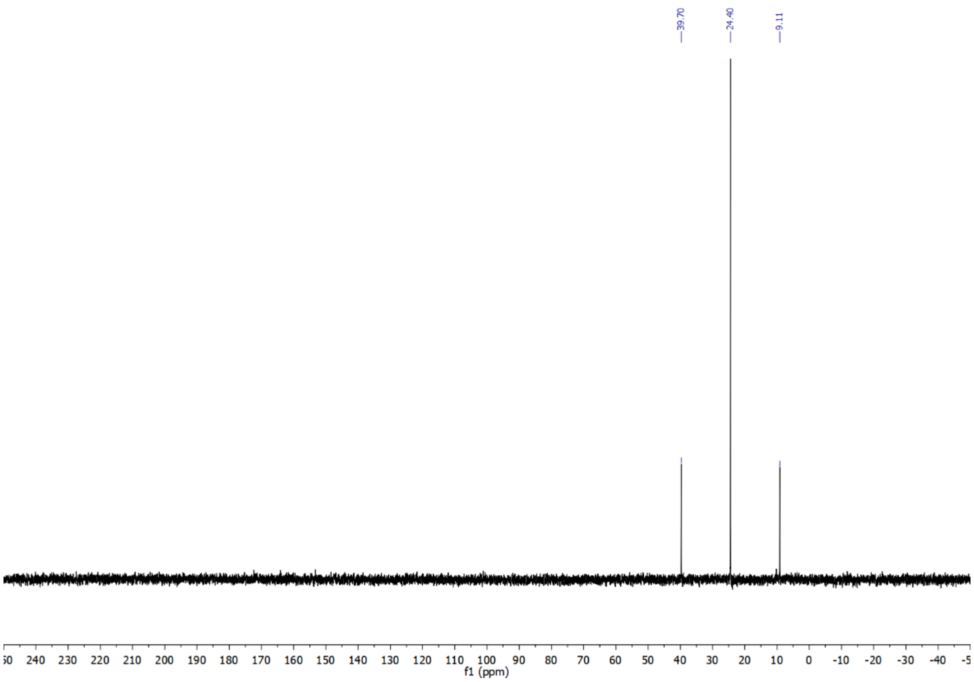

*Figure S5. $^{31}P\{^{1}H\}$ NMR spectrum of compound **2a** in $CDCl_3$ at 298K*

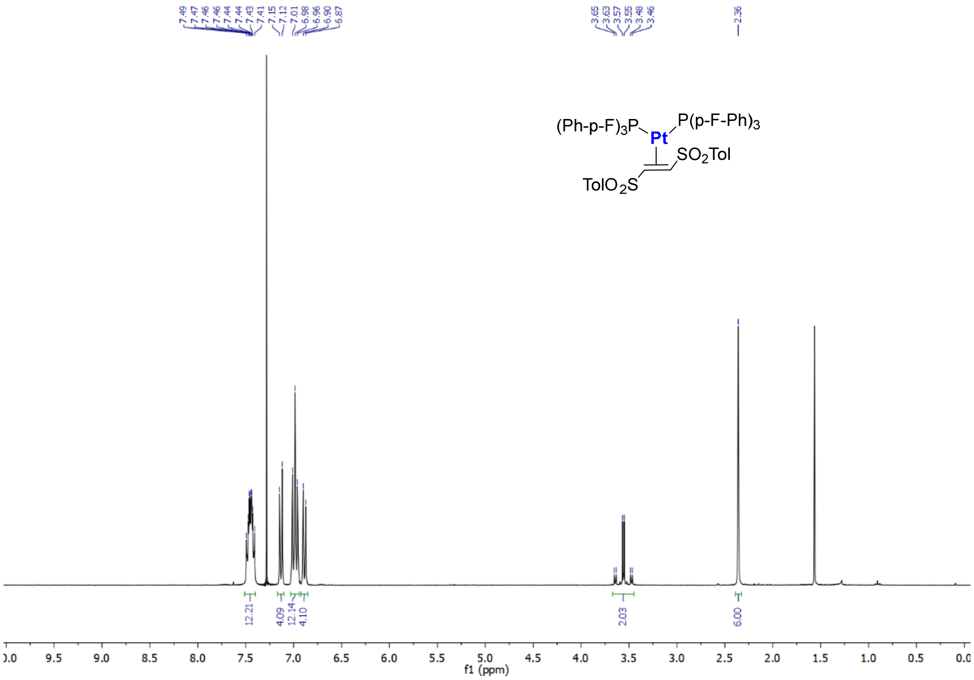

*Figure S6. ^{1}H NMR spectrum of compound **2b** in $CDCl_3$ at 298K*

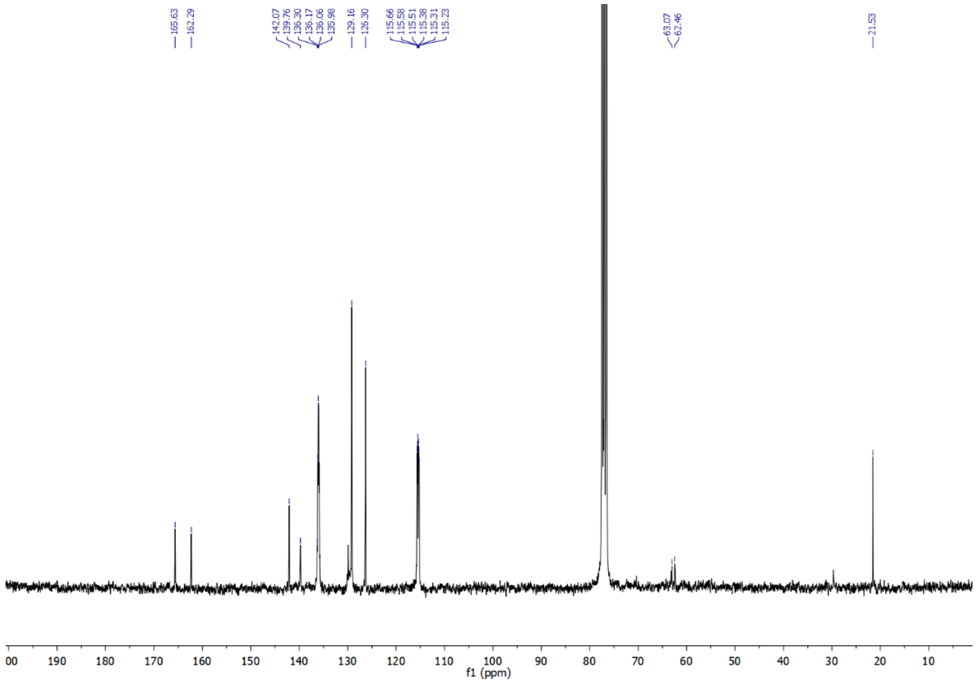

*Figure S7. $^{13}C\{^1H\}$ NMR spectrum of compound **2b** in $CDCl_3$ at 298K*

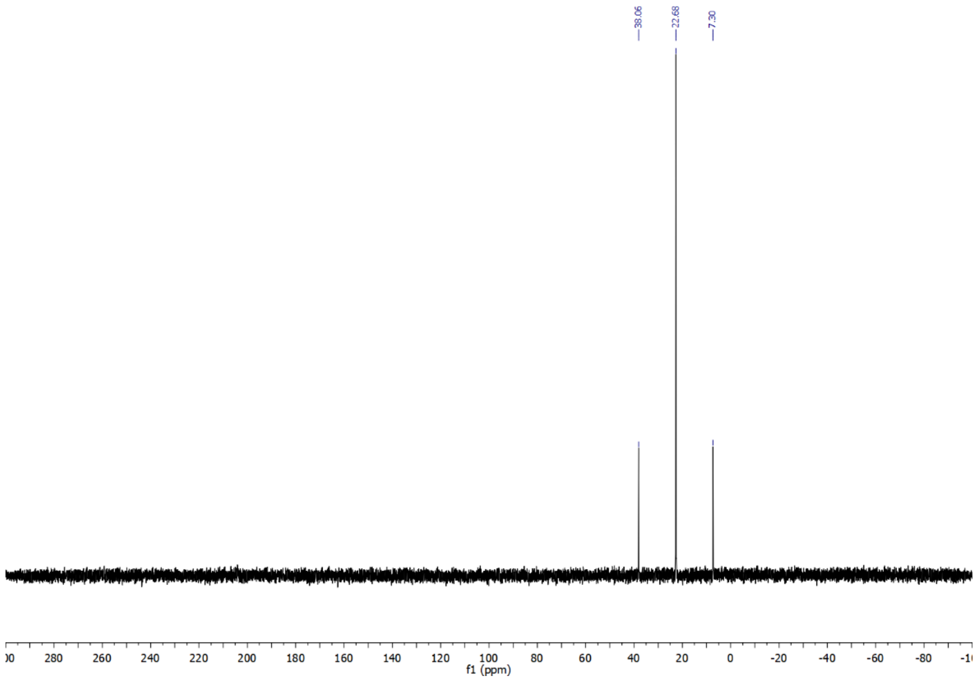

*Figure S8. $^{31}P\{^1H\}$ NMR spectrum of compound **2b** in $CDCl_3$ at 298K*

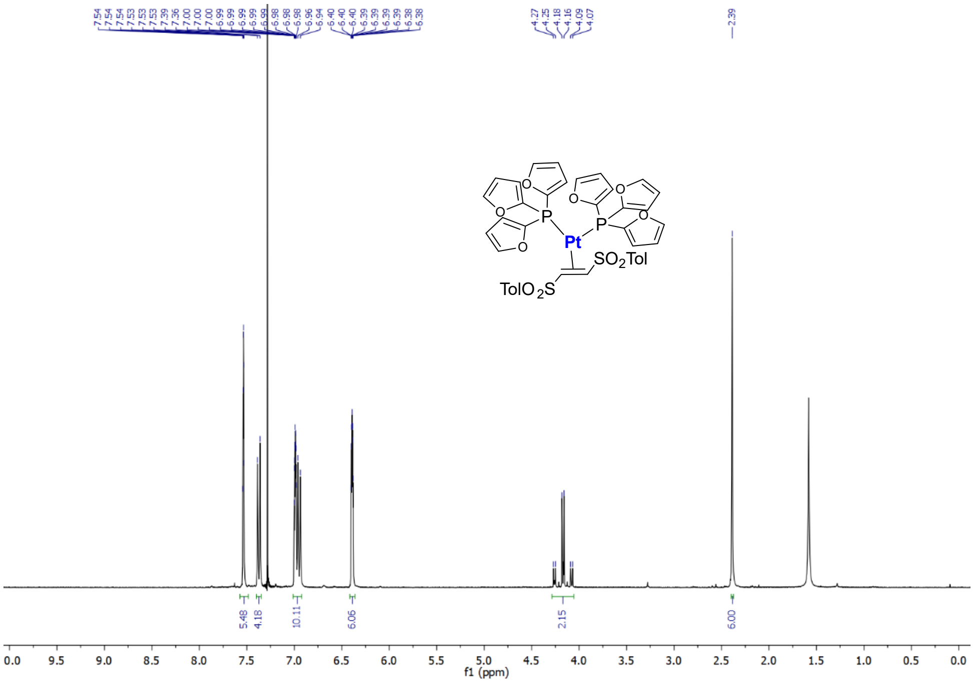

Figure S9. ¹H NMR spectrum of compound ***2c*** *in CDCl₃ at 298K*

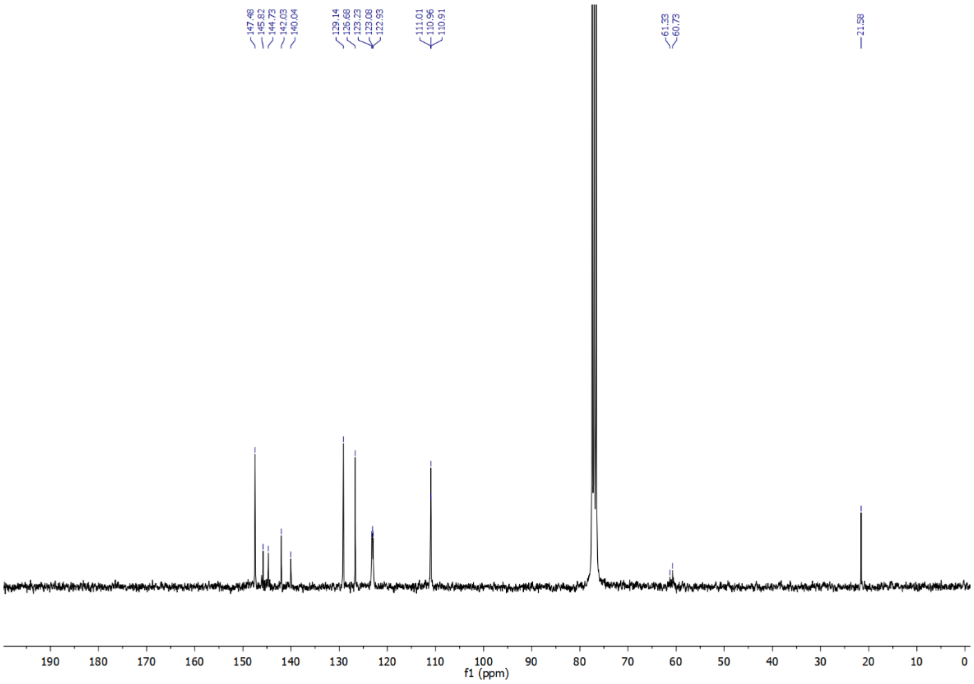

Figure S10. ¹³C{¹H} NMR spectrum of compound ***2c*** *in CDCl₃ at 298K*

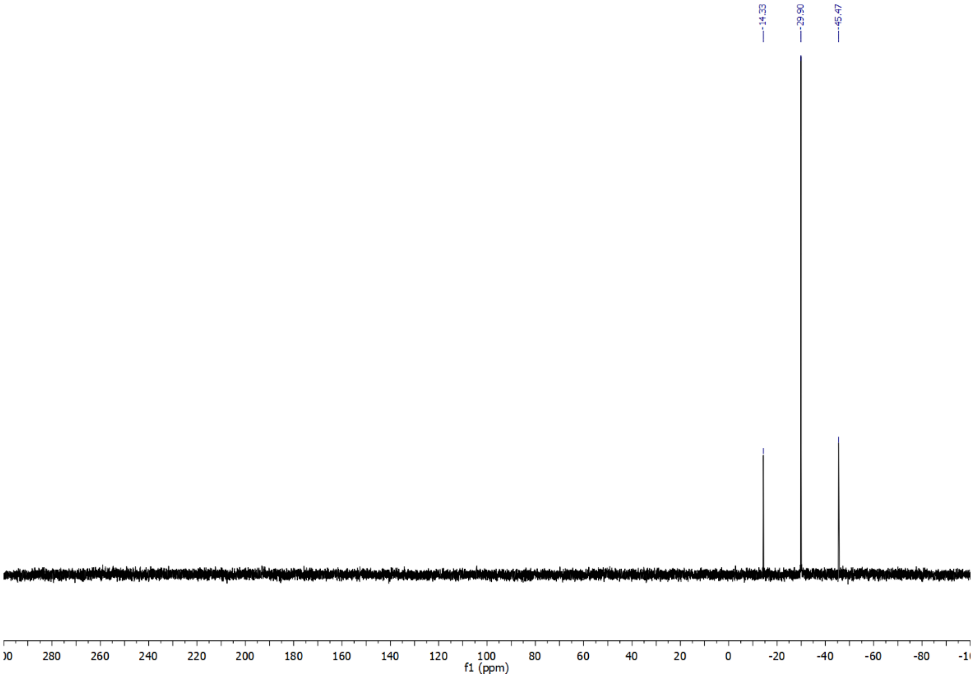

Figure S11. $^{31}P\{^{1}H\}$ *NMR spectrum of compound* ***2c*** *in* $CDCl_3$ *at 298K*

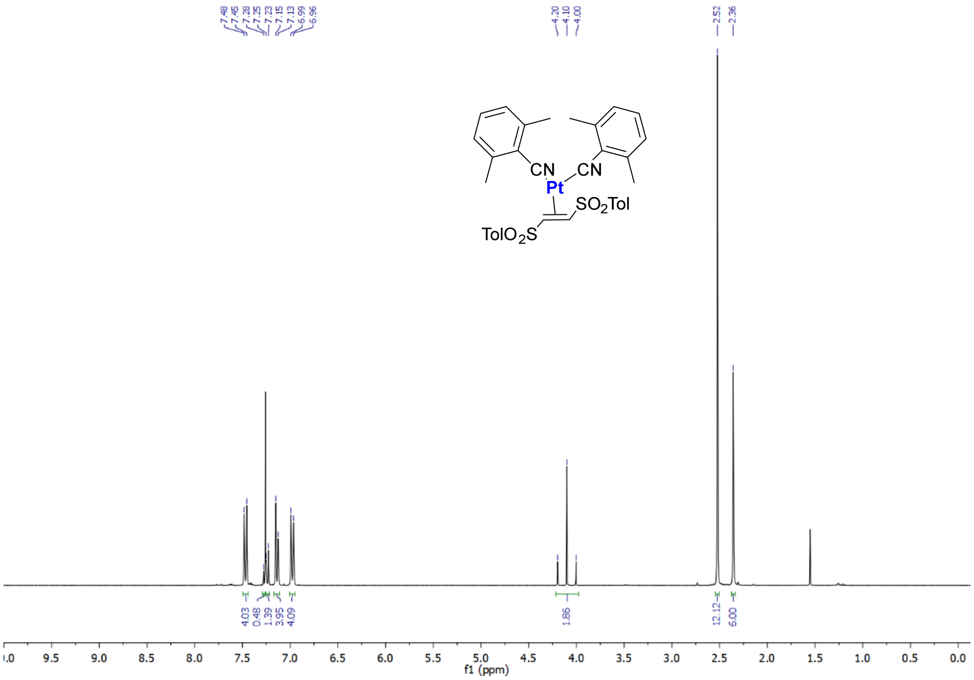

Figure S12. ^{1}H *NMR spectrum of compound* ***3a*** *in* $CDCl_3$ *at 298K*

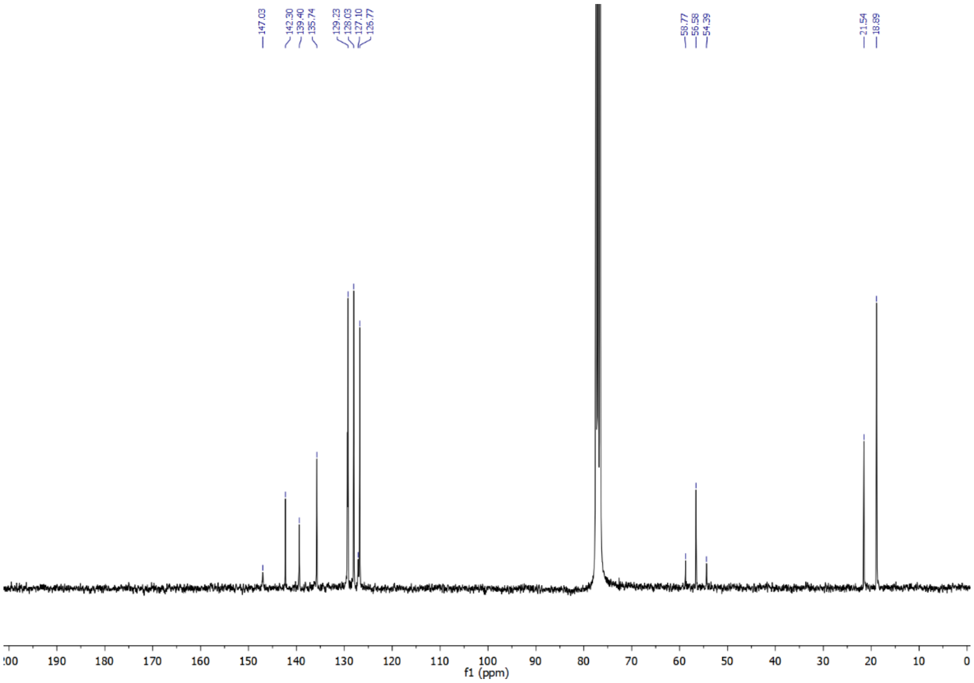

Figure S13. $^{13}C\{^1H\}$ *NMR spectrum of compound* ***3a*** *in* $CDCl_3$ *at 298K*

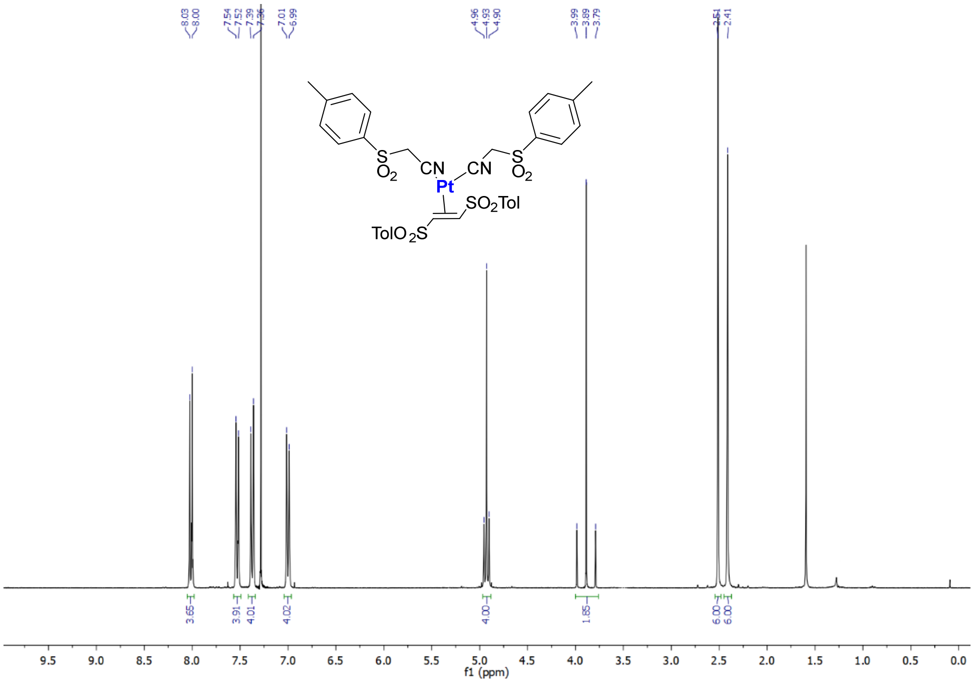

Figure S14 .^1H *NMR spectrum of compound* ***3b*** *in* $CDCl_3$ *at 298K*

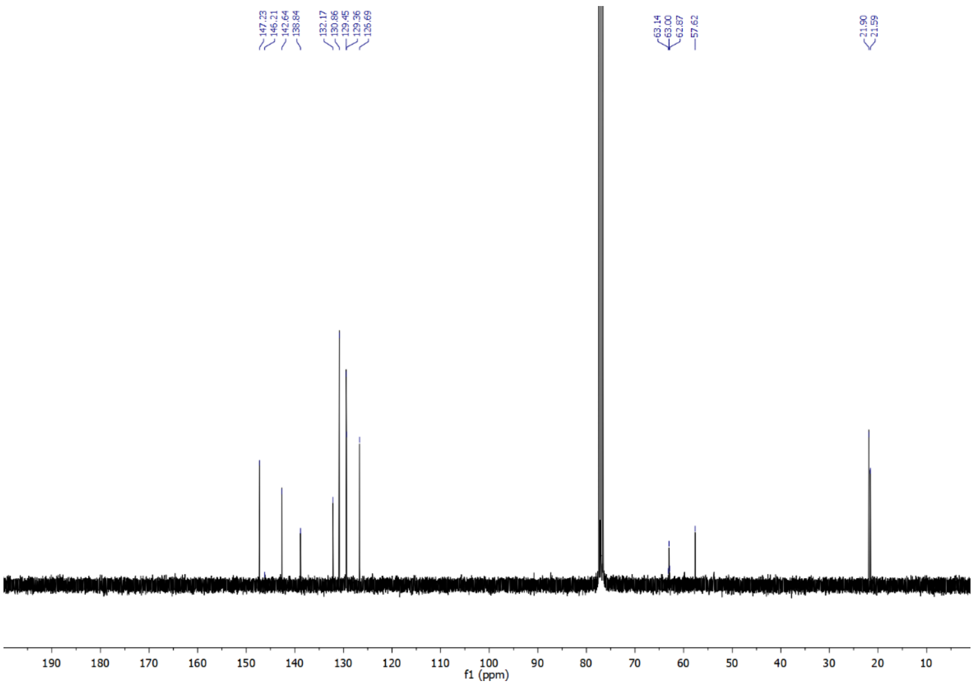

Figure S15. $^{13}C\{^{1}H\}$ *NMR spectrum of compound* ***3b*** *in* $CDCl_3$ *at 298K*

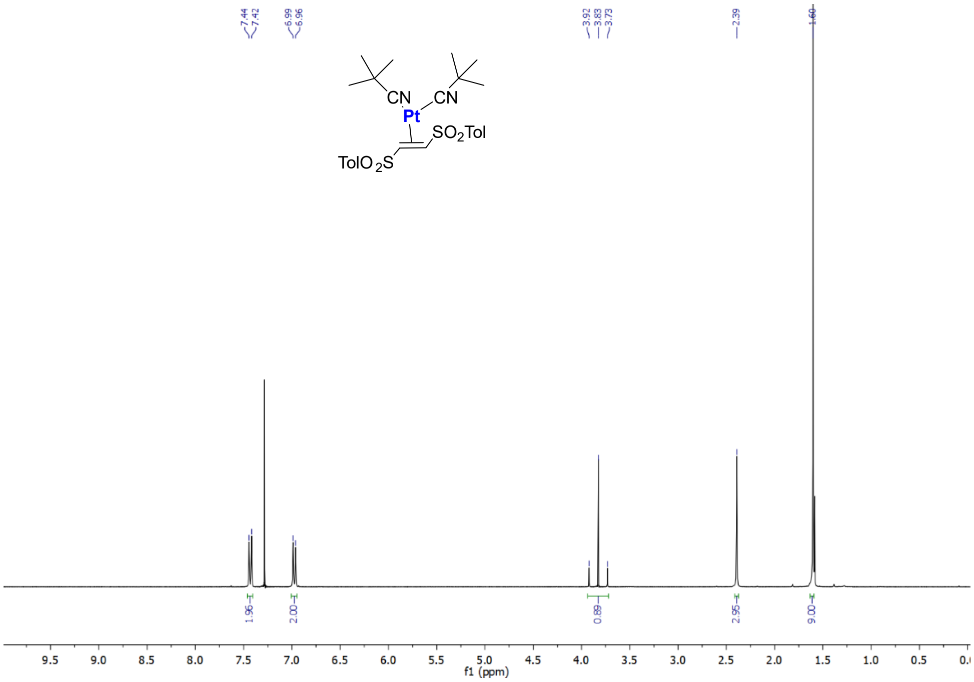

Figure S16. ^{1}H *NMR spectrum of compound* ***3c*** *in* $CDCl_3$ *at 298K*

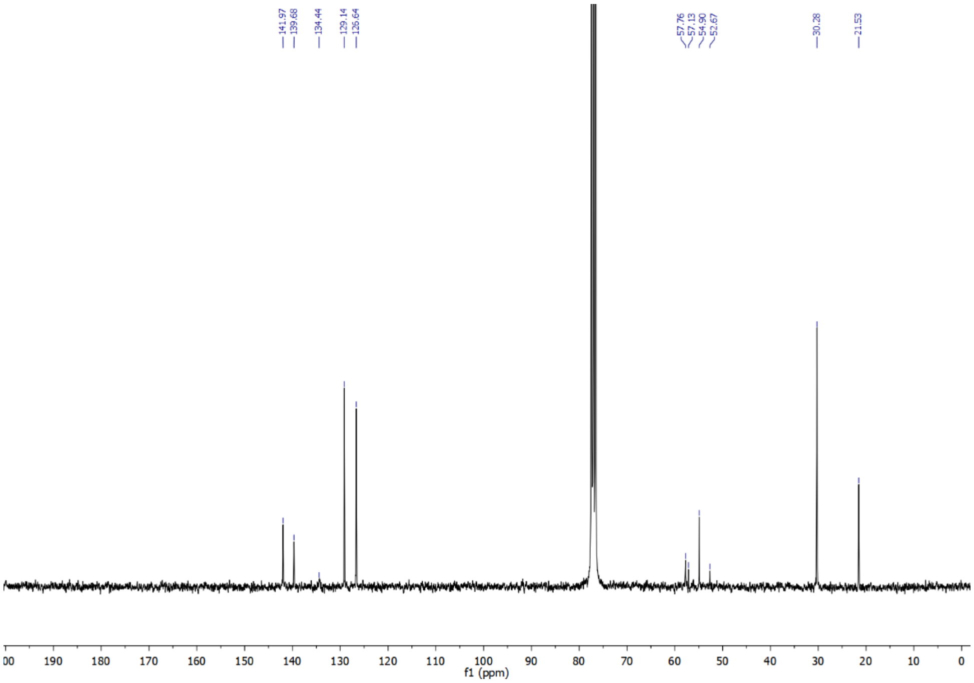

Figure S17. $^{13}C\{^{1}H\}$ *NMR spectrum of compound* ***3c*** *in* $CDCl_3$ *at 298K*

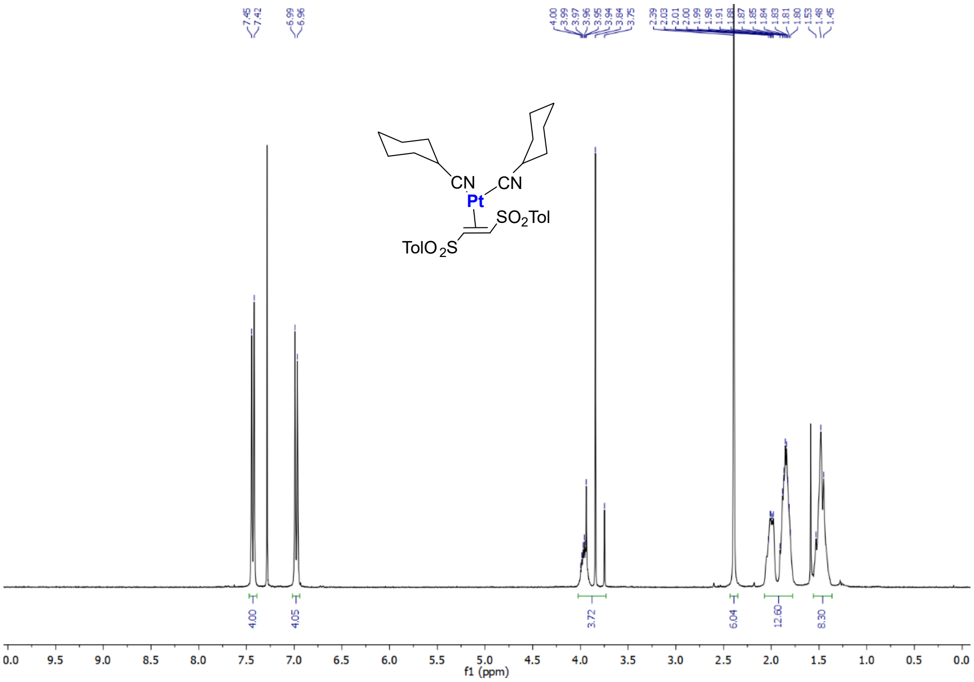

Figure S18. ^{1}H *NMR spectrum of compound* ***3d*** *in* $CDCl_3$ *at 298K*

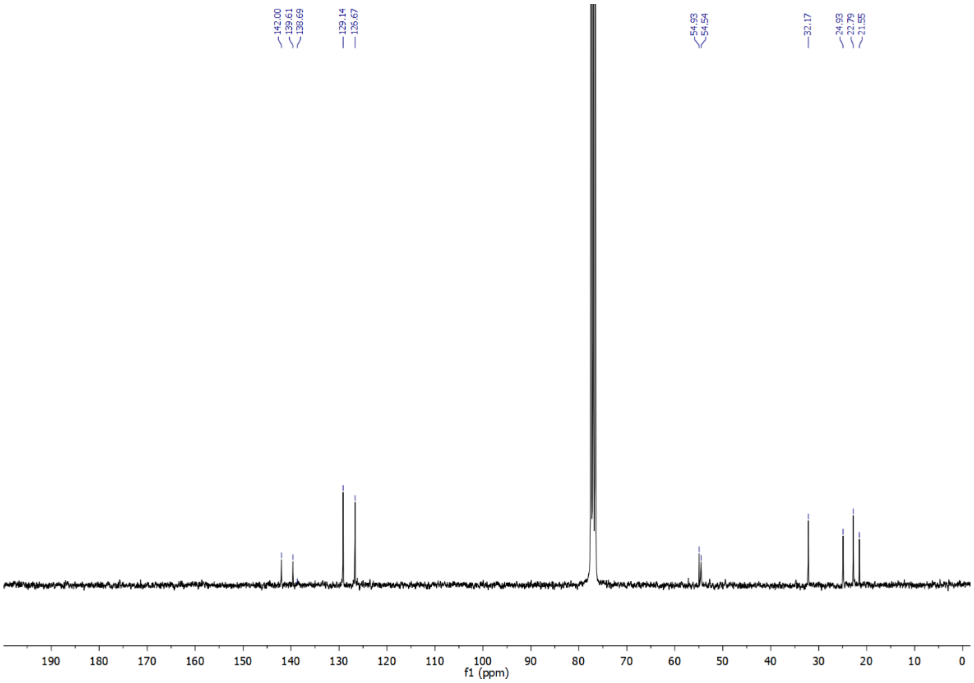

*Figure S19. ¹³C{¹H} NMR spectrum of compound **3d** in CDCl₃ at 298K*

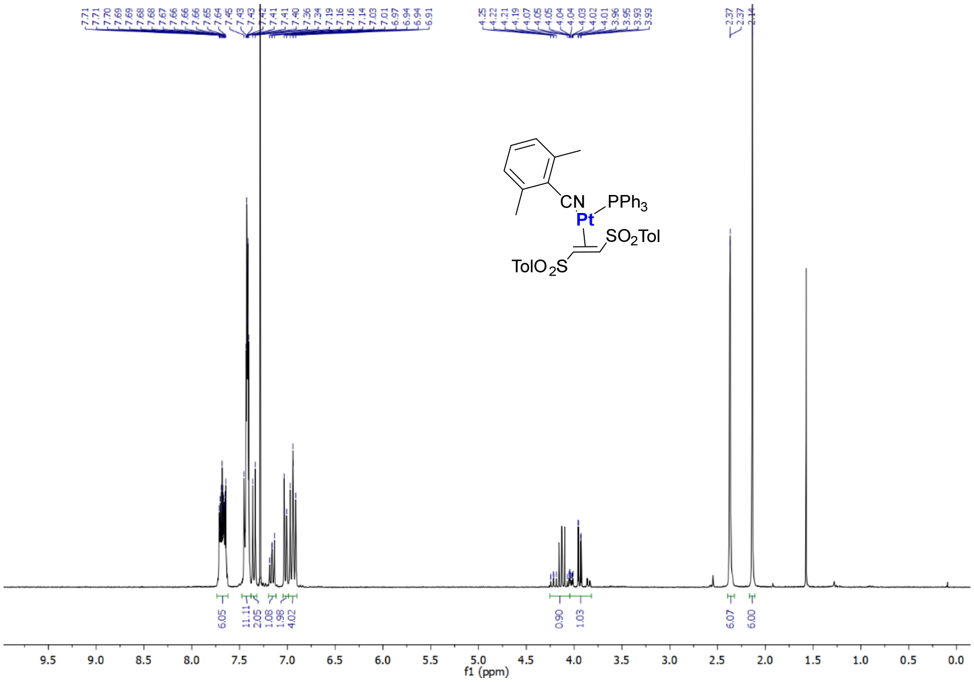

*Figure S20. ¹H NMR spectrum of compound **4a** in CDCl₃ at 298K*

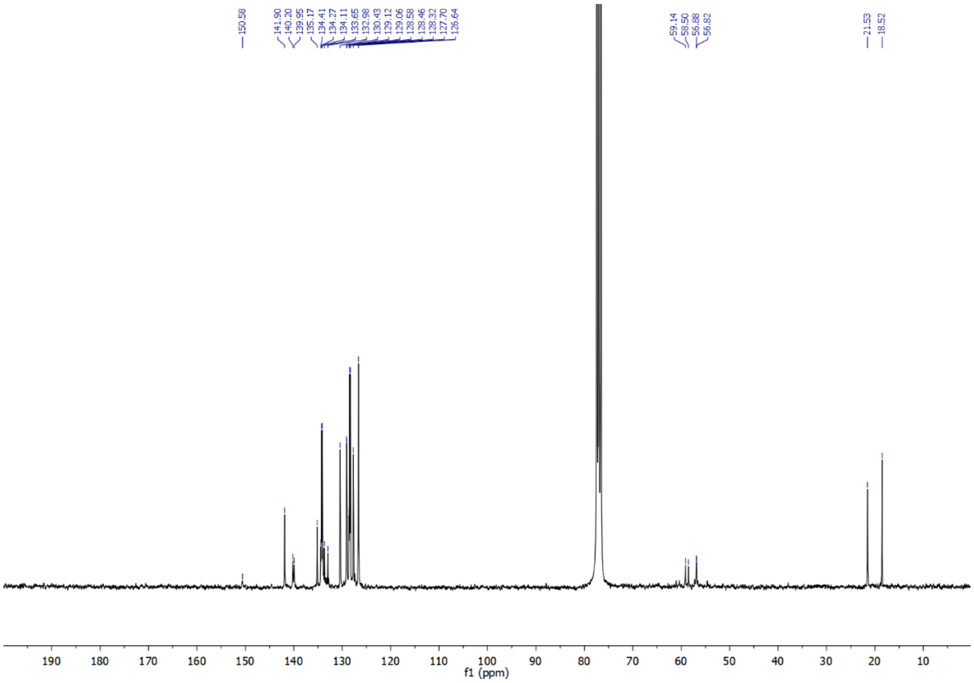

Figure S21. $^{13}C\{^1H\}$ *NMR spectrum of compound **4a** in* $CDCl_3$ *at 298K*

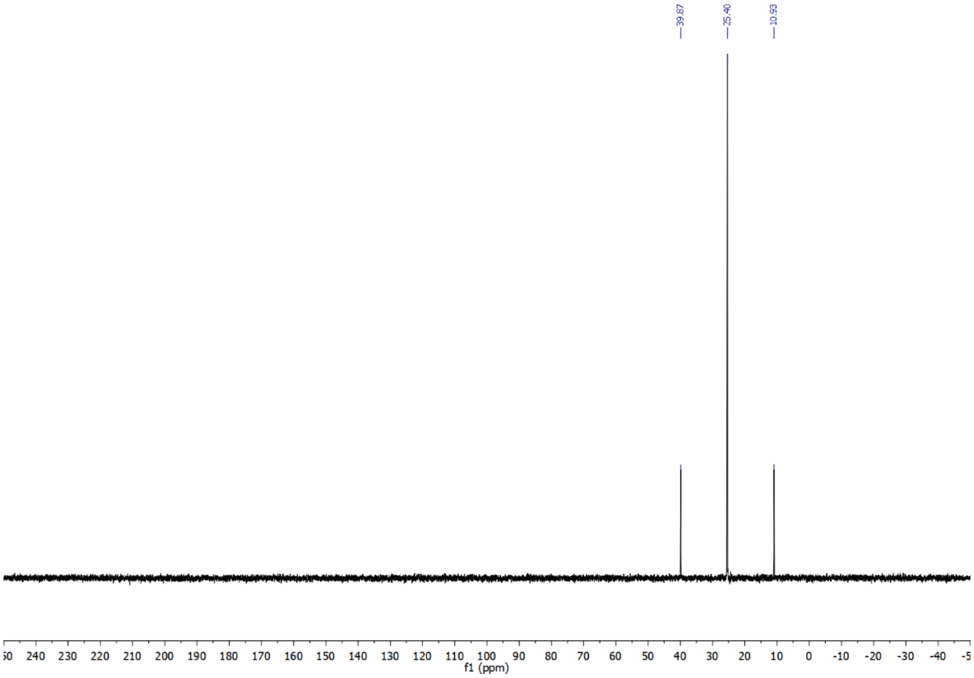

Figure S22. $^{31}P\{^1H\}$ *NMR spectrum of compound **4a** in* $CDCl_3$ *at 298K*

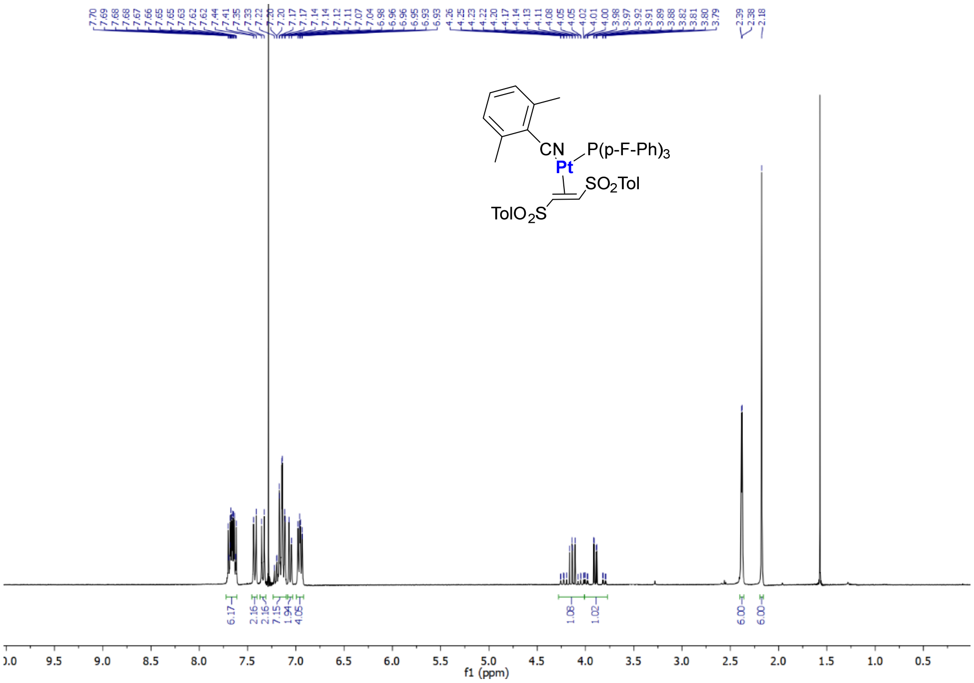

Figure S23. [1]*H NMR spectrum of compound* ***4b*** *in* $CDCl_3$ *at 298K*

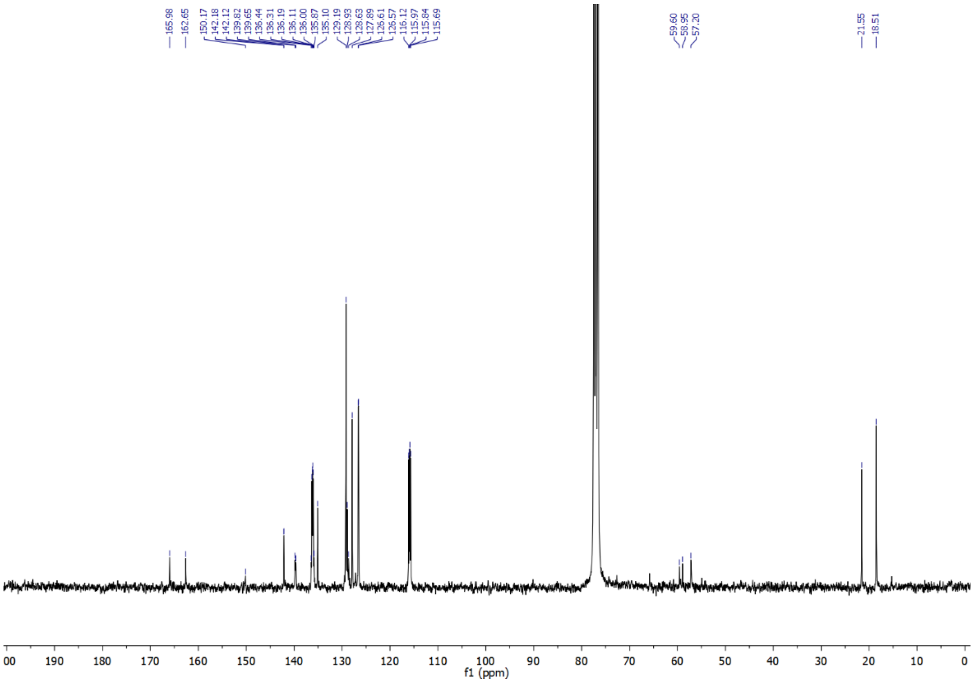

Figure S24. $^{13}C\{^{1}H\}$ *NMR spectrum of compound* ***4b*** *in* $CDCl_3$ *at 298K*

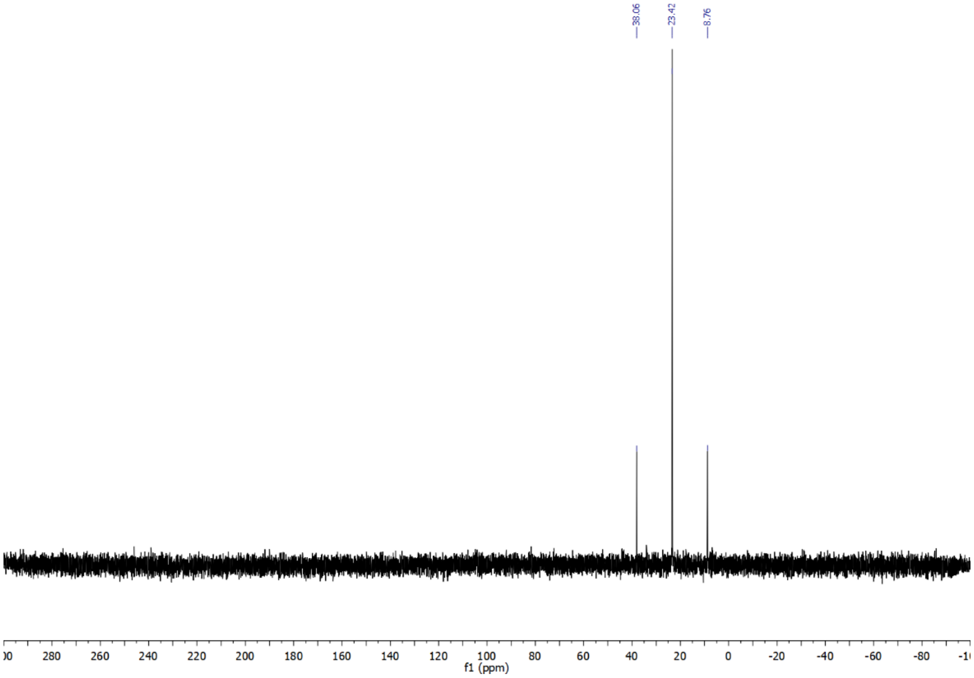

Figure S25. $^{31}P\{^{1}H\}$ *NMR spectrum of compound* ***4b*** *in* $CDCl_3$ *at 298K*

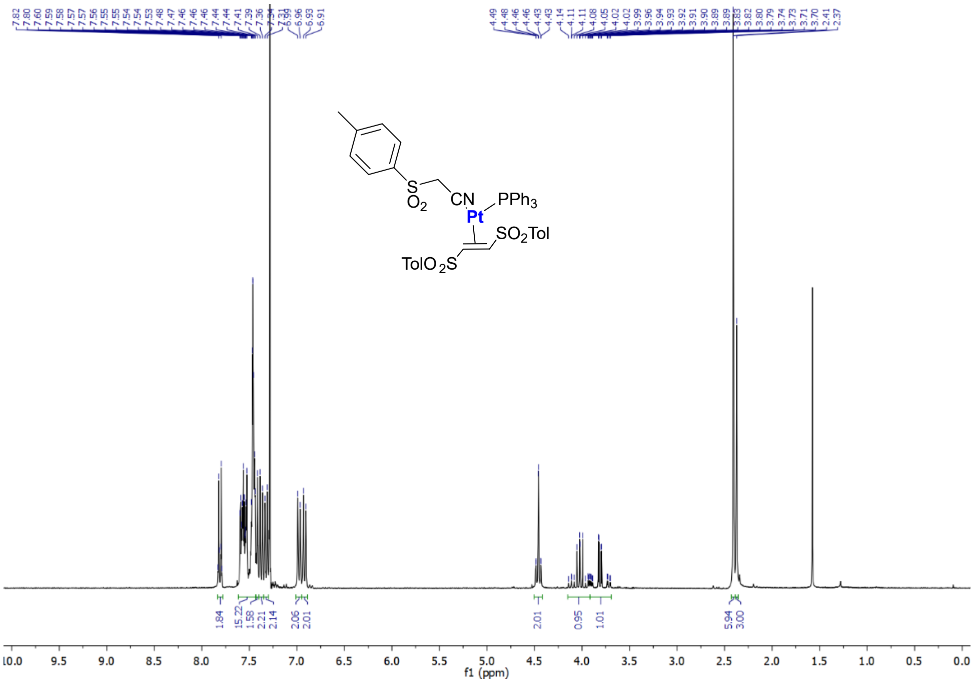

Figure S26. ^{1}H *NMR spectrum of compound* ***4c*** *in* $CDCl_3$ *at 298K*

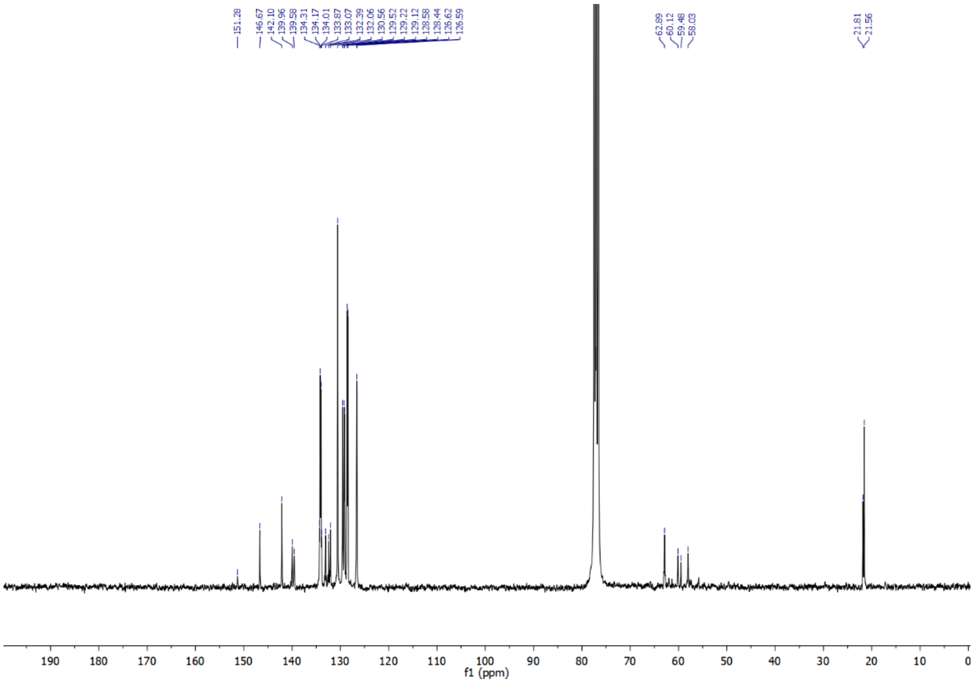

Figure S27.$^{13}C\{^1H\}$ NMR spectrum of compound **4c** in $CDCl_3$ at 298K

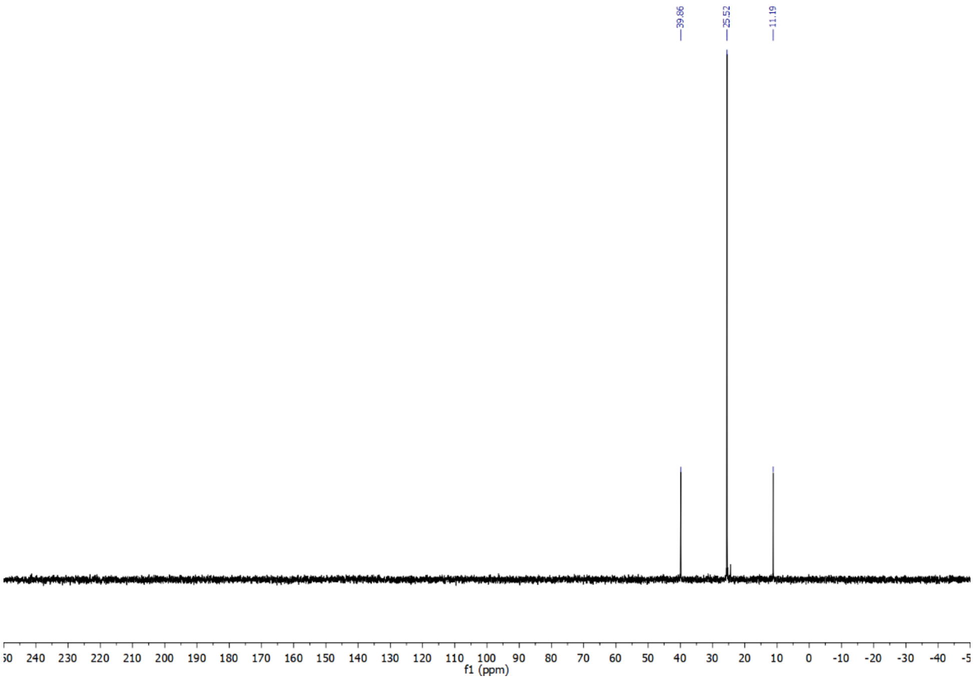

Figure S28. $^{31}P\{^1H\}$ NMR spectrum of compound 4**c** in $CDCl_3$ at 298K

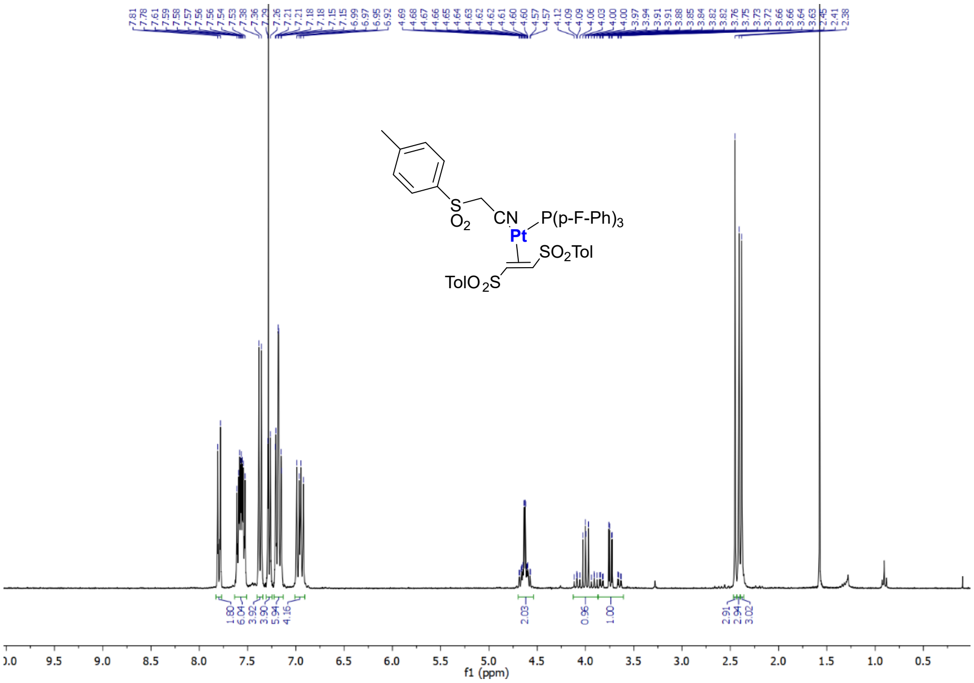

Figure S29. ^{1}H *NMR spectrum of compound* ***4d*** *in* $CDCl_3$ *at 298K*

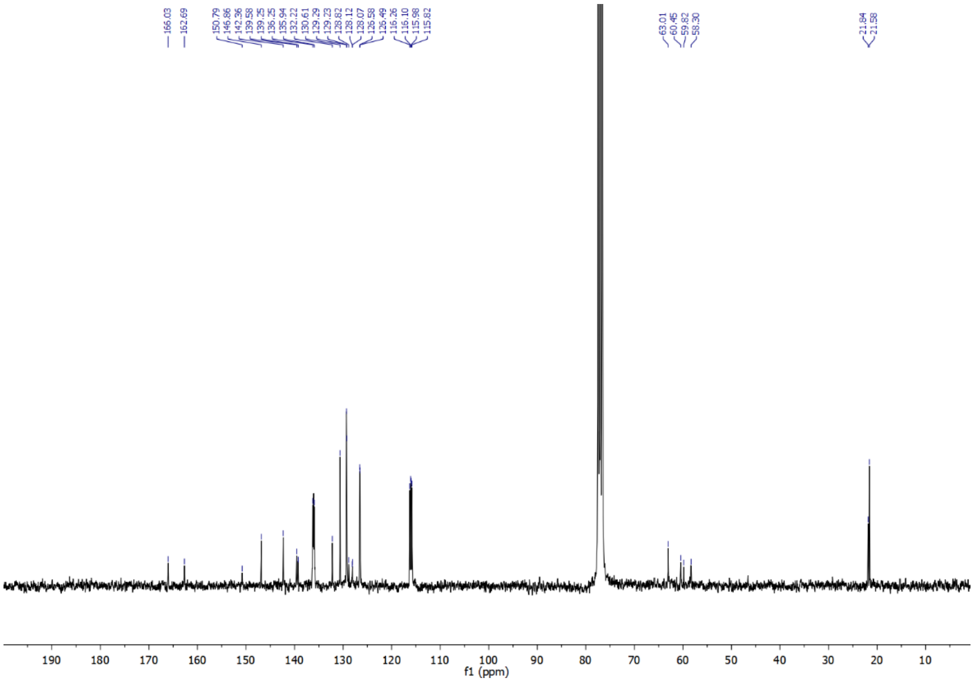

Figure S30. $^{13}C\{^{1}H\}$ *NMR spectrum of compound* ***4d*** *in* $CDCl_3$ *at 298K*

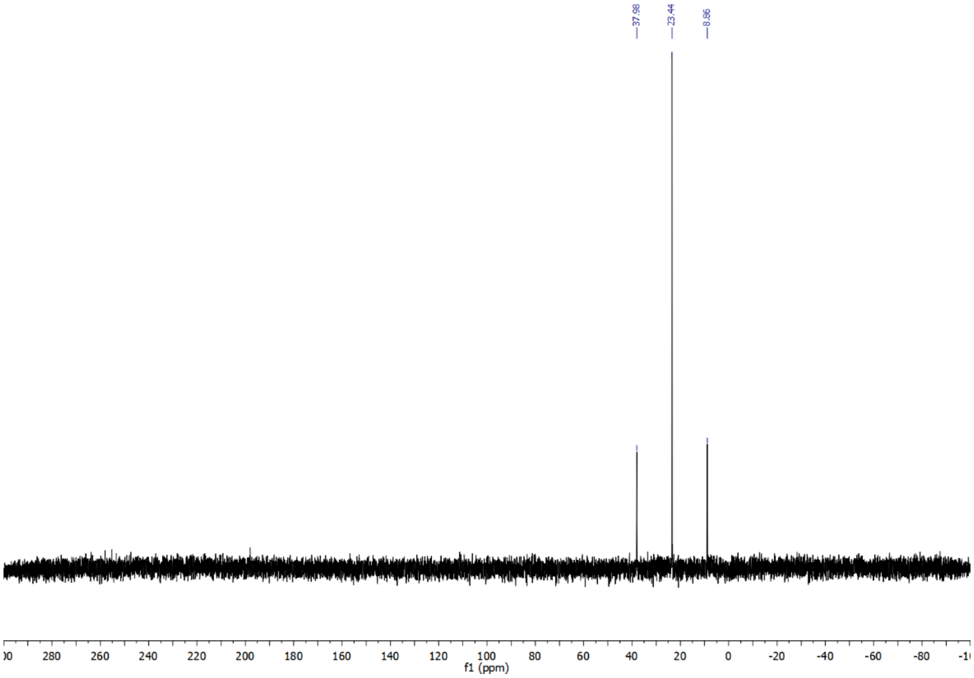

*Figure S31. ³¹P{¹H} NMR spectrum of compound **4d** in CDCl₃ at 298K*

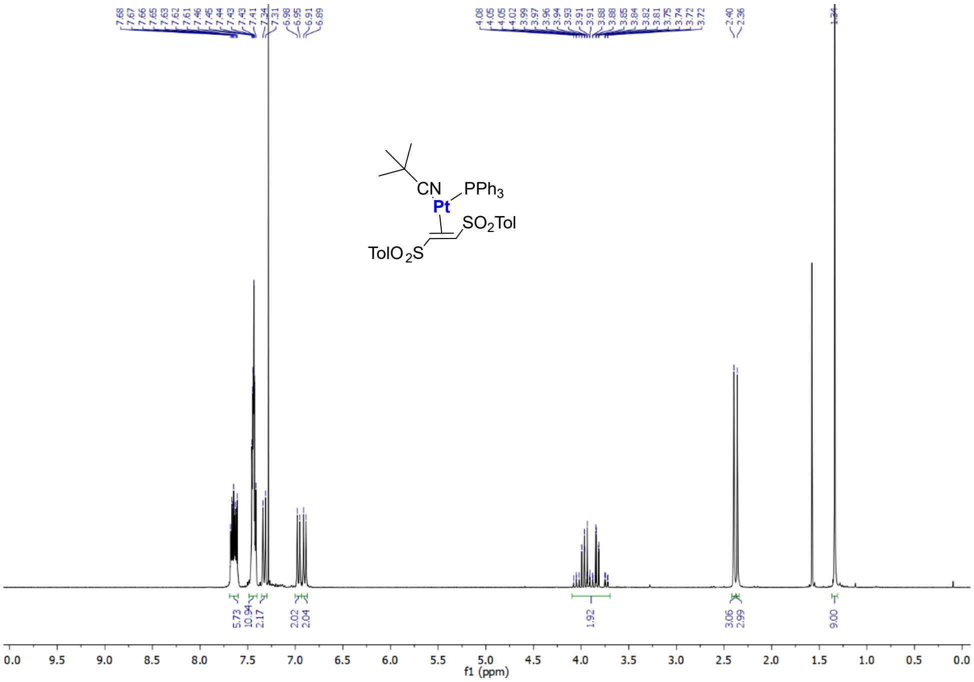

*Figure S32. ¹H NMR spectrum of compound **4e** in CDCl₃ at 298K*

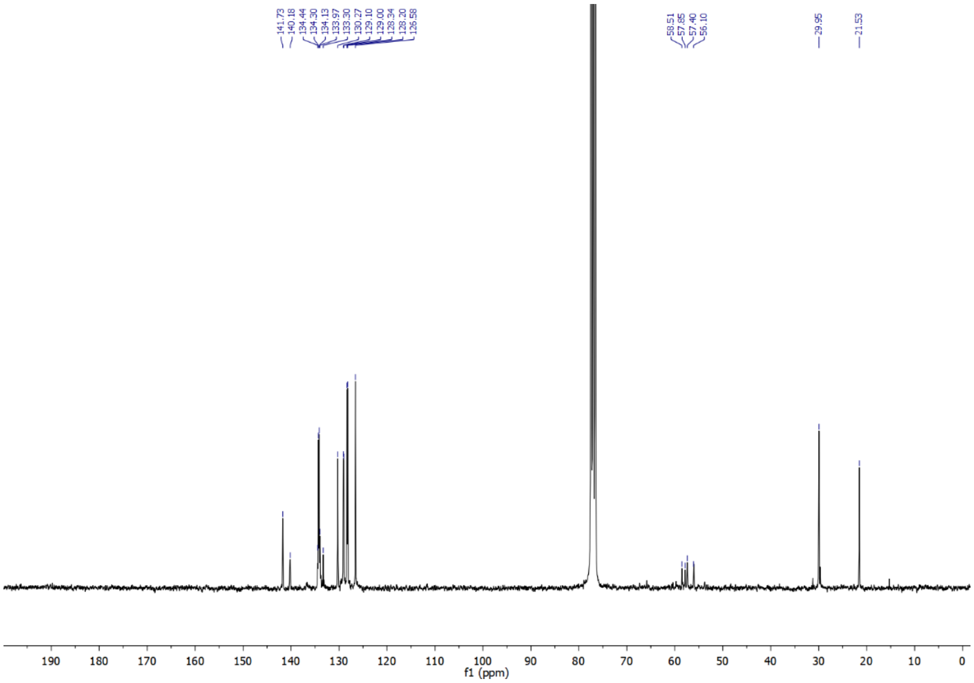

Figure S33. *$^{13}C\{^{1}H\}$ NMR spectrum of compound* ***4e*** *in $CDCl_3$ at 298K*

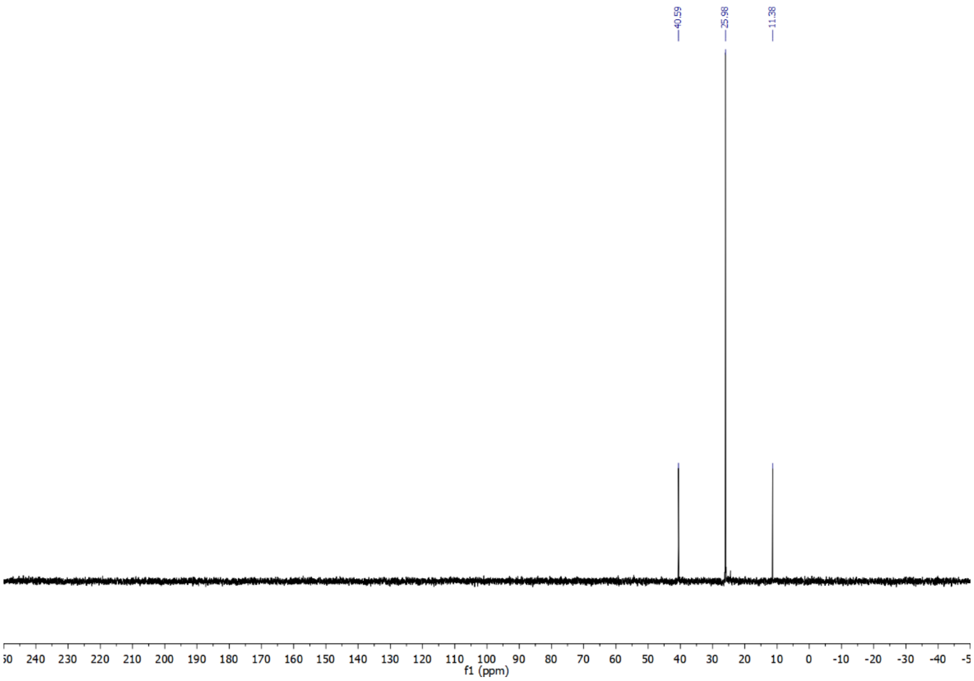

Figure S34. *$^{31}P\{^{1}H\}$ NMR spectrum of compound* ***4e*** *in $CDCl_3$ at 298K*

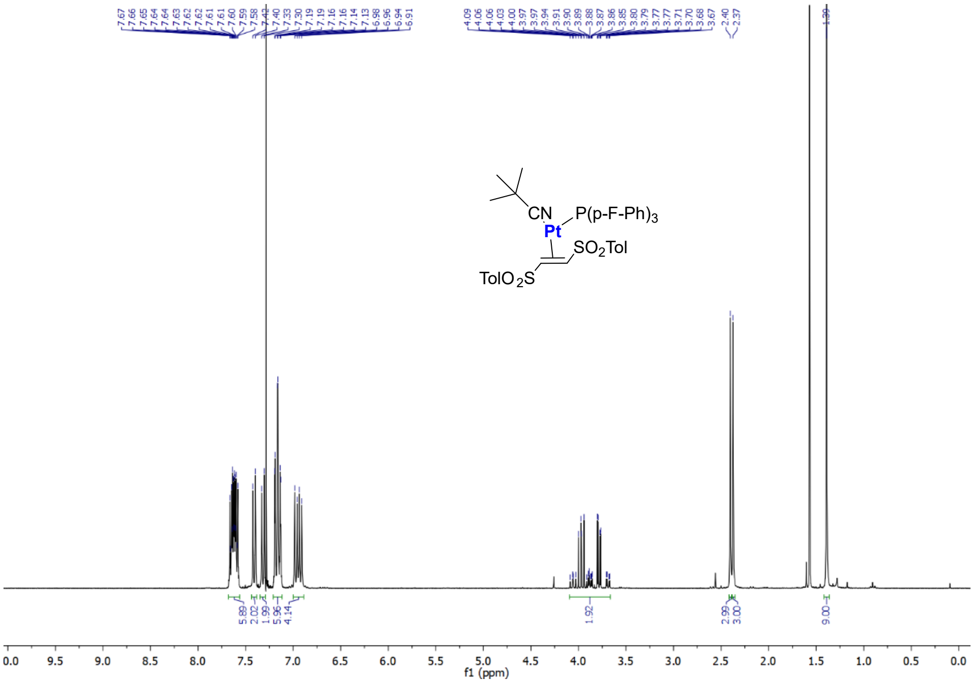

*Figure S35. ¹H NMR spectrum of compound **4f** in $CDCl_3$ at 298K*

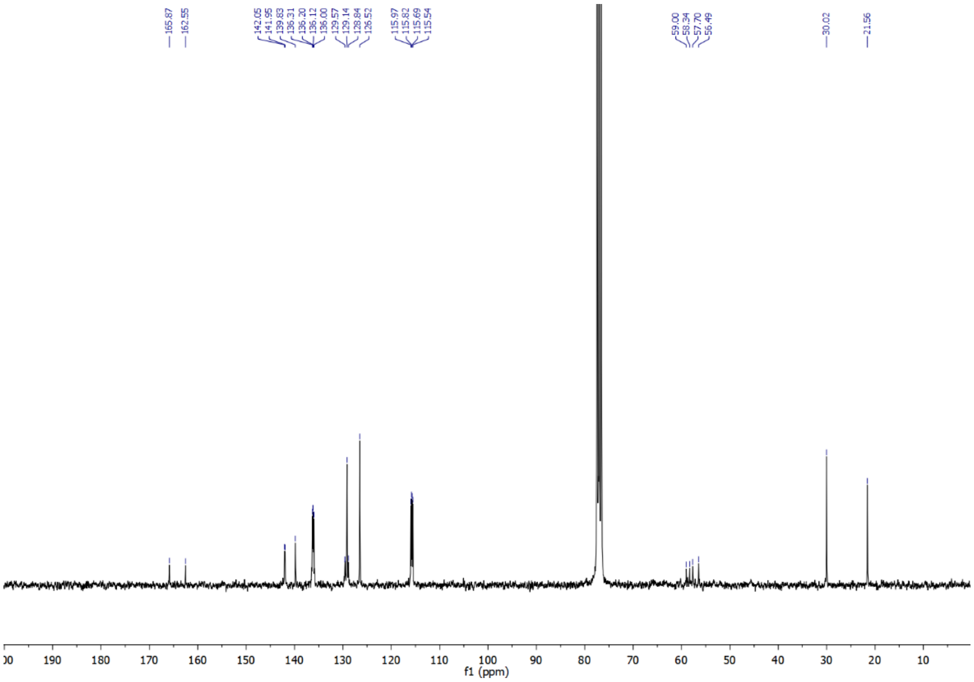

*Figure S36. $^{13}C\{^1H\}$ NMR spectrum of compound **4f** in $CDCl_3$ at 298K*

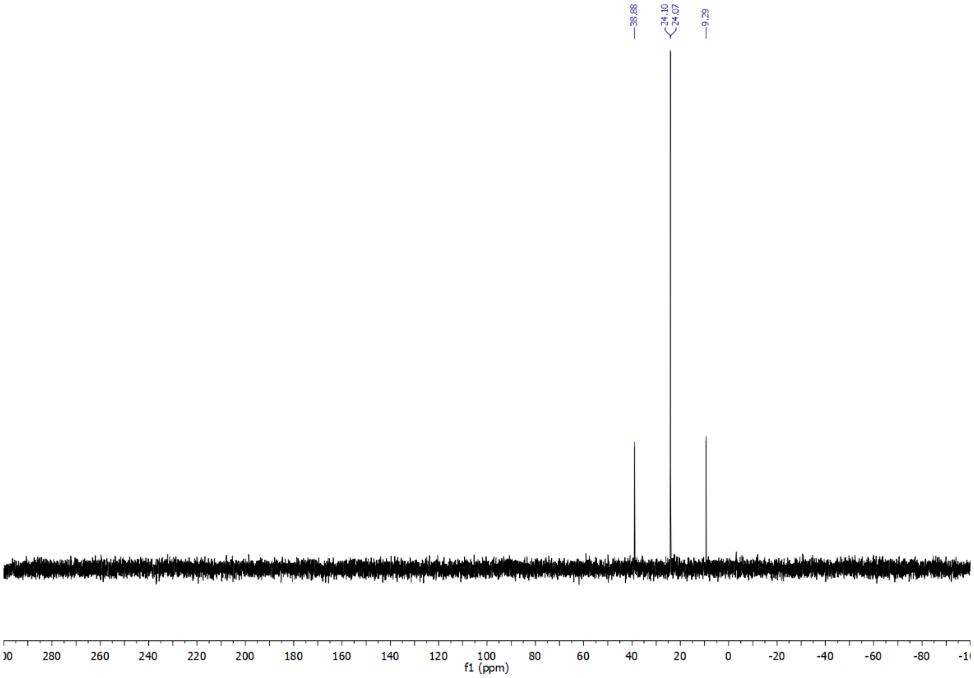

*Figure S37. $^{31}P\{^{1}H\}$ NMR spectrum of compound **4f** in $CDCl_3$ at 298K*

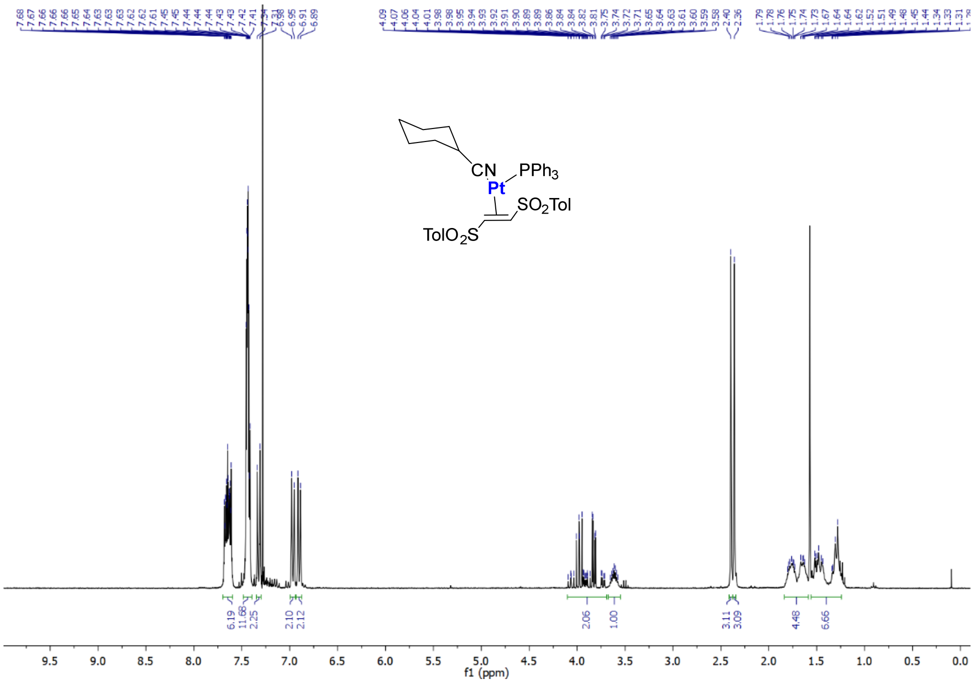

*Figure S38. ^{1}H NMR spectrum of compound **4g** in $CDCl_3$ at 298K*

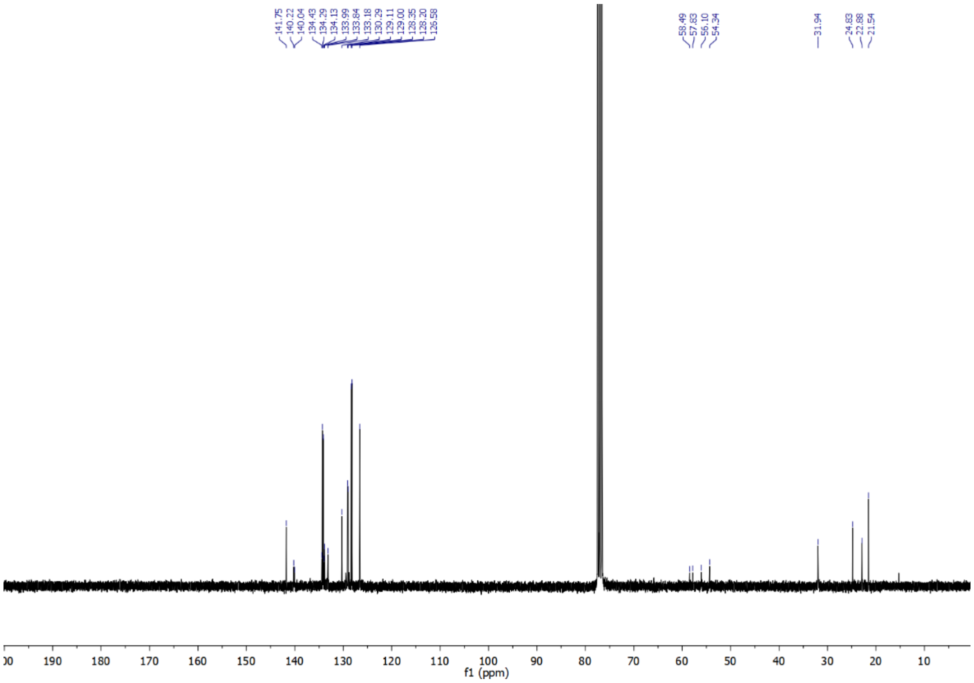

*Figure S39. $^{13}C\{^{1}H\}$ NMR spectrum of compound **4g** in $CDCl_3$ at 298K*

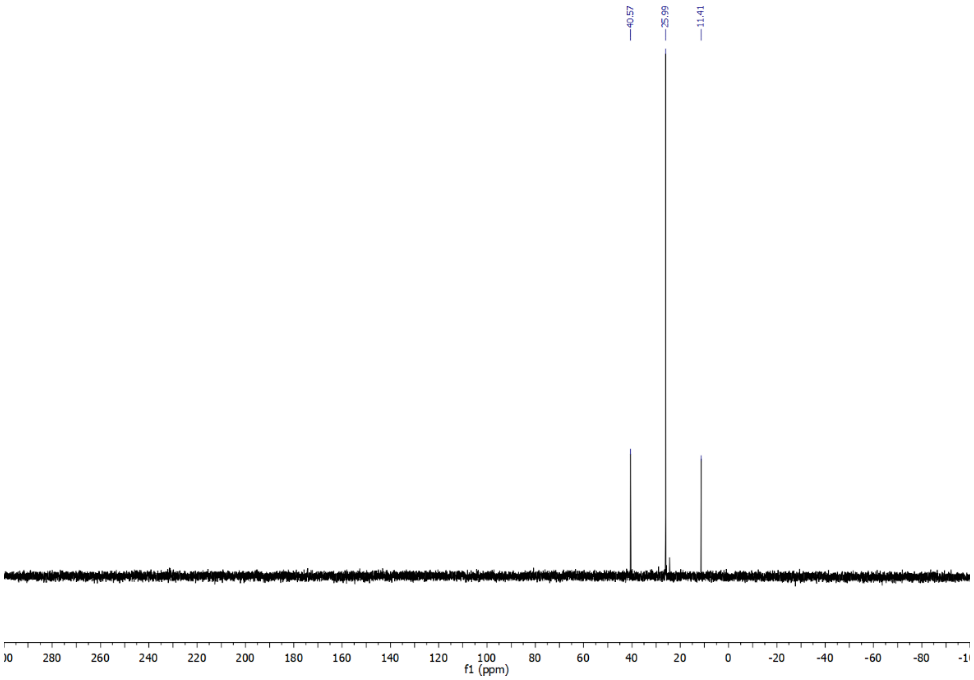

*Figure S40. $^{31}P\{^{1}H\}$ NMR spectrum of compound **4g** in $CDCl_3$ at 298K*

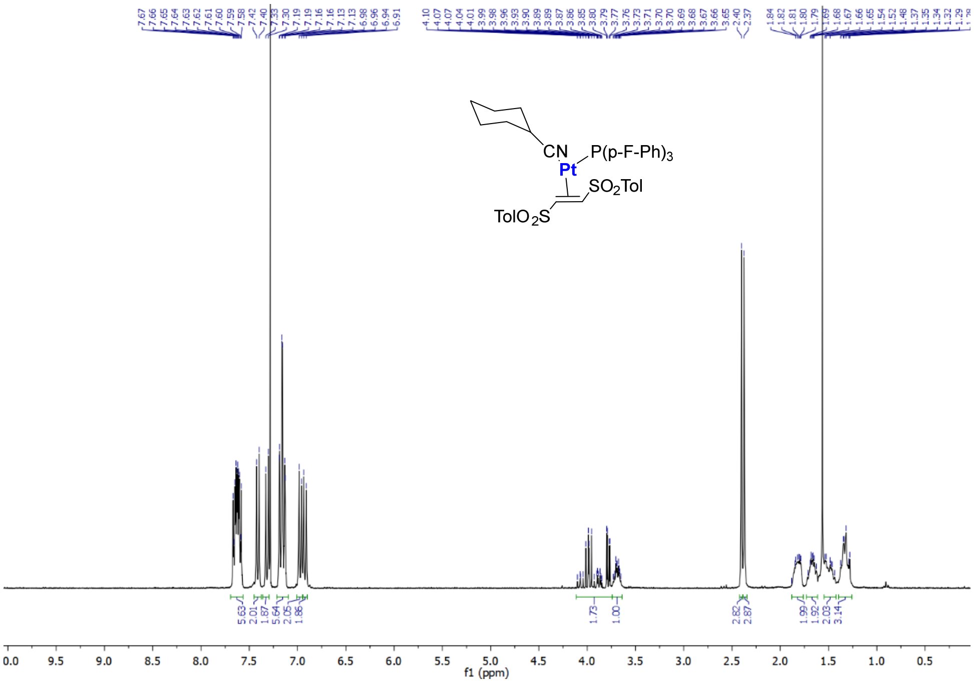

Figure S41. ^{1}H *NMR spectrum of compound* ***4h*** *in* $CDCl_3$ *at 298K*

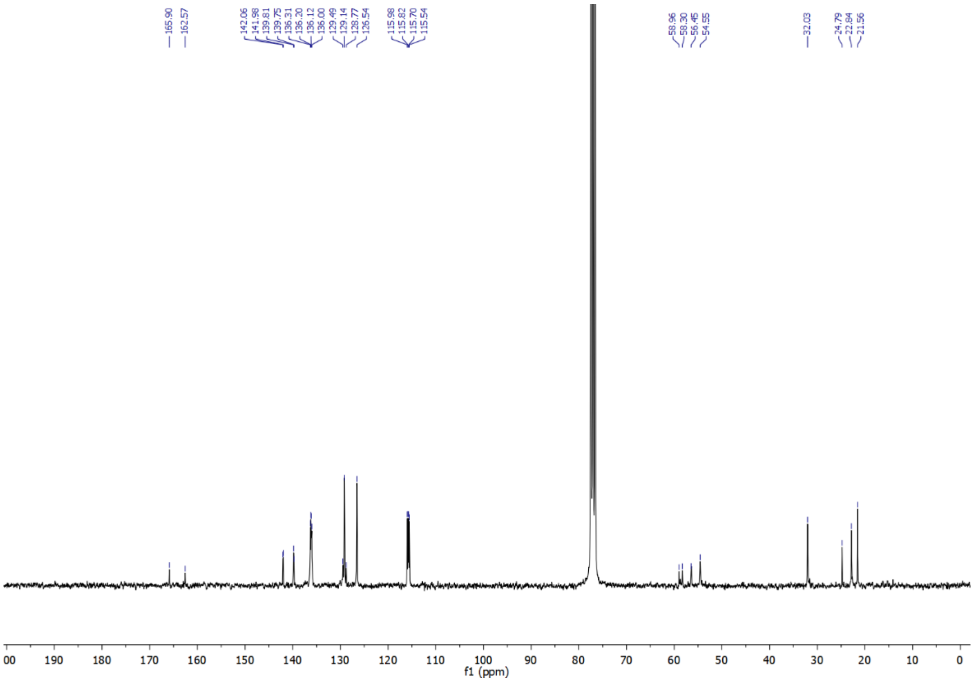

Figure S42. $^{13}C\{^{1}H\}$ *NMR spectrum of compound* ***4h*** *in* $CDCl_3$ *at 298K*

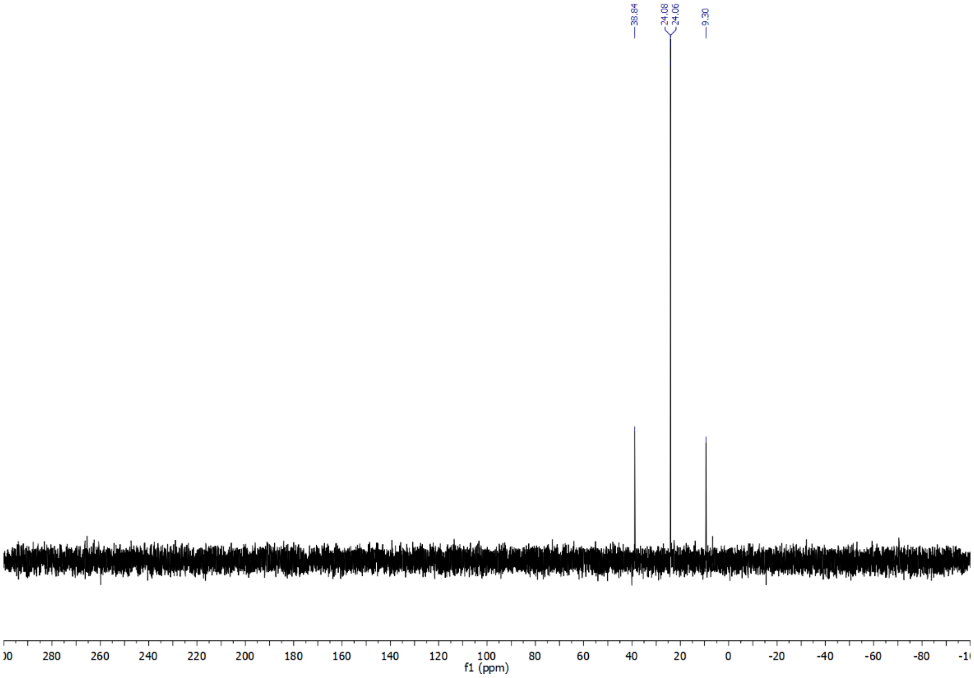

Figure S43. ¹³C{¹H} *NMR spectrum of compound* ***4h*** *in* $CDCl_3$ *at 298K*

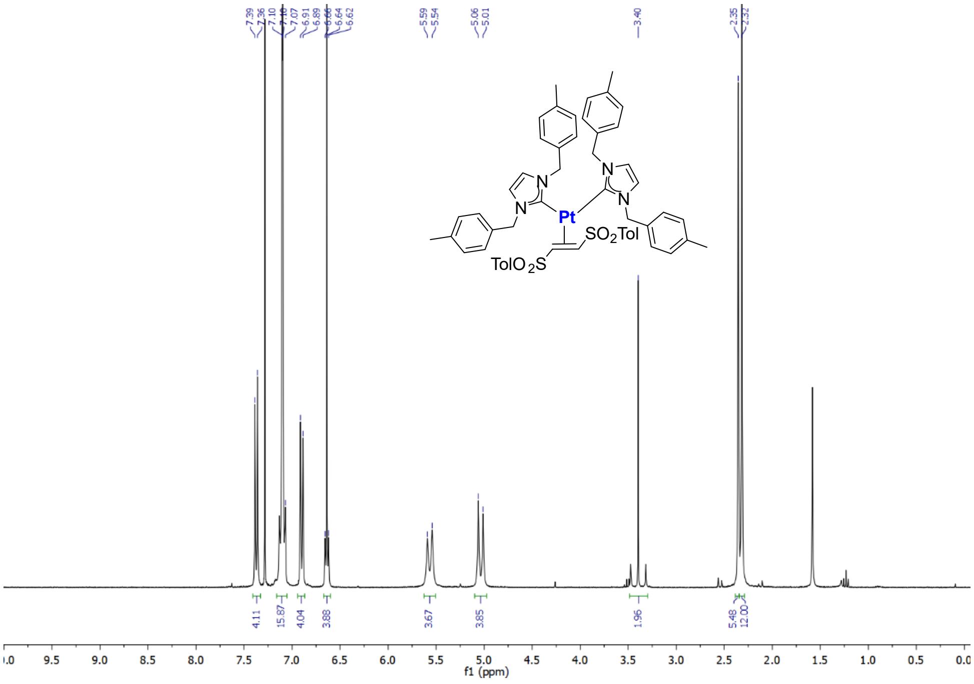

Figure S44. ¹H *NMR spectrum of compound* ***6a*** *in* $CDCl_3$ *at 298K*

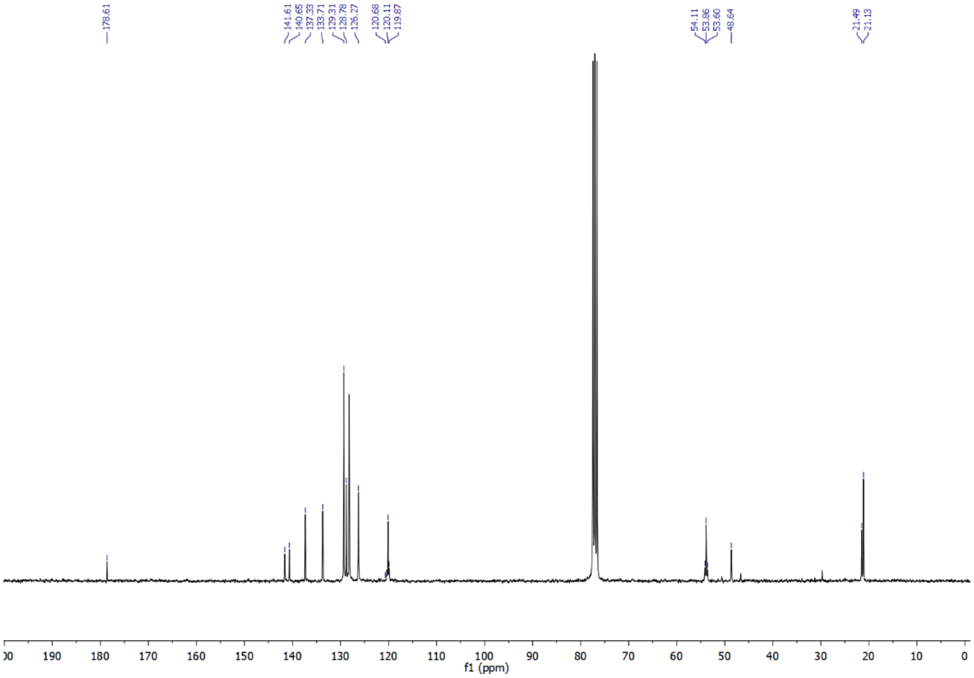

*Figure S45. $^{13}C\{^{1}H\}$ NMR spectrum of compound **6a** in $CDCl_3$ at 298K*

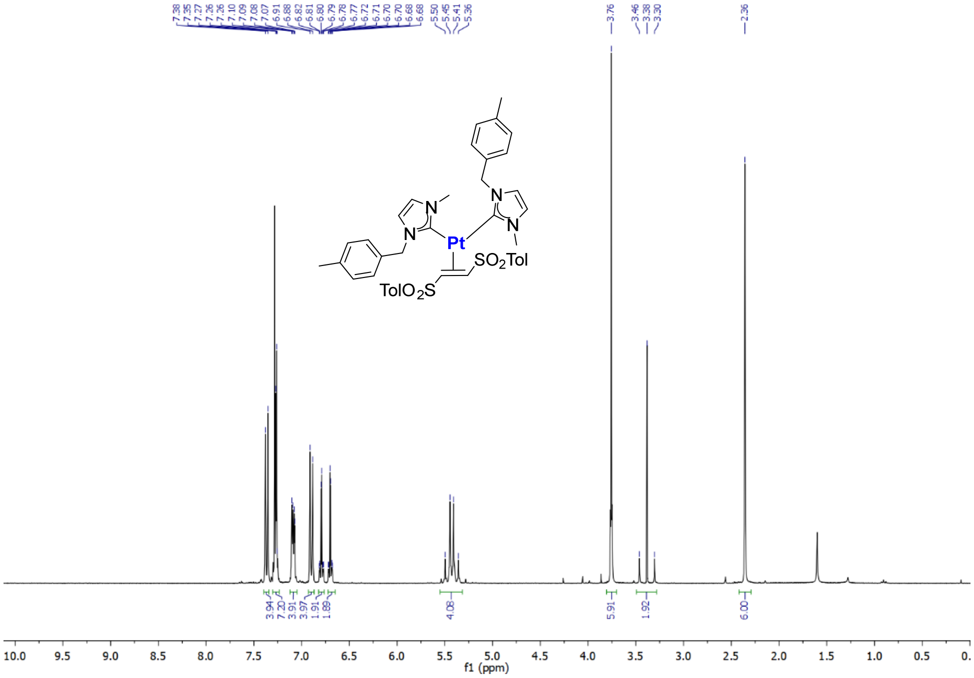

*Figure S46. ^{1}H NMR spectrum of compound **6b** in $CDCl_3$ at 298K*

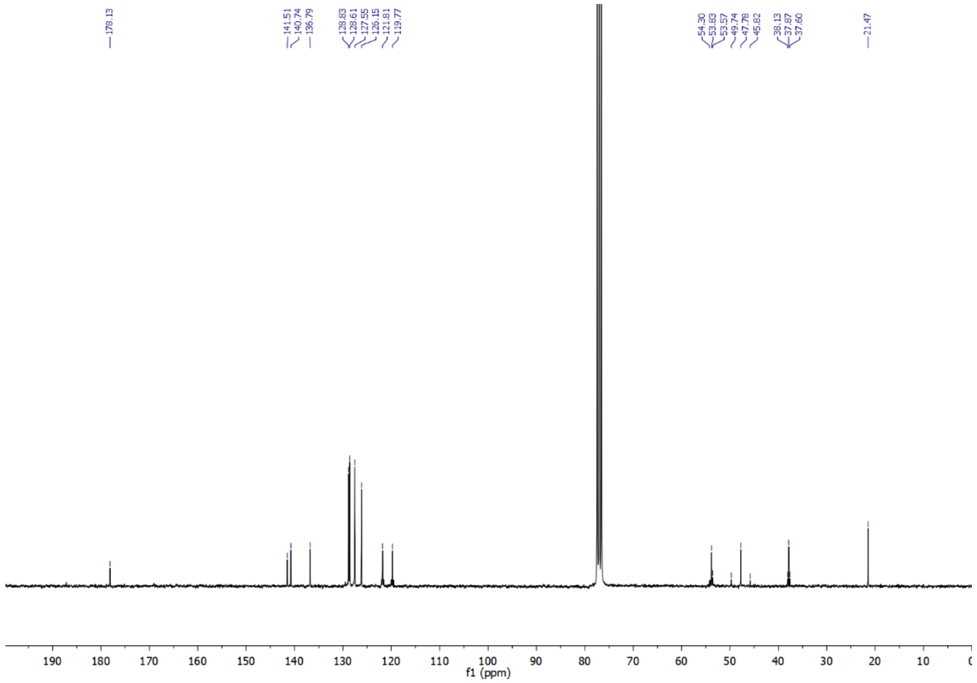

Figure S47. ${}^{13}C\{{}^{1}H\}$ *NMR spectrum of compound* ***6b*** *in* $CDCl_3$ *at 298K*

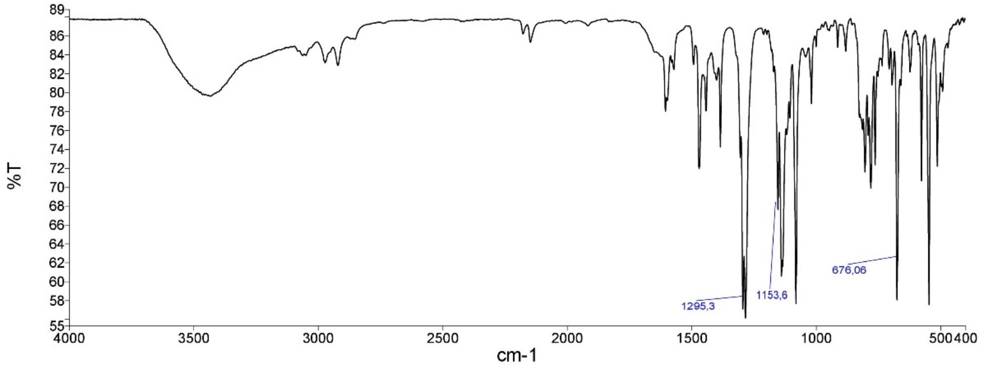

Figure S48. IR spectrum of compound ***1***

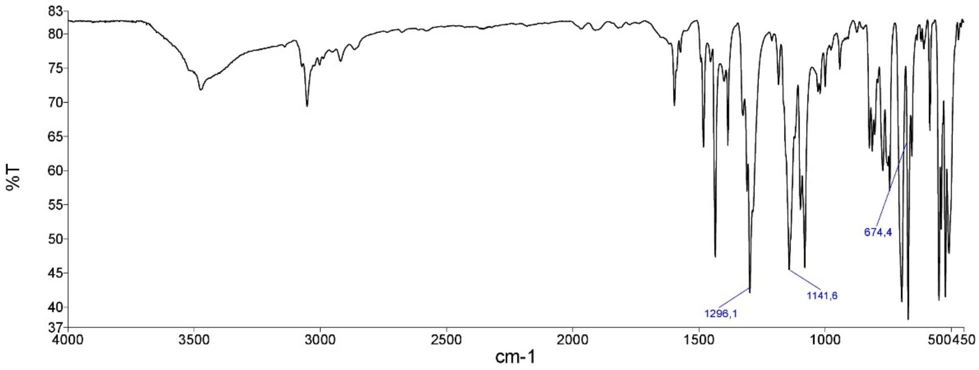

Figure S49. IR spectrum of compound ***2a***

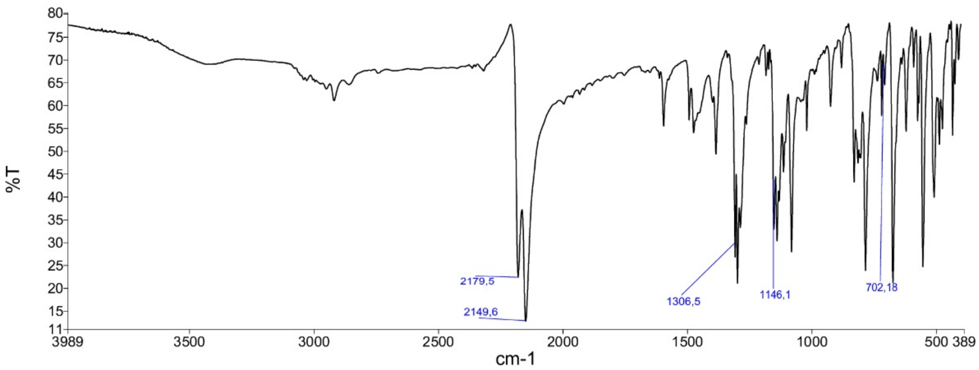

Figure S50. IR spectrum of compound ***3a***

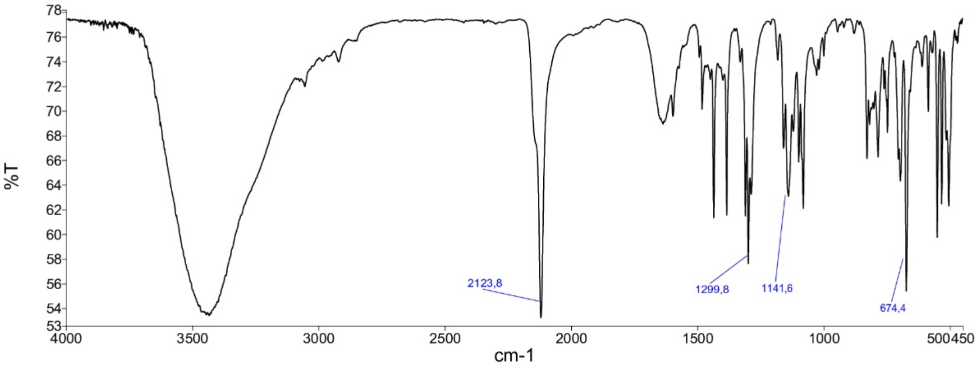

Figure S51. IR spectrum of compound ***4a***

X-ray diffraction analysis - Structural characterization

Table S1. Crystallographic data.

Compound	**3a@100 K**	**3b@100 K**	**2a@100 K**
Formula	$PtC_{34}H_{34}N_2O_4S_2$	$PtC_{34}H_{34}N_2O_8S_4$	$PtC_{52}H_{46}O_4P_2S_2 \cdot {}^1/_4C_4H_{10}O \cdot {}^1/_4CH_2Cl_2$
M/g·mol^{-1}	793.84	921.96	1095.54
Space group	*P* 2$_1$/*c*	*P* -1	*P* -1
Crystal system	Monoclinic	Triclinic	Triclinic
a/Å	15.496(3)	10.295(2)	14.660(3)
b/Å	9.010(2)	13.985(3)	17.981(4)
c/Å	23.067(5)	14.334(3)	18.585(4)
α/°	90	62.54(3)	101.57(3)
β/°	101.56(3)	80.47(3)	96.12(3)
γ/°	90	75.95(3)	98.80(3)
V/Å^3	3155.2(11)	1773.1(8)	4695.1(18)
Z	4	2	4
T/K	100(2)	100(2)	100(2)
D_c/g·cm^{-3}	1.671	1.727	1.550
F(000)	1576	916	2203
μ/mm^{-1}	3.232	2.966	2.251
Measured Reflections	60938	64502	223791
Unique Reflections	10435	12650	41421
R_{int}	0.0397	0.0568	0.0645
Obs. Refl.ns [I≥2σ(I)]	9184	12023	39404
θ_{min}- θ_{max}/°	1.17 – 27.12	1.46 – 28.01	0.99 – 31.13
hkl ranges	-22,22; -13,13; -33,33	-15,15; -21,21; -21,21	-23,23; -29,29; -30,30
R(F^2) (Obs.Refl.ns)	0.0289	0.0215	0.0295
wR(F^2) (All Refl.ns)	0.0767	0.0538	0.0737
No. Variables	394	446	1193
Goodness of fit	1.105	1.038	1.050
$\Delta\rho_{max}$; $\Delta\rho_{min}$/e·Å^{-3}	2.26; -2.39	1.35; -2.52	2.36; -2.22
CCDC Deposition N.	2323081	2323083	2323084

Compound	**2a@298 K**	**4b@100 K**	**6b@100 K**
Formula	$PtC_{52}H_{46}O_4P_2S_2 \cdot {}^1/_4C_4H_{10}O \cdot {}^1/_4CH_2Cl_2$	$PtC_{43}H_{37}F_3NO_4PS_2$	$PtC_{38}H_{40}N_4O_4S_2$
M/g·mol^{-1}	1095.54	978.91	875.95
Space group	*P* -1	*P* 1	*P* -1
Crystal system	Triclinic	Triclinic	Triclinic
a/Å	14.754(3)	11.488(2)	10.808(2)
b/Å	18.270(4)	14.203(3)	11.808(2)
c/Å	18.705(4)	14.404(3)	15.799(3)
α/°	101.72(3)	62.21(3)	74.17(3)
β/°	95.88(3)	75.71(3)	70.02(3)
γ/°	98.88(3)	74.35(3)	76.02(3)
V/Å^3	4830.9(18)	1981.8(9)	1798.5(8)
Z	4	2	2
T/K	298(2)	100(2)	100(2)
D_c/g·cm^{-3}	1.506	1.640	1.618
F(000)	2203	972	876
μ/mm^{-1}	2.188	2.618	2.843
Measured Reflections	232518	70256	17625
Unique Reflections	40702	31365	5731
R_{int}	0.0492	0.0420	0.0476
Obs. Refl.ns [I≥2σ(I)]	34524	29616	5440
θ_{min}- θ_{max}/°	0.98 – 31.83	1.41 – 30.00	1.17 – 27.12
hkl ranges	-24,24; -29,29; -29,29	-18,18; -22,22; -23,23	-12,12; -13,13; -18,18

$R(F^2)$ (Obs.Refl.ns)	0.0343	0.0277	0.0260
$wR(F^2)$ (All Refl.ns)	0.0940	0.0628	0.0636
No. Variables	1150	1000	446
Goodness of fit	1.055	1.109	1.074
$\Delta\rho_{max}$; $\Delta\rho_{min}$/e·Å^{-3}	1.07; -2.53	1.90; -2.40	2.35; -1.56
CCDC Deposition N.	2323085	2323087	2323082

Table S2. Selected platinum distances and angles for **3a** at 100 K.

3a (100 K) - $PtC_{34}H_{34}N_2O_4S_2$

**Distances**	(Å)	**Angles**	(°)
Pt_1-C1_2	2.073(2)	C1_2-Pt_1-C2_2	41.9(1)
Pt_1-C2_2	2.057(2)	C2_2-Pt_1-C_3	107.6(1)
Pt_1-C_3	1.957(3)	C_3-Pt_1-C_4	98.5(1)
Pt_1-C_4	1.962(3)	C1_2-Pt_1-C_4	112.0(1)
C1_2=C2_2	1.476(4)	Pd Ave.- C=C-S Planes[a]	88.4(7)

[a]Average angle among the mean metal coordination plane and the olefinic one (defined as the plane that contains the C=C and non-H atoms directly bound).

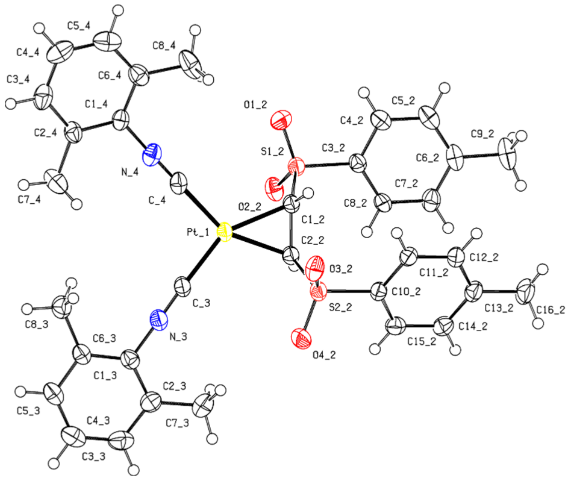

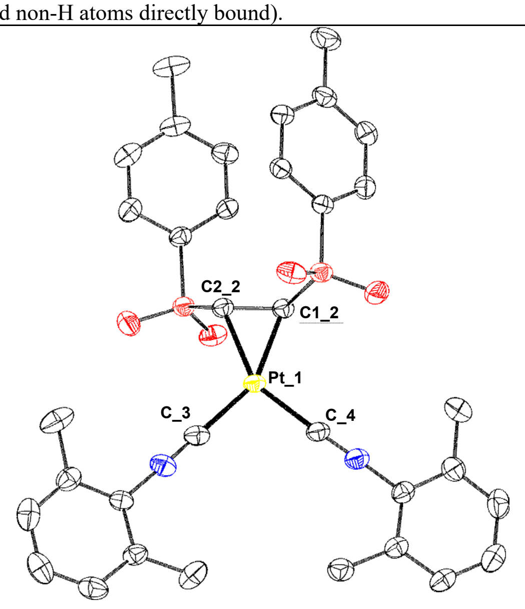

Table S3. Selected platinum distances and angles for **3b** at 100 K.

3b (100 K) - $PtC_{34}H_{34}N_2O_8S_4$

**Distances**	(Å)	**Angles**	**(°)**
Pt_1-C1_2	2.076(2)	C1_2-Pt_1-C2_2	41.38(6)
Pt_1-C2_2	2.060(2)	C2_2-Pt_1-C_3	104.87(7)
Pt_1-C_3	1.948(2)	C_3-Pt_1-C_4	102.90(8)
Pt_1-C_4	1.968(2)	C1_2-Pt_1-C_4	110.80(7)
C1_2=C2_2	1.462(2)	Pd Ave.- C=C-S Planes[a]	89.0(5)

[a]Average angle among the mean metal coordination plane and the olefinic one (defined as the plane that contains the C=C and non-H atoms directly bound).

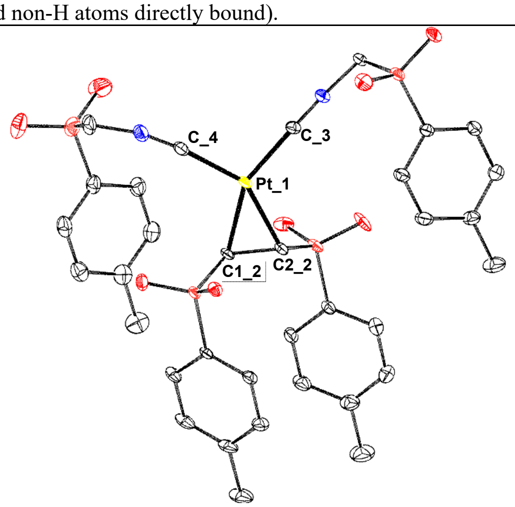

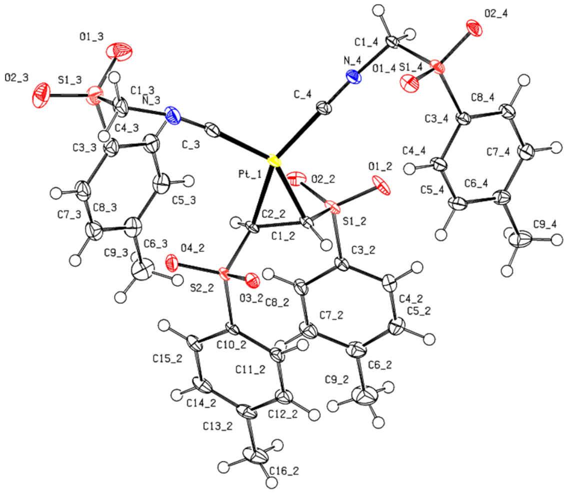

Table S4. Selected platinum distances and angles for **2a** at 100 K.

2a (100 K) - $PtC_{52}H_{46}O_4P_2S_2$

Molecule 1				Molecule 2			
**Distances**	(Å)	**Angles**	(°)	**Distances**	(Å)	**Angles**	(°)
Pt_11-C1_12	2.090(2)	C1_12-Pt_11-C2_12	40.99(8)	Pt_21-C1_22	2.104(2)	C1_22-Pt_21-C2_22	40.85(7)
Pt_11-C2_12	2.070(2)	C2_12-Pt_11-P_13	106.85(6)	Pt_21-C2_22	2.060(2)	C2_22-Pt_21-P_23	101.74(5)
Pt_11-P_13	2.288(1)	P_13-Pt_11-P_14	106.74(2)	Pt_21-P_23	2.284(1)	P_23-Pt_21-P_24	107.8(1)
Pt_11-P_14	2.283(1)	C1_12-Pt_11-P_14	106.21(6)	Pt_21-P_24	2.286(4)	C1_22-Pt_21-P_24	109.8(1)
C1_12=C2_12	1.457(3)	Pd Ave.- C=C-S Planes[a]	89.1(2)	C1_22=C2_22	1.453(2)	Pd Ave.- C=C-S Planes[a]	88.9(4)

[a]Average angle among the mean metal coordination plane and the olefinic one (defined as the plane that contains the C=C and non-H atoms directly bound).

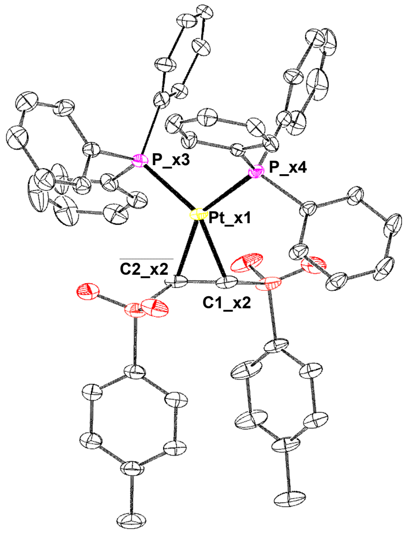

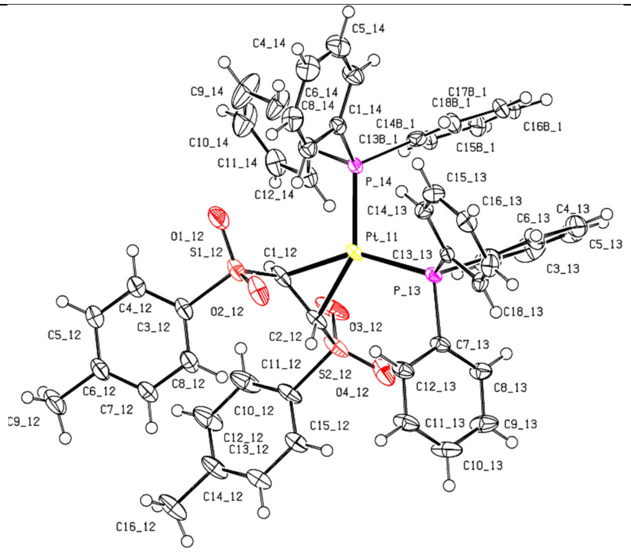

Table S5. Selected platinum distances and angles for **2a** at 298 K.

2a (298 K) - $PtC_{52}H_{46}O_4P_2S_2$

Molecule 1				Molecule 2			
**Distances**	(Å)	**Angles**	(°)	**Distances**	(Å)	**Angles**	(°)
Pt_11-C1_12	2.094(3)	C1_12-Pt_11-C2_12	40.9(1)	Pt_21-C1_22	2.099(2)	C1_22-Pt_21-C2_22	40.88(9)
Pt_11-C2_12	2.067(2)	C2_12-Pt_11-P_13	106.68(7)	Pt_21-C2_22	2.063(2)	C2_22-Pt_21-P_23	102.10(7)
Pt_11-P_13	2.292(1)	P_13-Pt_11-P_14	107.04(3)	Pt_21-P_23	2.290(1)	P_23-Pt_21-P_24	107.8(2)
Pt_11-P_14	2.287(1)	C1_12-Pt_11-P_14	106.30(7)	Pt_21-P_24	2.295(9)	C1_22-Pt_21-P_24	109.6(2)
C1_12=C2_12	1.453(3)	Pd Ave.- C=C-S Planes[a]	89.8(6)	C1_22=C2_22	1.454(3)	Pd Ave.- C=C-S Planes[a]	88.8(7)

[a]Average angle among the mean metal coordination plane and the olefinic one (defined as the plane that contains the C=C and non-H atoms directly bound).

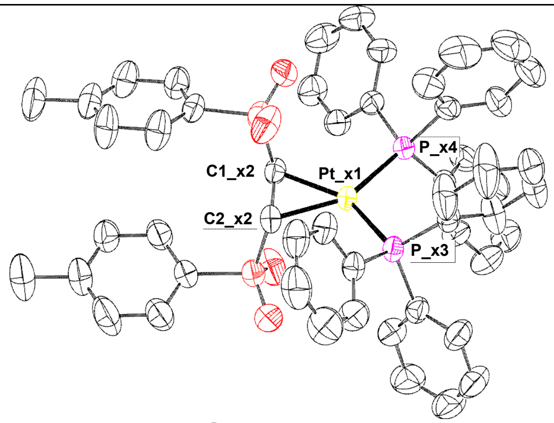

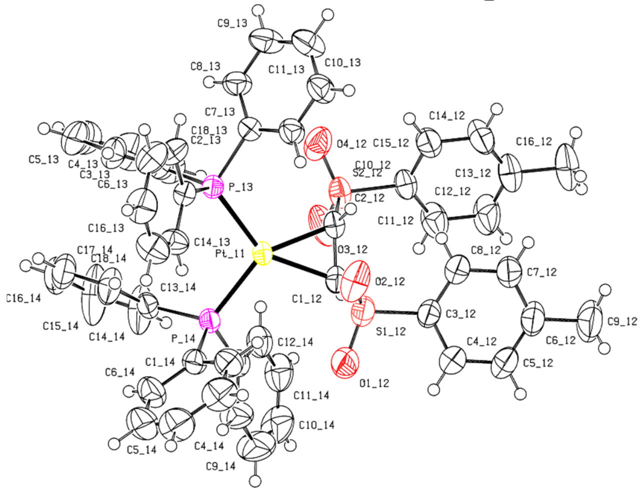

Table S6. Selected platinum distances and angles for **4b** at 100 K.

4b (100 K) - $PtC_{43}H_{37}F_3NO_4PS_2$

Molecule 1				Molecule 2			
**Distances**	(Å)	**Angles**	(°)	**Distances**	(Å)	**Angles**	(°)
Pt_11-C1_12	2.073(4)	C1_12-Pt_11-C2_12	41.2(2)	Pt_21-C1_22	2.077(3)	C1_22-Pt_21-C2_22	41.2(2)
Pt_11-C2_12	2.081(4)	C2_12-Pt_11-C_13	108.9(2)	Pt_21-C2_22	2.078(4)	C2_22-Pt_21-C_23	109.8(2)
Pt_11-C_13	1.942(3)	C_13-Pt_11-P_14	101.5(1)	Pt_21-C_23	1.941(3)	C_23-Pt_21-P_24	99.1(1)
Pt_11-P_14	2.305(1)	C1_12-Pt_11-P_14	108.5(1)	Pt_21-P_24	2.289(1)	C1_22-Pt_21-P_24	110.5(1)
C1_12=C2_12	1.461(5)	Pd Ave.- C=C-S Planes[a]	87.2(6)	C1_22=C2_22	1.462(5)	Pd Ave.- C=C-S Planes[a]	83.5(3)

[a]Average angle among the mean metal coordination plane and the olefinic one (defined as the plane that contains the C=C and non-H atoms directly bound).

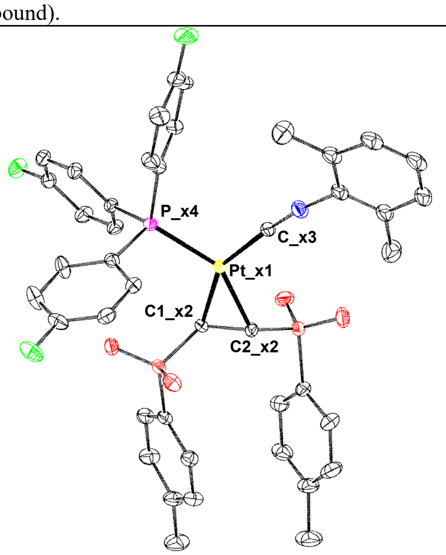

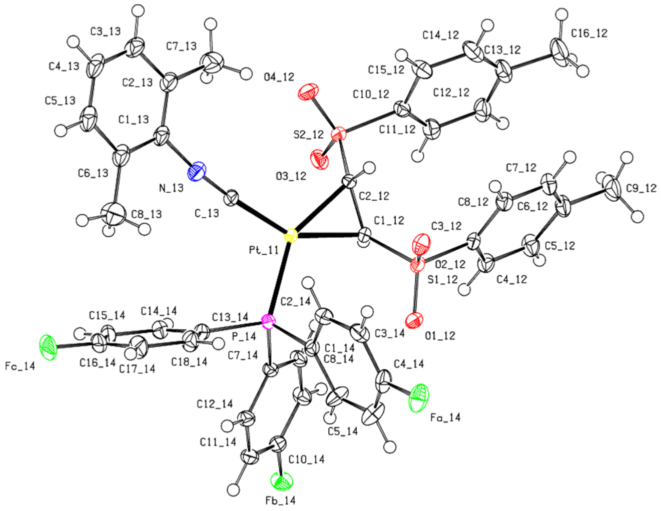

Table S7. Selected platinum distances and angles for **6b** at 100 K.

6b (100 K) - $PtC_{38}H_{40}N_4O_4S_2$

**Distances**	(Å)	**Angles**	(°)
Pt_1-C1_2	2.064(5)	C1_2-Pt_1-C2_2	42.0(2)
Pt_1-C2_2	2.078(5)	C2_2-Pt_1-C1_4	109.2(2)
Pt_1-C1_3	2.020(5)	C1_3-Pt_1-C1_4	99.3(2)
Pt_1-C1_4	2.007(4)	C1_2-Pt_1-C1_3	109.5(2)
C1_2=C2_2	1.486(5)	Pd Ave.- C=C-S Planes[a]	86.2(3)
		Pd Ave.- NHC Planes[b]	73.8(3)

[a]Average angle among the mean metal coordination plane and the olefinic one (defined as the plane that contains the C=C and non-H atoms directly bound).
[b]Average angle among the mean metal coordination plane and the NHC ones (defined as the plane that contains the C=C and non-H atoms directly bound).

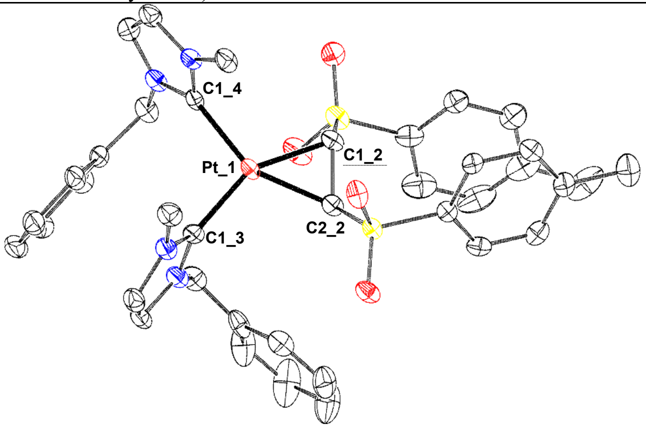

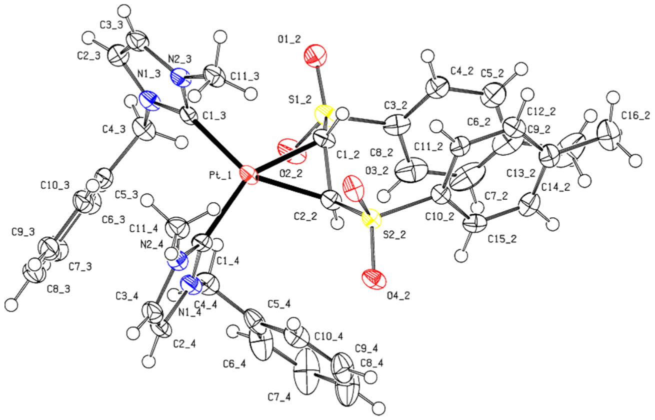

Supplement: Supplementary file 1 [file molecules-29-01119-s001.zip › ESI.pdf]
